# α-Synuclein blocks endoplasmic reticulum co-translational protein translocation early in Parkinson’s disease

**DOI:** 10.1038/s41467-026-76173-4

**Published:** 2026-08-06

**Authors:** Chor Lai Lam, Nicholas J. F. Gatford, Ana Aragón-González, Benedict Tanudjojo, Anis Sahoo, Andrew R. Castle, Devika Agarwal, Ashwin Jainarayanan, Svenja S. Hester, Navoneel Sen, Justin L. P. Benesch, Roman Fischer, David Sims, George K. Tofaris

**Affiliations:** 1 Nuffield Department of Clinical Neurosciences, University of Oxford, Oxford, UK; 2 Kavli Institute for Nanoscience Discovery, University of Oxford, Oxford, UK; 3 MRC Weatherall Institute of Molecular Medicine, University of Oxford, Oxford, UK; 4 Kennedy Institute of Rheumatology, University of Oxford, Oxford, UK; 5 Target Discovery Institute, Nuffield Department of Medicine, University of Oxford, Oxford, UK; 6 Department of Chemistry, University of Oxford, Oxford, UK

**Keywords:** Cellular neuroscience, Parkinson's disease, Endoplasmic reticulum, Induced pluripotent stem cells, Protein-protein interaction networks

## Abstract

The primary mechanism and subcellular localisation of α-synuclein toxicity in Parkinson’s disease pathogenesis remain unknown. We spatially and temporally resolved proteomic and transcriptomic changes in human iPSC-derived dopaminergic neurons with increasing burden of pathological α-synuclein. We found that misfolded α-synuclein proteoforms, signified by the formation of nanoscale intraneuronal puncta, are associated with impaired translocon function at the endoplasmic reticulum (ER). We show that α-synuclein interacts with Sec61A in iPSC-derived dopaminergic neurons and in post-mortem brain tissue from patients with Parkinson’s disease. This interaction interferes with the co-translational translocation of ER-processed proteins including the vacuolar-type ATPase V0a1 subunit, glucocerebrosidase, and Cathepsin B, causing defective organelle function such as reduced lysosomal acidification, leading to increased extracellular vesicle release of α-synuclein. Defective ER-translocation was associated with increased ribosomal UFMylation and proteasomal recruitment but not activation of the unfolded protein response. Reduction of pathological α-synuclein by either CRISPRi to decrease α-synuclein expression or pharmacological activation of proteasomal degradation with repurposed drugs mitigates the ER defect. Our study offers a unifying mechanistic link between α-synuclein pathology and dysregulation of diverse organelle-associated proteins that are both Sec61A translocon substrates and genetic modifiers of Parkinson’s disease risk. Our data also provide a therapeutic rationale for proteasomal activation in early Parkinson’s disease.

## Abstract (web summary)

Proteomic and transcriptomic profiling on hiPSC-derived midbrain dopaminergic neurons with pathological α-synuclein burden reveals defective Sec61A interactions and co-translational translocation of ER-processed proteins.

## Introduction

The cardinal motor features of Parkinson’s disease (PD) are caused by the degeneration of midbrain dopaminergic neurons (DANs), which is widely ascribed to intraneuronal accumulation and misfolding of α-synuclein (αSyn)^1^. This is further supported by familial PD cases harbouring αSyn gene (*SNCA*) multiplications where gene dosage correlates with disease severity^2^. Successful modelling of PD pathology in cells or animals using αSyn overexpression or seeded aggregation with exogenous fibrils has identified multiple potential mechanisms of its proteotoxicity^1^. We previously developed a human model of αSyn pathology using induced pluripotent stem cell (iPSC)- derived dopaminergic neurons (hiPSC-DANs) and found that αSyn abundance and conformation are key determinants of pathology^3^. Despite the description of aberrant aggregate interactions and diverse resulting phenotypes, a temporal and spatial resolution of the process of αSyn aggregation and its impact on human DANs is currently lacking. Such in-depth information is needed to fully delineate the sequence of the earliest events that ultimately damage human neurons and to identify subcellular pathways of molecular convergence for therapeutic intervention. Here, we deployed data-driven interrogation of such events derived from spatially and temporally resolved proteomic and transcriptomic changes in hiPSC-derived DANs with increasing burden of pathological αSyn by overexpression, αSyn gene (*SNCA)* triplication (*SNCA*^*TRIP*^), *SNCA* A53T mutation or seeded aggregation. Specifically, we used proximity labelling with ascorbate peroxidase (APEX)^4^ in living neurons at different stages of pathology followed by mass spectrometry and time- or single cell-resolved transcriptome analyses to delineate the earliest mechanisms of αSyn-induced neurotoxicity.

## Results

### Aberrant αSyn interactions in human DANs associate with nanoscale aggregates

**Fig. 1:**
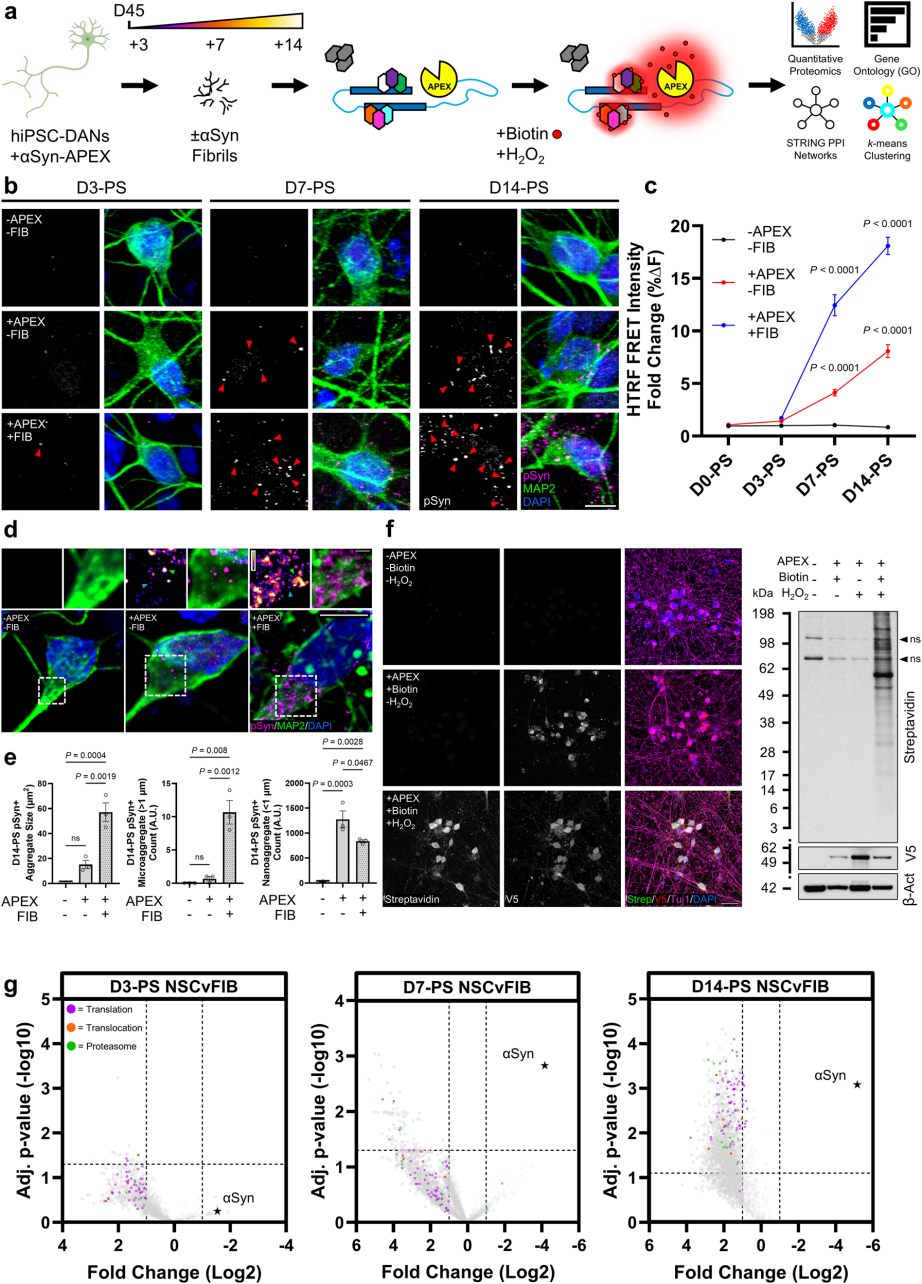
Nanoscale aggregates are associated with aberrant αSyn interactions in human DANs. **a** Schematic detailing the αSyn-APEX interactome experimental pipeline with and without αSyn seeding. Image Created with BioRender https://BioRender.com/699uz0t. **b** Representative images showing progressive pSyn staining in hiPSC-DANs overexpressing αSyn-APEX with and without seeded αSyn aggregation over time. Scale bar = 5 μm. **c** HTRF FRET intensity data showing increasing pSyn aggregation in hiPSC-DANs overexpressing αSyn-APEX with and without seeded αSyn aggregation (±FIB) over time. Two-way ANOVA—*F*(10,33) = 168.2, *p* < 0.0001; D3-PS|D14-PS *p* < 0.0001, *n* = 3. **d** Representative iSIM images showing that αSyn-APEX overexpression induces nanoscale aggregates in hiPSC-DANs. Upper scale bar = 1 μm, lower scale bar = 5 μm. **e** Quantification of pSyn+ aggregate size, micro- or nanoaggregate count showing that seeding with αSyn fibrils induces microaggregates while αSyn-APEX overexpression is sufficient to induce nanoaggregates Size: Ordinary one-way ANOVA—*F*(2,6) = 38.05, *p* = 0.0004; αSyn-APEX-|Fibril+: *p* = 0.0019. Microaggregate count: Ordinary one-way ANOVA—*F*(2,6) = 33.24, *p* = 0.0006; αSyn-APEX-|Fibril+: *p* = 0.0012. Nanoaggregate count: Ordinary one-way ANOVA—*F*(2,6) = 40.86, *p* = 0.0003; αSyn-APEX-|Fibril+: *p* = 0.047, *n* = 3. **f** Representative images and immunoblot showing αSyn-APEX induced biotinylation only in the combined presence of αSyn-APEX overexpression, biotin treatment and hydrogen peroxide (H_2_O_2_), *n* = 4. Scale bar = 100 μm. Immunoblot arrows indicate non-specific (ns) bands found in all hiPSC-DAN control samples probed for streptavidin. **g** Volcano plots showing the complete αSyn-APEX interactome at D3-post seeding (PS), D7-PS and D14-PS with and without seeded aggregation (FIB) in hiPSC-DANs, *n* = 4. Purple dots indicate translation related hits. Orange dots indicate proteins involved in ER co-translational translocation. Green dots indicate proteasome related hits. Highlighted dots were targeted for downstream analysis. Star indicates the bait protein αSyn on all volcano plots. Vertical and horizontal dashed lines indicate cutoffs of log_2_ fold change (+1/−1) and adjusted –log_10_ *P* value (1.3), respectively. Differentially abundant proteins were identified using a student’s *t*-test using a permutation-based FDR correction (5%). Each dot corresponds to one clone differentiated once and data are mean ± SEM from at least *n* = 3 differentiations per clone. Source data are provided as a Source Data file.

To map the αSyn interactome during the process of aggregation we transduced DANs derived from a healthy hiPSC line with a lentivirus expressing WT αSyn-APEX (Fig. 1a). The neurons were differentiated using a modified floor-plate protocol^5^, transduced upon plating on day in vitro 25 (D25) and matured to D45, a timepoint at which we previously found hiPSC-derived DANs to be electrophysiologically active^5^. We first confirmed that DANs co-expressed microtubule-associated protein 2 (MAP2) (76.46%), β−3 tubulin (TUJ1) (74.19%), tyrosine hydroxylase (TH) (70.42%) and dopamine transporter (DAT) (62.38%) (Supplementary Fig. 1a). We then confirmed that transduction had minimal effect on viability or differentiation efficiency: ~70% were TUJ1-expressing cells averaged across all transduction conditions (Supplementary Fig. 1b).

To reproduce different stages of pathology we generated de novo fibrils (Supplementary Fig. 2) and confirmed their seeding capacity in control hiPSC-DANs (Supplementary Fig. 3). Half of the APEX-transduced hiPSC-DANs were then seeded at D45 and monitored at 3-, 7- and 14-days post-seeding (D3-PS, D7-PS, D14-PS) up to D60. Under these conditions, immunostaining revealed progressive pathology as detected by phosphorylation of αSyn at serine 129 (pSyn), which is the most reliable and widely used marker of endogenous αSyn aggregation^6^. Airyscan super-resolution microscopy showed that αSyn-APEX expression alone led to the formation of aggregates which increased further upon seeding with αSyn fibrils (Fig. 1b). This progressive increase in aggregation was further confirmed by homogenous time-resolved fluorescence (HTRF) FRET-based quantification of pSyn signal in neuronal lysates at each time point as shown in Fig. 1c. Instant structured illumination microscopy (iSIM) confirmed that nanoscale aggregates (≤1 μm) are detected when αSyn-APEX per se was expressed in hiPSC-DANs (Fig. 1d and quantified in 1e). These data demonstrate that αSyn-APEX expression ± αSyn fibrils in hiPSC-DANs simulates different stages of αSyn aggregation in a human neuronal model.

To identify interactions within a 10 nm radius of misfolded αSyn assemblies, we induced protein biotinylation in living human DANs by adding 500 μM biotin-phenol for 30 min, followed by 1 min of 1% H_2_O_2_. In control experiments, we first tested and confirmed successful αSyn-APEX induced biotinylation in DANs by both immunostaining and immunoblotting (Fig. 1f). DANs were seeded at D45 and biotinylation was induced at three timepoints post-seeding (PS): D3-PS, D7-PS and D14-PS. Interacting proteins were immunoprecipitated from neuronal lysates using streptavidin-coated beads as confirmed by immunoblotting (Supplementary Fig. 4), eluted, and subjected to mass spectrometry. We identified a combined total of 2674 proteins with a (log2)fold change of ≤ or ≥ 1 between non-seeded and seeded aggregation across all conditions. Volcano plots of all αSyn interacting proteins at different timepoints revealed an unexpected asymmetry in the interactome with an increasing number of interacting proteins reaching statistical significance over time under non-seeded conditions (Fig. 1g and Supplementary Data 1). Strikingly, the principal differentially interacting protein detected under seeded aggregation relative to the non-seeded condition was αSyn itself, most prominently at D14-PS, in contrast to 1716 interacting proteins detected in the non-seeded condition. No biotinylated proteins were detected in the remaining pellets when dissolved in 2% SDS, thus excluding partial solubilisation of aggregates as the likely explanation for this finding (Supplementary Fig. 5). Therefore, in our experimental model, larger αSyn-APEX ‘microscale’ aggregates or inclusion bodies induced by seeding appear to be comparatively biochemically inert and primarily biotinylate self and neighbouring αSyn molecules but not other proteins in their vicinity. Instead, most interactions were detected with αSyn-APEX overexpression, which induced the formation of ‘nanoscale’ aggregates as shown in Fig. 1d, e. Collectively, these data show that early-stage αSyn aggregates (≤1 μm) rather than larger inclusions are associated with more extensive aberrant interactions within neurons.

### αSyn interactome analysis implicates the ER and proteasomes in early pathology

**Fig. 2:**
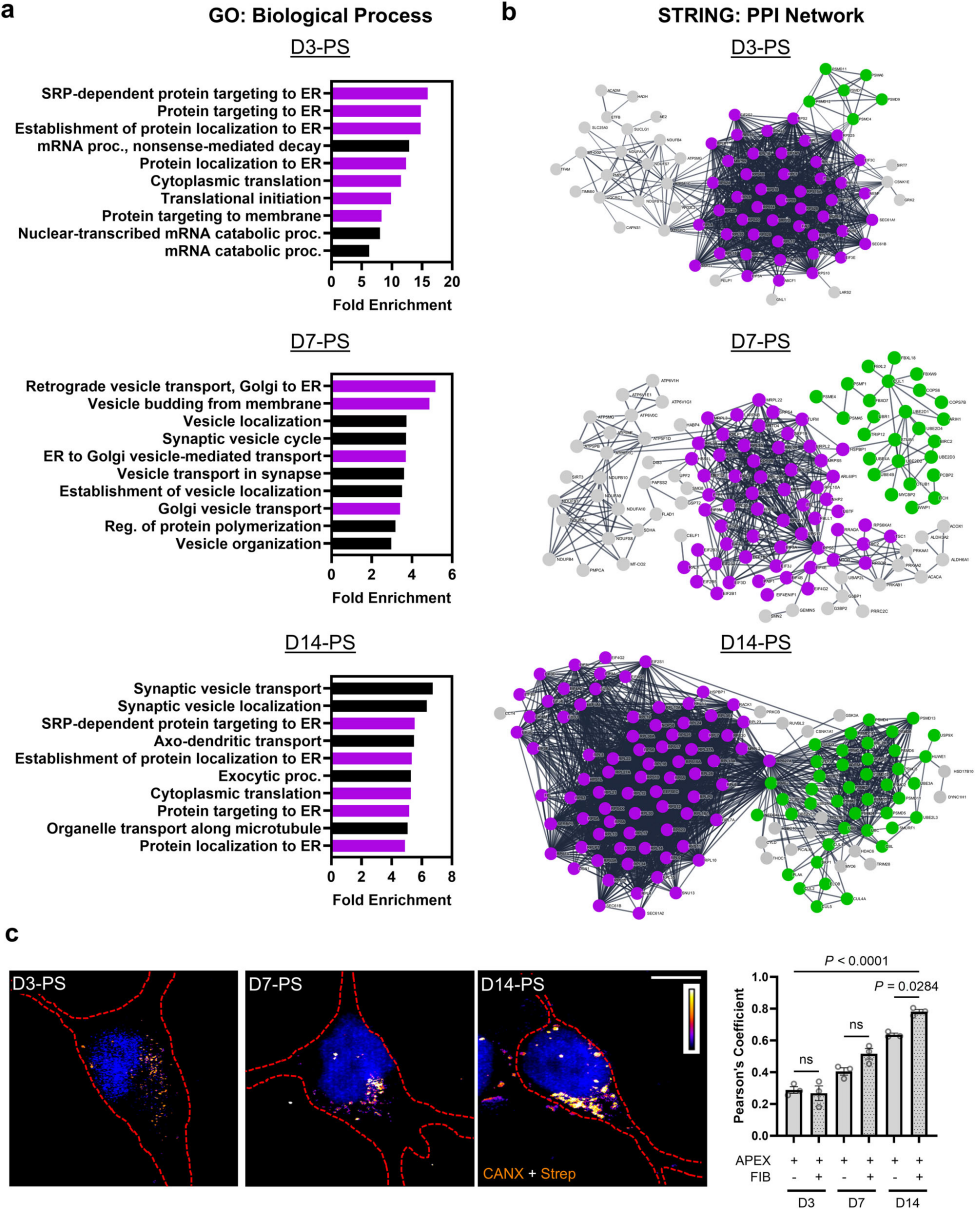
αSyn interactome analysis implicates the ER and proteasomes in early pathology. **a** Gene ontology (GO) analysis showing the top 10 terms corresponding to biological process across time in the αSyn interactome. Purple highlights indicate ER related GO terms. **b** STRING-db protein-protein interaction (PPI) networks showing the most densely clustered interactions after *k*-means clustering of the overall PPI network across time in the αSyn interactome. Purple highlights indicate ER related proteins. Green highlights indicate proteasome related proteins. **c**, Representative images and data of combined calnexin and streptavidin signal demonstrating increased colocalization intensity between the ER and biotinylated proteins over time in hiPSC-DANs with and without αSyn seeded aggregation. Two-way ANOVA—*F*(6,12) = 107.2, *p* < 0.0001,; D3-PS+-|D14-PS++: *p* < 0.00013, *n* = 3. Scale bar = 5μm. Each dot corresponds to one clone differentiated once and data are mean ± SEM from at least *n* = 3 differentiations per clone. Source data are provided as a Source Data file.

To determine which pathways were mostly perturbed, we performed Gene Ontology (GO) and STRING protein-protein interaction (PPI) network analysis. This analysis identified ribosomal-ER translation as the core component of the early αSyn interactome (purple in all volcano plots in Fig. 1g and GO analyses/PPI networks in Fig. 2a, b), localising to the translocon based on the identification of two of its three subunits (SEC61A and SEC61B) as well as components of the signal recognition particle (SRP) including SRP14, SRP68, SRP72 and SRPRB (orange in all volcano plots in Fig. 1g). Specifically, at D3-PS, multiple ER translation-related terms were among the top 10 enriched pathways including SRP-dependent protein targeting to the ER as shown in Fig. 2a. These terms were persistent at D7-PS and D14-PS with additional enrichment in ER-Golgi vesicle transport, organelle and synaptic vesicle localisation. Further *k*-means clustering of the overall D14-PS PPI network indicated that these terms were driven by two highly dense and connected subclusters consisting of many ribosomal-ER proteins (including SEC61A and SEC61B) and many proteasomal subunits or ubiquitin pathway proteins (Fig. 2b). This map of the time-resolved αSyn-induced interactome suggests a progressive disruption of ER-associated translocation of nascent chains followed by proteasome recruitment at this site of damage. We confirmed the co-localisation between streptavidin (denoting biotinylated proteins) and calnexin (ER marker) which was already detected in αSyn-APEX expressing hiPSC-DANs with only a small increase upon seeding, most notably at D14-PS (Fig. 2c). Collectively, these data identify the ER as a key focus of pathology in hiPSC-DANs that is already detectable when early misfolded αSyn proteoforms start to accumulate.

### The transcriptomic response to *SNCA*^*TRIP*^ in DANs is enriched in ER and translation terms

**Fig. 3:**
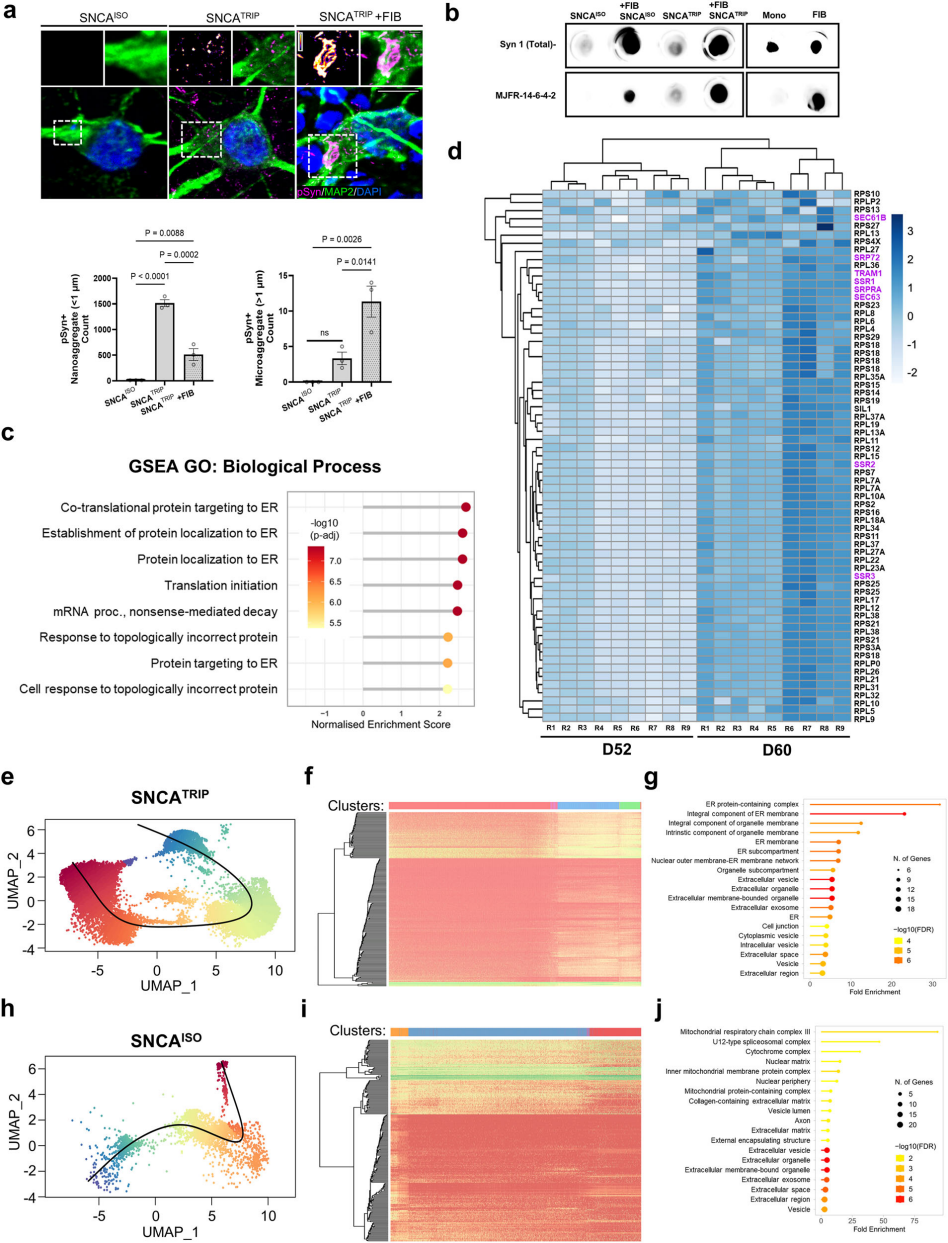
The transcriptomic response to *SNCA*^*TRI*P^ in DANs is enriched in ER and translation terms. **a** Representative iSIM images and quantification showing endogenous pSyn-positive nanoaggregates in *SNCA*^*TRIP*^ compared to *SNCA*^*ISO*^ hiPSC-DANs which coalesce into microaggregates upon seeding with fibrils. Upper scale bar = 1 μm, lower scale bar = 5 μm. Size: Microaggregate count: Ordinary one-way ANOVA—*F*(2,6) = 18.32, *p* = 0.0028; *SNCA*^*TRIP*^|*SNCA*^*TRIP*^+FIB: *p* = 0.014, *n* = 3. Nanoaggregate count: Ordinary one-way ANOVA—*F*(2,6) = 99.20, *p* < 0.0001; *SNCA*^*ISO*^|*SNCA*^*TRIP*^*:* *p* < 0.0001, *SNCA*^*TRIP*^|*SNCA*^*TRIP*^ + FIB: *p* = 0.0002, *n* = 3. **b** Representative native dot blot showing detection of misfolded αSyn in *SNCA*^*TRIP*^ DANs, but not in *SNCA*^*ISO*^ in non-seeded conditions using MJFR-14-6-4-2. Syn1 recognises total αSyn. Aggregation was highest in dot blots of lysates from seeded (FIB) *SNCA*^*TRIP*^ and seeded *SNCA*^*ISO*^ DANs. Antibody specificity of MJFR-14-6-4-2 for aggregated αSyn is shown with recombinant monomers (Mono) vs fibrils (FIB). **c** Top 8 Gene Ontology (GO) terms identified by bulk RNA-seq using Gene Set Enrichment Analysis (GSEA) with a significance threshold at adjusted *p* < 0.001 were enriched in biologically relevant pathways that differed at D52 vs D60 hiPSC-DANs suggesting a progressive impact of αSyn on ER-associated translation. DESeq2 Wald test was used for differential expressions; *p*-values were corrected for multiple testing using Benjamini-Hochberg False discovery rate (FDR) method. **d** Heatmap showing differentially expressed genes between D52 vs D60 hiPSC-DANs for the top pathway (co-translational protein targeting to ER; *n* = 9 per timepoint). ER-related genes are shown in purple. **e** Pseudotime trajectory in *SNCA*^*TRIP*^ hiPSC-DANs with **f** corresponding heatmap depicting the expression profile of the genes influencing the pseudotime trajectories across the five DAN clusters and **g** the GO cellular component analysis of these genes. **h** Pseudotime trajectory in *SNCA*^*ISO*^ hiPSC-DANs with **i** corresponding heatmap depicting the expression profile of the genes influencing the trajectory across the identified DAN clusters and **j** the GO cellular component analysis of these genes. Each dot corresponds to one clone differentiated once and data are mean ± SEM from at least *n* = 3 differentiations per clone. Source data are provided as a Source Data file.

αSyn-APEX overexpression in hiPSC-derived DANs is an accelerated model of aggregation involving a fused construct that may introduce artefacts. To orthogonally assess the relevance of our αSyn interactome in a pathophysiologically relevant system, we generated DANs from hiPSCs derived from a patient with *SNCA*^*TRIP*^, which we previously showed to exhibit approximately a 2.5-fold increase in αSyn expression^3^. We first tested whether these neurons exhibit early-stage aggregates that may not be easily detectable. To this end, we performed iSIM which revealed nanoscale aggregates (≤1 μm) in *SNCA*^*TRIP*^ hiPSC-DANs while fibril seeding increased the preponderance of microscale (>1 μm) aggregates (Fig. 3a). This finding was further confirmed by dot blot under native conditions, which revealed misfolded αSyn in *SNCA*^*TRIP*^ DANs but not isogenic control DANs (Fig. 3b) using the MJFR14-6-4-2 antibody at a dilution (1:20000) that selectively binds to aggregates^7^.

To map the evolution of pathology caused by endogenously generated misfolded αSyn proteoforms in *SNCA*^*TRIP*^ hiPSC-DANs, we initially performed bulk RNA-seq on hiPSC-DAN neuronal cultures and temporally assessed the transcriptomic changes between DANs at D52 vs D60 (*n* = 9 per timepoint), two time-points which roughly corresponded to the days in culture investigated in the αSyn-APEX system. DESeq2 with log2 fold-change >0.5 and adjusted *p*-value of <0.05 identified 4383 differentially expressed genes between these timepoints. In total, 1973 genes were upregulated and 2410 genes were downregulated in DANs at D52 vs D60 (Supplementary Data 2). To decipher progressive cellular responses, we performed Gene Set Enrichment Analysis (GSEA) using GO in D52 vs D60. Similar to our proteomic data, with a threshold of adjusted *p* < 0.001 we found enrichment in ER-associated functions and ribosomal translation with co-translational protein targeting to the ER and establishment of protein localisation to the ER being the top pathways (Fig. 3c). Upregulated genes in the leading edge of the top pathway (Fig. 3d) included the translocon component *SEC61B*, the transmembrane ER channel *TRAM1* required for protein translocation across the ER membrane, the translocon-associated subunit SSR1, the SRP receptor subunit alpha (*SRPRA*), *SEC63* and ribosomal subunits (*RPL17*, *RPL36*, *RPL12*), suggesting an attempt to compensate for a progressive defect in these complexes.

To further confirm these changes, we performed single cell RNA sequencing comparing DANs derived from a separate *SNCA*^*TRIP*^ clone to its isogenic control clone (*SNCA*^*ISO*^) at D73. After preprocessing, we identified five clusters of DANs which showed differential gene expressions (Fig. 3e and Supplementary Fig. 6). Pseudotime analysis was conducted to explore the functional trajectories within these clusters. This analysis revealed a distinct trajectory between the clusters in *SNCA*^*TRIP*^ DANs compared to their isogenic controls, suggesting a transition across pathological states (Figs. 3e and 3h). To elucidate the molecular underpinnings of these phenotypic differences, we identified differentially expressed genes (DEGs) in *SNCA*^*TRIP*^ and isogenic control DANs across the pseudotime trajectory (Fig. 3f, i and Supplementary Data 3). GO analysis was then applied to the differentially expressed genes within these trajectories. The results showed significant enrichment in terms associated with ER-associated protein complex, ER membrane, organelle subcompartments and extracellular vesicles (EVs), highlighting these pathways as key drivers of the transitional states observed in *SNCA*^*TRIP*^ DANs (Fig. 3g), which were distinct from those detected in isogenic controls (Fig. 3j). These temporally resolved transcriptome changes both at bulk and single cell resolution further support the identification of ER as a focus of early pathology.

### αSyn interacts with Sec61A but not the other main ER translocases

**Fig. 4:**
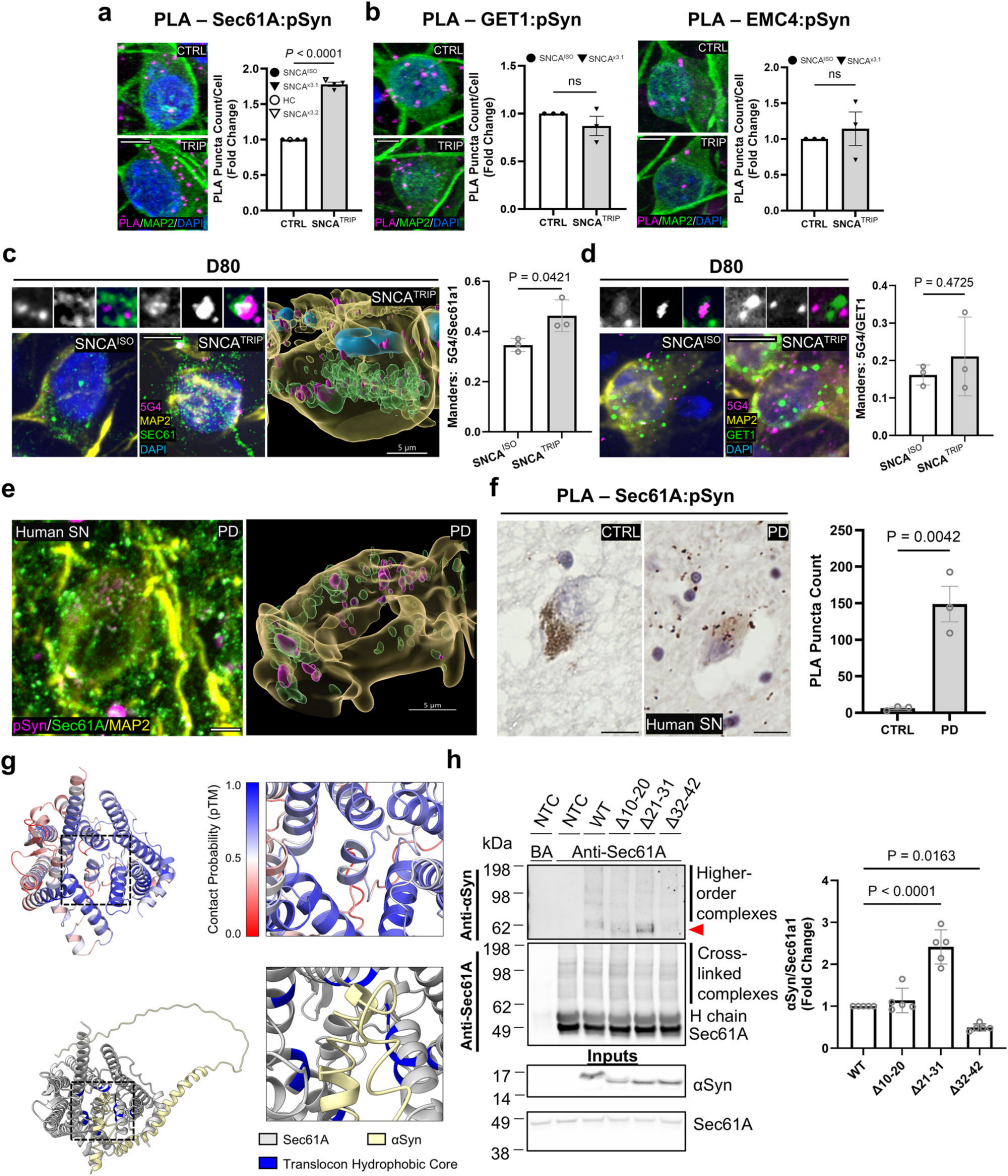
αSyn interacts with the ER translocase Sec61A1. **a** Representative images and quantification of proximity ligation assay (PLA) demonstrating increased pSyn interaction with Sec61A in *SNCA*^*TRIP*^ hiPSC-DANs when compared to healthy controls at D80. D80 Sec61: pSyn; CTRL|*SNCA*^*TRIP*^: Two-sided Unpaired *t*-test—t(12) = 25.69, *p* < 0.0001. Scale bar = 5 μm, *n* = 4. **b** Representative images and quantification of PLA showing no change in the interaction between pSyn and TMEM85 (EMC4) or GET1. D80 GET1: pSyn; CTRL|*SNCA*^*TRIP*^ Two-sided Unpaired *t*-test—t(12) = 1.281, *p* = 0.2694. D80 EMC4: pSyn; CTRL|*SNCA*^*TRIP*^ t(12) = 0.6047, *p* = 0.55780, *n* = 3. Scale bar = 5 μm. **c** Representative images with 3D reconstruction and quantification of co-localisation between Sec61 and 5G4-labelled αSyn, showing increased co-localisation in *SNCA*^*TRIP*^ hiPSC-DANs when compared to isogenic control DANs. Sec61: 5G4; *SNCA*^*ISO*^|*SNCA*^*TRIP*,^ Two-sided Unpaired *t*-test—*t*(4) = 2.948, *p* = 0.0421, *n* = 3. Scale bar = 5 μm. **d** Representative image and quantification of co-localisation between GET1 and 5G4-labelled αSyn, showing no difference in *SNCA*^*TRIP*^ hiPSC-DANs compared to isogenic controls DANs, GET1: 5G4; *SNCA*^*ISO*^|*SNCA*^*TRIP*^, Two-sided Unpaired *t*-test; t(4) = 0.7924, *p* = 0.4725, *n* = 3. Scale bar = 5 μm. **e** Representative image with 3D reconstruction showing co-localisation between Sec61 and pSyn in substantia nigra sections from postmortem PD brain. **f** Representative images and quantification of PLA demonstrating increased pSyn interaction with Sec61A in substantia nigra sections from postmortem PD brains compared to healthy controls. Sec61A: pSyn; *CTRL*|*PD*, Two-sided Unpaired *t*-test; t(4) = 5.890, *p* = 0.0042, *n* = 3 case per group. Scale bar = 5 μm. **g** Structural modelling shows the likely sites of interaction between Sec61A and a predicted αSyn proteoform with a partly unfolded N-terminal α-helical domain. **h** Immunoblots and corresponding quantification of immunoprecipitation showing interaction between αSyn (WT and N-terminus deletion mutants) with Sec61A following in vivo crosslinking (0.5 mM DSG). The red arrow indicates the band corresponding to the Sec61A–αSyn complex (monomeric αSyn), Ordinary one-way ANOVA—F(3,16) = 51.83, *p* < 0.0001; αSyn-WT|αSyn-∆21–31: *p* < 0.0001. Scale bar = 5 μm; αSyn-WT|αSyn-∆32-42: *p* = 0.0163, *n* = 5. Each dot corresponds to one clone differentiated once, or one biological replicate and data are mean ± SEM from at least *n* = 3 differentiations per clone. Source data are provided as a Source Data file.

To confirm the identified interactions and impact on ER-associated translation in an endogenous system, we performed PLA between pSyn, which denotes pathological αSyn and the translocon subunit Sec61A in hiPSC-DANs with *SNCA*^*TRIP*^ (labelled SNCA^X3.1^), their isogenic hiPSC DANs (*SNCA*^*ISO*^) as well as hiPSC-DANs derived from a separate *SNCA*^*TRIP*^ hiPSC line (labelled SNCA^X3.2^) and a healthy control (HC) hiPSC line. In agreement with the interactome results, we found that non-seeded *SNCA*^*TRIP*^ hiPSC-DANs exhibit increased interaction between pSyn and Sec61A when compared to controls at D80 (Fig. 4a). In contrast, other ER-associated complexes involved in protein translocation such as the EMC and GET pathways did not co-localise with αSyn based on similar background PLA signal in *SNCA*^*TRIP*^ vs *SNCA*^*ISO*^ between pSyn and either TMEM85 (EMC4) or GET1 epitopes that are exposed to the cytosol (Fig. 4b). In further control experiments, we tested pSyn and UGP2, a protein that was identified as a non-significant interactor in the proteomic analysis, as well as pSyn with no additional antibody. Under these control conditions, limited amplification of equal intensity was seen in *SNCA*^*ISO*^ and *SNCA*^*TRIP*^ hiPSC-DANs (Supplementary Fig. 7a, b). Furthermore, we assessed the abundance and localisation of misfolded αSyn proteoforms as detected by 5G4, an antibody that preferentially recognises such species^8^. We found evidence of co-localisation of 5G4-positive proteoforms with Sec61A but not GET1 in D80 *SNCA*^*TRIP*^ hiPSC-DANs when compared to their isogenic control (Fig. 4c, d). We assessed the relevance of these findings to the human condition by examining substantia nigra sections from postmortem PD or control brains (*n* = 3 per group). We immunostained for Sec61A and pSyn and visualised their interaction by 3D image rendering (Fig. 4e and Supplementary Fig. 8c) and PLA (Fig. 4f and Supplementary Fig. 8a, b). These experiments showed increased interaction between pSyn and Sec61A only in PD brains.

Collectively, these experiments are consistent with αSyn-induced blockade of the translocon subunit Sec61A, suggesting a mechanism of proteotoxicity in human neurons. We therefore sought to generate a structural model of this interaction. AlphaFold3^9^ predicted that αSyn assemblies do not directly interact with Sec61A (Supplementary Fig. 9a). We then asked whether non-assembled αSyn proteoforms may interact with Sec61A. Although AlphaFold often produces structures with low confidence for intrinsically disordered proteins, we were interested to obtain a structure of the complex between Sec61A and αSyn. We used ColabFold^10^, which combines the fast homology search of MMseqs2 with AlphaFold2, to increase our sampling of the conformational space by using multiple seeds. The structural predictions generated from 100 runs were ranked using the estimated template modelling score (pTM). For each structure, the minimum distance between Sec61A and αSyn for each residue on αSyn were calculated and the weighted average distance for the top 50 structures was calculated using the pTM scores as weights. This distance, normalised between 0 and 1, is displayed on the Sec61A structure (Fig. 4g), with regions coloured in blue being closest in space to αSyn and regions in red furthest away. With a 4.5 Å cutoff, this analysis revealed the Sec61A residues that are closest to αSyn are all hydrophobic: Phe12, Ile15, Leu89, Leu93, Ile122, Ile125, Phe156, Leu293, Phe374, Phe375, Tyr378. Notably, two of these residues (Leu89 and Leu293) overlap with the hydrophobic core of Sec61A (Fig. 4g), which associates with nascent chains as they emerge from the ribosome^11^. The N-terminus of a predicted partially unfolded α-helix of αSyn (amino acids 16-40), which is enriched in Lys residues, is the most likely sequence interacting with the Sec61A hydrophobic core residues.

To assess this model experimentally, we expressed WT or deletion mutants (Δ10-20, Δ21-31, Δ32-42) in the N-terminus, tagged with Flag at the C-terminus, in SH-SY5Y cells in which endogenous αSyn was knocked out with recombinant Cas9 (Supplementary Fig. 9b). In this model, we co-immunoprecipitated the WT protein and αSyn deletion mutants with Sec61A after crosslinking with DSG (0.5 mM) and immunocapture with anti-Sec61A antibodies. We found that αSyn Δ32-42 deletion consistently decreased the interaction with Sec61A (*n* = 5). Interestingly the αSyn Δ21-31 deletion mutant exhibited enhanced interaction with Sec61A compared to WT (Fig. 4h). This could be due to re-arrangement of key lysine residues in amino acids 32-42, within the N-terminus of αSyn, which has an imperfect tandem repeat sequence with a KTK core (Supplementary Fig. 9c). Our findings indicate that changes in the N-terminus of αSyn influence its interaction with Sec61A. Therefore, unfolding of the N-terminal helical structure of αSyn may induce mislocalisation and interaction with Sec61A, blocking access to its hydrophobic core that anchors newly synthesised polypeptide chains. This model concurs with our proteomic analysis, which identified fewer interaction events under seeding conditions that induce the formation of larger assemblies.

### αSyn misfolding interferes with ER co-translational translocation and activates UFMylation

**Fig. 5:**
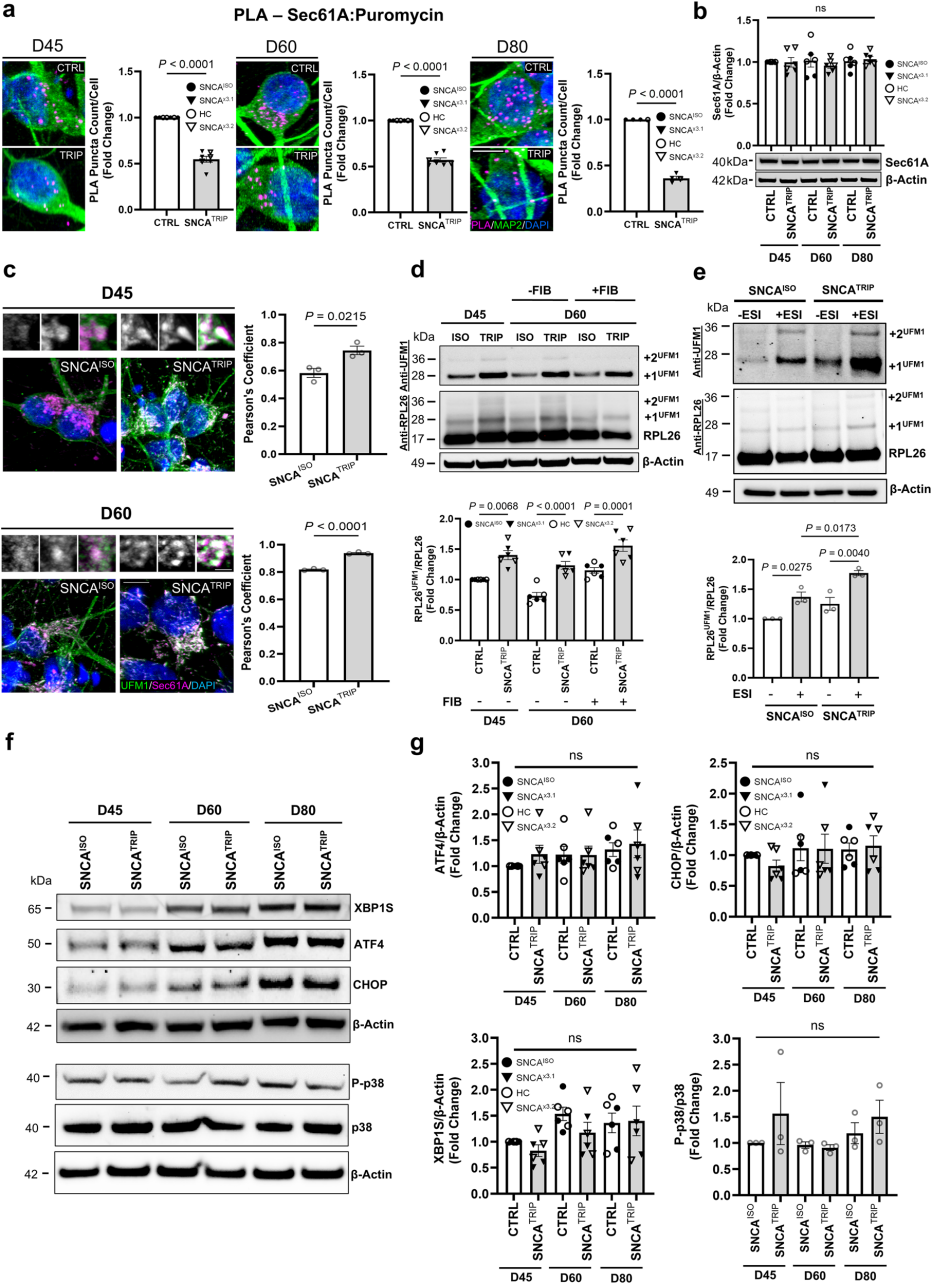
αSyn interferes with ER co-translational translocation and activates UFMylation. **a** Representative images and quantification of PLA demonstrating reduced interaction of Sec61A with puromycin-labelled nascent chains in *SNCA*^*TRIP*^ hiPSC-DANs when compared to healthy controls at all tested timepoints. D45 CTRL|*SNCA*^*TRIP*^: Two-sided Unpaired *t*-test—t(12) = 14.08, *p* < 0.0001. D60 CTRL|*SNCA*^*TRIP*^: Two-sided Unpaired *t*-test—t(12) = 18.35, *p* < 0.0001. D80 CTRL|*SNCA*^*TRIP*^: Two-sided Unpaired *t*-test—t(12) = 29.32, *p* < 0.0001, *n* = 6. Scale bar = 5 μm. **b**, Representative immunoblots and quantification showing equal Sec61A expression across CTRL and *SNCA*^*TRIP*^ hiPSC-DANs at all timepoints. Two-way ANOVA—*F*(5,25) = 0.43, *p* = 0.82, *n* = 6. **c** Representative images and quantification showing increased colocalization of UFM1 with Sec61A *SNCA*^*TRIP*^ when compared to *SNCA*^*ISO*^ control hiPSC-DANs at D45 and D60. D45 *SNCA*^*ISO*^|*SNCA*^*TRIP*^: Two-sided Unpaired *t*-test—t(4) = 3.67, *p* = 0.02. D60 *SNCA*^*ISO*^|*SNCA*^*TRIP*^: Two-sided Unpaired *t*-test—t(4) = 18.97, *p* < 0.0001. *n* = 3. Scale bar = 1 μm (upper), 5 μm (lower). **d** Representative immunoblots and quantification showing increased UFMylation in *SNCA*^*TRIP*^ hiPSC-DANs compared to *SNCA*^*ISO*^ controls over time. Two-way ANOVA—*F*(5,25) = 48.75, *p* < 0.0001; D45 CTRL|*SNCA*^*TRIP*^: *p* < 0.0001, D60 CTRL|*SNCA*^*TRIP*^: *p* ≤ 0.0001, D80 CTRL|*SNCA*^*TRIP*^: *p* = <0.0001, *n* = 3. **e** Representative immunoblots and quantification showing increased RPL26 UFMylation in Eeyarestatin I (ESI) treated *SNCA*^*ISO*^ and *SNCA*^*TRIP*^ hiPSC-DANs, with treated *SNCA*^*TRIP*^ exhibiting higher levels compared to treated *SNCA*^*ISO*^ hiPSC-DANs. One-way ANOVA -F(3,8) = 20.07, *p* = 0.0004, *SNCA*^*ISO*^|*SNCA*^*ISO*^ ESI, *p* = 0.0275, *SNCA*^*TRIP*^|*SNCA*^*TRIP*^ ESI, *p* = 0.004, *SNCA*^*ISO*^ ESI|*SNCA*^*TRIP*^ ESI, *p* = 0.0173, *n* = 3. **f** Representative immunoblots showing p38 phosphorylation (*n* = 3 differentiations) and unfolded protein response effectors (*n* = 6) over time between *SNCA*^*ISO*^ and *SNCA*^*TRIP*^ hiPSC-DANs. **g** Quantification of immunoblot showing no difference in p38 phosphorylation (*n* = 3) and unfolded protein response (*n* = 6) over time between *SNCA*^*ISO*^ and *SNCA*^*TRIP*^ hiPSC-DANs. ATF4 (One-way ANOVA -F(5,30) = 0.6902, *p* = 0.6347),CHOP (One-way ANOVA -F(5,30) = 0.6150, *p* = 0.6892), XBP1 (One-way ANOVA -F(5,29) = 2.184, *p* = 0.0835), P-38 (One-way ANOVA -F(5,12) = 0.9651, *p* = 0.4763). Each dot corresponds to one clone differentiated once and data are mean ± SEM from at least *n* = 3 differentiations per clone. Source data are provided as a Source Data file.

To investigate the functional consequences of αSyn/Sec61A interaction, we pulsed hiPSC-DANs with 10 μg/ml of puromycin for 10 min. Puromycin is a tyrosyl-tRNA mimic that blocks translation by labelling and releasing elongating polypeptide chains from translating ribosomes. By combining puromycin treatment with PLA between Sec61A and puromycin-labelled nascent chains as they are released from ribosomes, we aimed to measure co-translational translocation into the ER in *SNCA*^*TRIP*^ hiPSC-DANs (clone SNCA^x3.1^) compared to their isogenic control (SNCA^ISO^) hiPSC-DANs. To ensure that any findings were not an idiosyncrasy of this isogenic hiPSC pair, we also used an additional *SNCA*^*TRIP*^ line (SNCA^x3.2^) and a hiPSC line from a healthy control (HC). We found that *SNCA*^*TRIP*^ hiPSC-DANs exhibit a 2-fold decrease in ER-associated translation at D45 and D60 which was more pronounced at D80 (Fig. 5a) i.e. the opposite to the pSyn/Sec61A signal (Fig. 4a). In control experiments, neuronal lysates from puromycin-treated cultures were assessed by immunoblotting, which confirmed equal Sec61A expression levels across hiPSC lines (Fig. 5b) and the incorporation of puromycin into nascent chains, identified as a smear due to variable polypeptide length (Supplementary Fig. 10a). These data strongly suggest that endogenously generated pathological αSyn conformers interact and interfere with ER-associated translation.

Impaired translation at the ER triggers ribosome subunit UFMylation with UFM1 to regulate the degradation of stalled polypeptide chains on the ribosome^12,13^. We therefore assessed the co-localisation of UFM1 with Sec61A in *SNCA*^*TRIP*^ and isogenic control hiPSC-DANs at D60. We found increased co-localisation between UFM1 and Sec61A in *SNCA*^*TRIP*^ hiPSC-DANs as shown in Fig. 5c. This co-localisation was associated with increased total UFMylation or UFMylation of the Sec61A associated ribosomal subunit RPL26^14^ in neuronal lysates of *SNCA*^*TRIP*^ hiPSC-DANs compared to controls as quantified by immunoblotting (Fig. 5d). Overnight treatment with the Sec61A inhibitor Eeyarestatin (ESI; 20 μM) further increased UFMylation in *SNCA*^*TRIP*^ hiPSC-DANs compared to controls (Fig. 5e). At the timepoints tested, there was no increase in p38 phosphorylation (Fig. 5f and 5g), which has been linked to apoptosis induced by stalling of cytoplasmic translation^15^, or activation of the unfolded protein response (UPR) (Fig. 5f, g). In control experiments we confirmed that increased UFMylation occurs in cells in response to ribosomal stalling induced by treatment with the peptidyl transferase inhibitor Anisomycin (200 nM) over time (Supplementary Fig. 10b).

### Defective co-translational translocation into the ER impairs the delivery of organelle-associated proteins

**Fig. 6:**
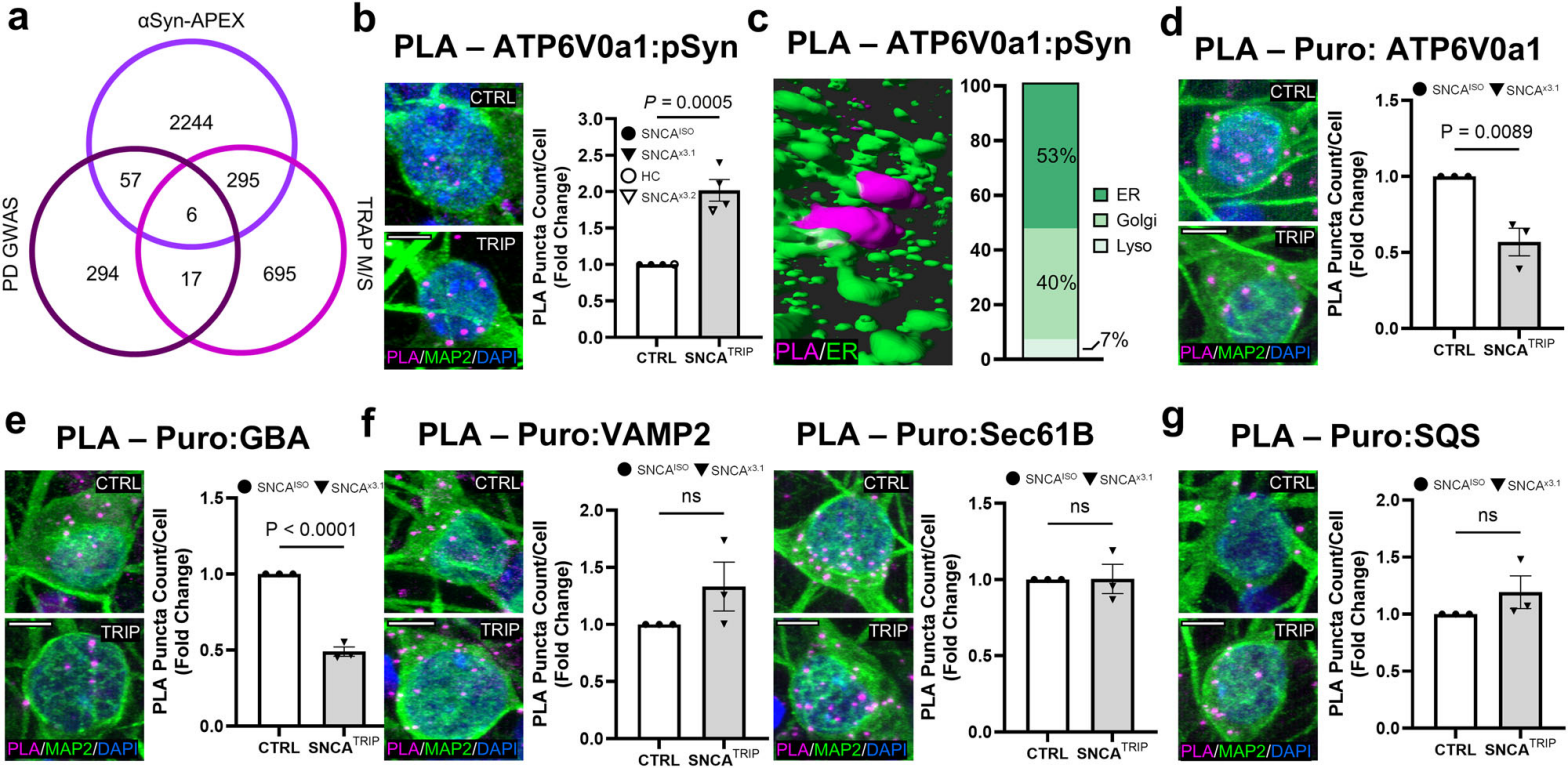
Defective co-translational translocation into the ER impairs the delivery of organelle-associated proteins. **a** Venn diagram showing the overlap of hits from the αSyn-APEX interactome, PD-associated genes and previously identified translocon substrates^16^ including ATP6V0a1. **b** Representative images and quantification of PLA between ATP6V0a1 and pSyn showing increased interaction in *SNCA*^*TRIP*^ hiPSC-DANs compared to healthy controls at D80. CTRL|*SNCA*^*TRIP*^: Two-sided Unpaired *t*-test—t(12) = 6.84, *p* = 0.0005, *n* = 6. Scale bar = 5 μm. **c** Representative 3D render image showing PLA puncta from ATP6V0a1: pSyn interaction in contact with the ER (left) with quantification of the percentage of contact sites between PLA puncta and the ER, Golgi apparatus and endolysosomes (right). **d** Representative images and quantification of PLA between puromycin-labelled nascent chains and Sec61A substrate ATP6V0a1 and **e** GBA showing a decrease in interaction in *SNCA*^*TRIP*^ hiPSC-DANs compared to healthy controls at D80. Puro: ATP6V0a1 CTRL|*SNCA*^*TRIP*^: Two-sided Unpaired *t*-test—t(12) = 4.760, *p* = 0.0089. Puro:GBA CTRL|*SNCA*^*TRIP*^: Two-sided Unpaired *t*-test—t(12) = 16.40, *p* < 0.0001, *n* = 3. Scale bar = 5 μm. **f** Representative images and quantification of PLA between puromycin-labelled nascent chains and GET substrate Sec61B and VAMP2 showing no difference in interaction in *SNCA*^*TRIP*^ hiPSC-DANs compared to healthy controls at D80. Puro: Sec61B CTRL|*SNCA*^*TRIP*^: Two-sided Unpaired *t*-test—t(12) = 0.04879, *p* = 0.9634. Puro: VAMP2 CTRL|*SNCA*^*TRIP*^: Two-sided Unpaired *t*-test—t(12) = 1.545, *p* = 0.197, *n* = 3. Scale bar = 5 μm. **g** Representative images and quantification of PLA between puromycin-labelled nascent chains and EMC substrate SQS showing no difference in interaction in *SNCA*^*TRIP*^ hiPSC-DANs compared to healthy controls at D80. *SNCA*^*ISO*^|*SNCA*^*TRIP*^: Two-sided Unpaired *t*-test—t(12) = 1.340, *p* = 0.2513, *n* = 3. Scale bar = 5 μm. Each dot corresponds to one clone differentiated once and data are mean ± SEM from at least *n* = 3 differentiations per clone.

ER-associated ribosomal translation concerns primarily the synthesis and distribution of proteins that are essential for organelle and plasma membrane homoeostasis. We found that ~30% of all proteins identified as translocon substrates^16^ were labelled by αSyn-APEX including proteins implicated in PD by genetic studies (Fig. 6a). One explanation for this finding is that biotinylation occurs during polypeptide chain synthesis at the ER and predicts that the ER-associated translational defect in *SNCA*^*TRIP*^ hiPSC-DANs impacts on the trafficking of translocon substrates. We tested this prediction focusing initially on the localisation of the v-ATPase V0a1 subunit of the v-ATPase pump because it is required for widespread organelle pH homoeostasis and it is also a PD GWAS candidate gene^17^. To determine where pathological pSyn interacts with v-ATPase V0a1, we quantified the contact sites between the PLA signal arising from these two aforementioned markers and Sec61A- (ER), GM130- (Golgi) or Lamp2- (Endolysosome) positive 3D-rendered structures. We found that the pSyn/v-ATPase V0a1 PLA signal was increased in *SNCA*^*TRIP*^ neurons compared to isogenic controls (Fig. 6b) and primarily detected in contact with the ER (52.63%) or Golgi (40.26%) and to a much lesser extent with endolysosomes (7.11%) as shown in Fig. 6c even though the v-ATPase V0a1 is normally most abundant at the lysosome^18^. Although we cannot exclude direct interaction with the assembled v-ATPase at these organelles, our data in *SNCA*^*TRIP*^ hiPSC-DANs suggest that pathological αSyn primarily interacts with v-ATPase V0a1 at the site of its synthesis as the polypeptide chain emerges from the ribosome at the translocon or early trafficking rather than the site of its final destination at the lysosome.

**Fig. 7:**
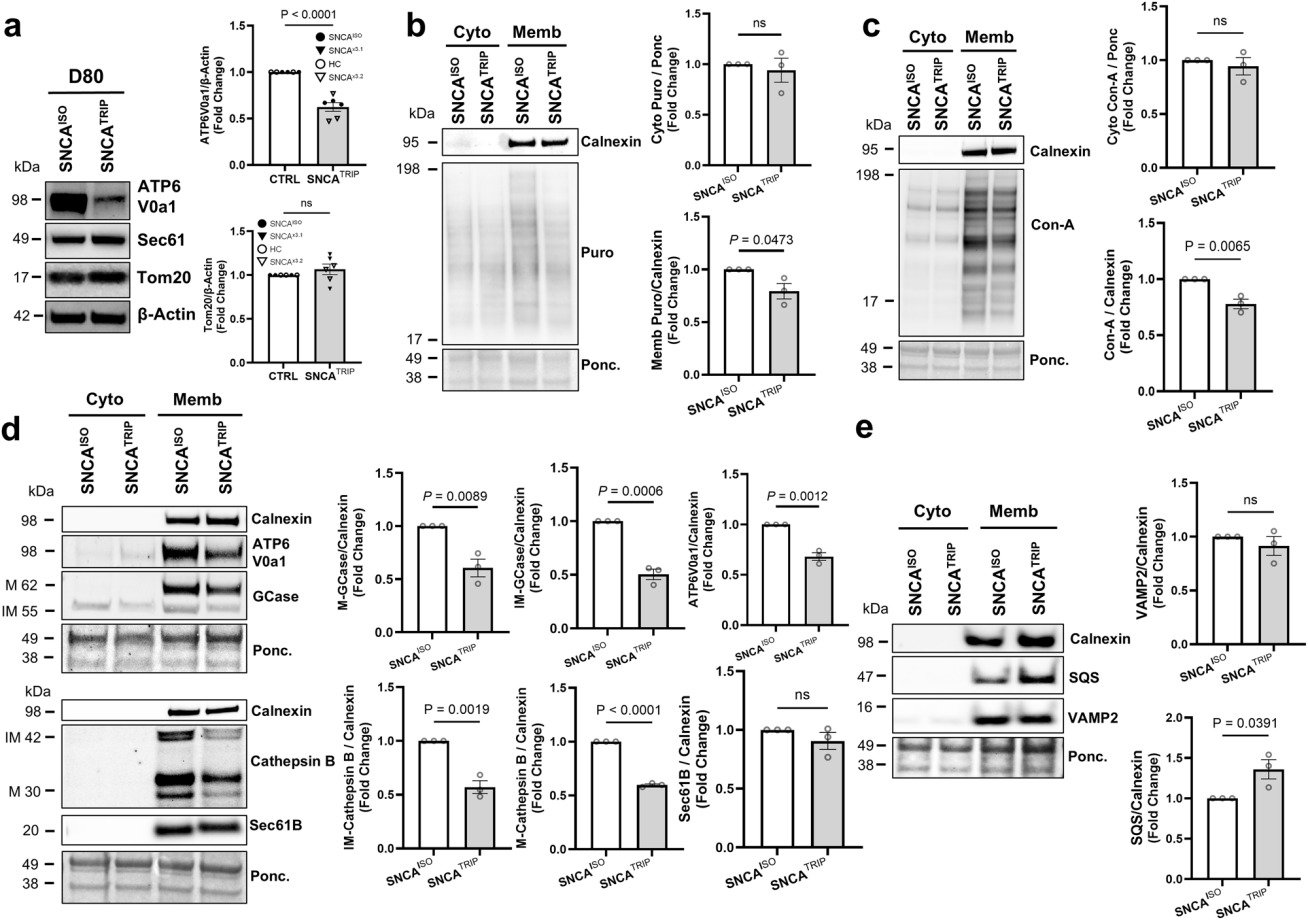
Defective co-translational translocation reduces organelle-associated protein levels. **a** Representative immunoblots and quantification, showing a relative reduction in ATP6V0a1 levels in *SNCA*^*TRIP*^ compared to *SNCA*^*ISO*^ control hiPSC-DANs at D80. Tom20, which is not a translocon substrate, was unchanged across conditions. ATP6V0a1 (CTRL|*SNCA*^*TRIP*^: Two-sided Unpaired *t*-test—t(4) = 2.709, *p* = 0.0220) and Tom20 (CTRL|*SNCA*^*TRIP*^: Two-sided Unpaired *t*-test—t(4) = 0.7609, *p* = 0.4643), *n* = 6. **b** Representative immunoblots and quantification showing decreased puromycin-labelled proteins in the membrane (Memb) fraction but not the cytosolic (Cyto) fraction of D80 *SNCA*^*TRIP*^ compared to *SNCA*^*ISO*^ hiPSC-DANs. *SNCA*^*ISO*^|*SNCA*^*TRIP*^: Two-sided Unpaired *t*-test—t(4) = 2.83, *p* = 0.0473, *n* = 6. **c** Representative images and quantification showing decreased Concanavalin-A (Con-A) labelled glycosylated proteins in the Memb fraction but not the Cyto fraction of D80 *SNCA*^*TRIP*^ compared to *SNCA*^*ISO*^ hiPSC-DANs. Memb *SNCA*^*ISO*^|Memb *SNCA*^*TRIP*^: Two-sided Unpaired *t*-test—t (4) = 5.198, *p* = 0.0065. D80 Cyto *SNCA*^*ISO*^|Cyto *SNCA*^*TRIP*^: Two-sided Unpaired *t*-test—t(4) = 0.7002, *p* = 0.5224, *n* = 3. **d** Representative immunoblots and quantification showing decreased levels of PD risk modifiers in the membrane/organelle compartment in *SNCA*^*TRIP*^ compared to *SNCA*^*ISO*^ control hiPSC-DANs including ATP6V0a1 (*SNCA*^*ISO*^|*SNCA*^*TRIP*^: Two-sided Unpaired *t*-test—t(4) = 8.30, *p* = 0.0012), mature (M) GCase (*SNCA*^*ISO*^|*SNCA*^*TRIP*^: Two-sided Unpaired *t*-test—t(4) = 4.76, *p* = 0.0089), immature (IM) GCase (*SNCA*^*ISO*^|*SNCA*^*TRIP*^: Two-sided Unpaired *t*-test—t(4) = 10.01, *p* = 0.0006), IM Cathepsin B (*SNCA*^*ISO*^|*SNCA*^*TRIP*^: Two-sided Unpaired *t*-test—t(4) = 7.222, *p* = 0.0019) and M Cathepsin B (*SNCA*^*ISO*^|*SNCA*^*TRIP*^: Two-sided Unpaired *t*-test—t(4) = 35.59, *p* < 0.0001). Protein levels of the GET substrates Sec61B (*SNCA*^*ISO*^ | *SNCA*^*TRIP*^: Two-sided Unpaired t-test—t(4) = 1.304, *p* = 0.2622) and **e** VAMP2 (*SNCA*^*ISO*^|*SNCA*^*TRIP*^: Two-sided Unpaired *t*-test—t(4) = 0.9832, *p* = 0.3812) were similar in *SNCA*^*TRIP*^ compared to *SNCA*^*ISO*^ control hiPSC-DANs. Protein levels of the EMC substrate SQS (*SNCA*^*ISO*^|*SNCA*^*TRIP*^: Two-sided Unpaired *t*-test—t (4) = 3.022, *p* = 0.0391) increased in *SNCA*^*TRIP*^ compared to *SNCA*^*ISO*^ control hiPSC-DANs, *n* = 3. Each dot corresponds to one clone differentiated once and data are mean ± SEM from at least *n* = 3 differentiations per clone. Source data are provided as a Source Data file.

To further investigate the proposed mechanism, we quantified the translation of newly synthesised proteins that translocate into the ER using Sec61A, EMC or GET dependent pathways. To this end, hiPSC-DANs from *SNCA*^*TRIP*^ or their isogenic controls were pulsed with puromycin (10 μg/ml) for 10 min, followed by PLA using antibodies against puromycin and relevant substrates. We found that the translation of Sec61A substrates v-ATPase V0a1 and GBA was reduced (Fig. 6d, e) whereas the translation of VAMP2 and Sec61B (GET substrates) or SQS (EMC substrate) was unaffected (Fig. 6f, g). Accordingly, we found a relative reduction in v-ATPase V0a1 levels by immunoblotting in *SNCA*^*TRIP*^ hiPSC-DANs compared to control neurons that was most prominent at D80 (Fig. 7a and Supplementary Fig. 11a, b). In contrast, the levels of the mitochondrial protein Tom20 which is not a translocon substrate, were unaffected (Fig. 7a).

To assess the global impact of the identified ER-associated translational defect, we treated D80 hiPSC-DANs with puromycin followed by neuronal fractionation with a view to quantifying the abundance of labelled proteins in the cytosol vs membranous compartments. We found reduced levels of puromycin-labelled proteins in *SNCA*^*TRIP*^ hiPSC-DANs compared to the isogenic control DANs only in the membrane fraction (Fig. 7b), suggesting a widespread defect in ER-associated translation of membrane/organelle-associated proteins. To corroborate this conclusion with an alternative readout of global ER-associated protein processing, we used Concanavalin A (Con A), a lectin that binds to α-D-mannosyl and α-D-glucosyl residues on glycoproteins. This analysis also revealed lower protein glycosylation in the membrane protein fraction isolated from *SNCA*^*TRIP*^ hiPSC-DANs (Fig. 7c). In these experiments, we further confirmed the reduction in v-ATPase V0a1 subunit as well as other translocon substrates which are genetic modifiers such as GCase encoded by the PD risk gene *GBA1* and Cathepsin B, encoded by the PD gene *CTSB* (Fig. 7d). Protein levels of the GET or EMC substrates Sec61B, SQS or VAMP2 were not reduced (Fig. 7d, e). The mRNA levels of relevant substrates were unchanged in our RNA-seq analysis (Supplementary Fig. 11c). Thus, defective ER-associated translation impairs the synthesis and/or trafficking of the v-ATPase V0a1 as well as other translocon substrates including the lysosomal proteins GCase and Cathepsin B. To further verify these findings, we treated hiPSC-DANs overnight with the Sec61A inhibitor Eeyarestatin (20 μM). Pharmacological inhibition of the translocon with ESI revealed a reduction in the levels of the ATPase V0a1 subunit (Supplementary Fig. 11d, e) and mature Cathepsin B (Supplementary Fig. 11f, g) most prominently in *SNCA*^*TRIP*^ hiPSC-DANs with accumulation of immature Cathepsin B, mainly in *SNCA*^*ISO*^ hiPSC-DANs corresponding to non-translocated species as would be expected from a pre-existing defect in *SNCA*^*TRIP*^, without an effect on Sec61B or VAMP2 (Supplementary Fig. 11d–g).

### Defective ER-associated translocation impairs lysosome function and increases αSyn release in extracellular vesicles

**Fig. 8:**
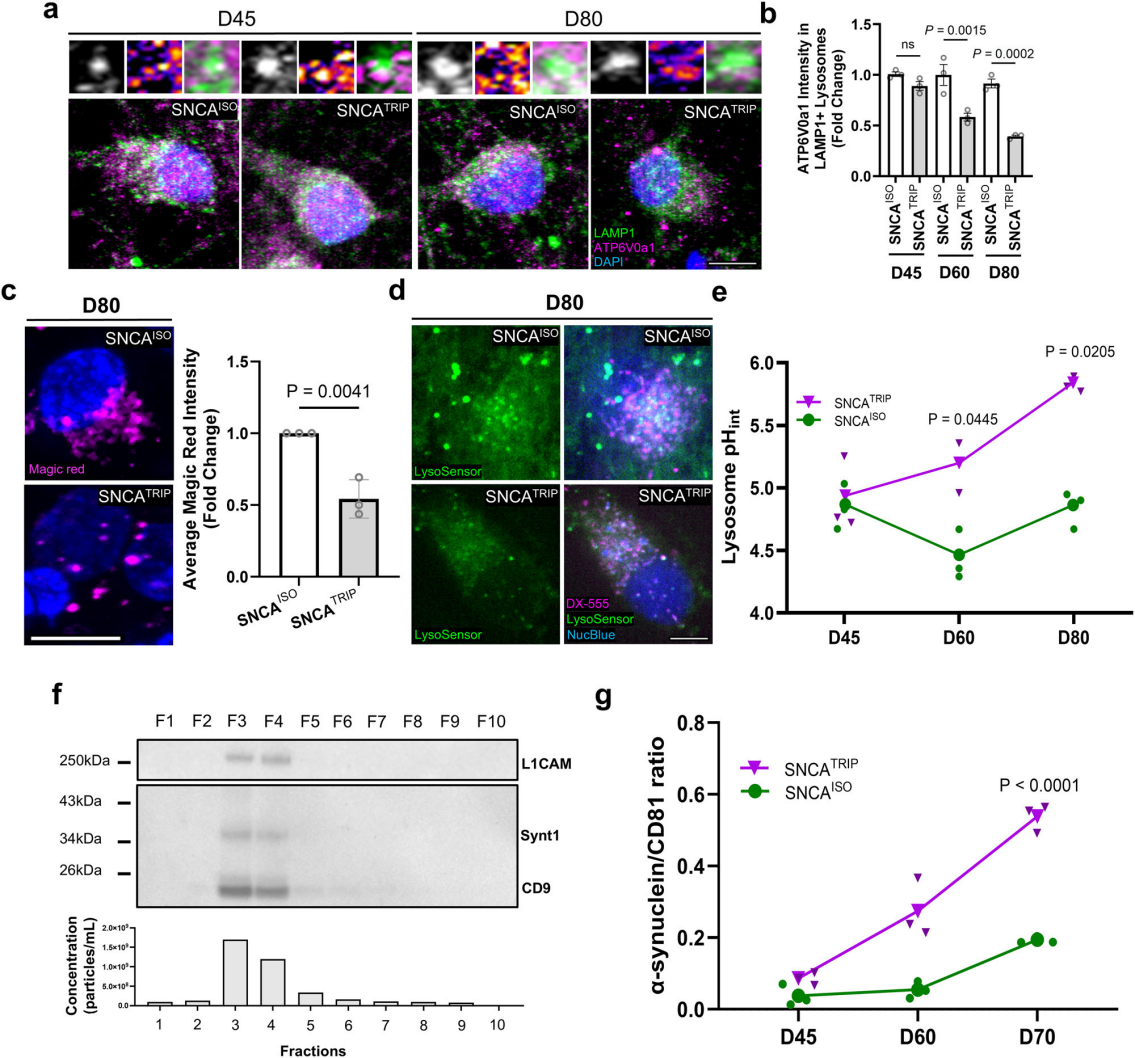
Defective ER-associated translocation impairs lysosome acidification and increases egress of EV-associated αSyn. **a** Representative images and quantification in (**b**), showing decreased intensity of v-ATPase subunit ATP6V0a1 in LAMP1-positive lysosomes over time in *SNCA*^*TRIP*^ hiPSC-DANs compared to *SNCA*^*ISO*^ controls, most prominently at D80. Two-way ANOVA - *F*(5,10) = 28.23, *p* < 0.0001; *SNCA*^*ISO*^ D80|*SNCA*^*TRIP*^-D80: t(6) = 7.81, *p* = 0.0002, *n* = 3. **c** Representative images and quantification of Cathepsin B activity showing a decrease in Magic Red fluorescence intensity in *SNCA*^*TRIP*^ hiPSC-DANs compared to *SNCA*^*ISO*^ controls at Day 80. *SNCA*^*ISO*^|*SNCA*^*TRIP*^, Two-sided Unpaired *t*-test—t(4) = 5.912, *p* = 0.0041, *n* = 3. **d** Representative Day 80 images and quantification in (**e**), showing progressive failure of lysosomal acidification over time in *SNCA*^*TRIP*^ hiPSC-DANs compared to isogenic control hiPSC-DANs. Two-way ANOVA—*F* (2,9) = 11.36, *p* < 0.0001; D60 SNCA^ISO^|*SNCA*^*TRI*P^: *p* = 0.0455, D80 SNCA^ISO^|*SNCA*^*TRIP*^-FIB: *p* = 0.0205, *n* = 3. **f** Representative immunoblot demonstrating extracellular vesicles are eluted only in fractions 3 and 4 (upper) and corresponding nanoparticle tracking analysis measurements confirming the highest concentration of particles only in fractions 3 and 4 (lower). **g** Quantification of αSyn/CD81 ratio in EV-enriched fractions from conditioned media showing progressive increase in αSyn release over time in *SNCA*^*TRIP*^ hiPSC-DANs compared to isogenic control hiPSC-DANs. Two-way ANOVA—*F* (1,4) = 88.84, *p* = 0.0007; D70 SNCA^ISO^|*SNCA*^*TRI*P^: *p* < 0.0001, *n* = 3. Each dot corresponds to one clone differentiated once and data are mean ± SEM from at least *n* = 3 differentiations per clone. Source data are provided as a Source Data file.

To investigate the functional consequences of reduced v-ATPase V0a1 subunit and Cathepsin B synthesis, we first tested and confirmed a progressive decline in the co-localisation of v-ATPase V0a1 subunit with Lamp1 positive lysosomes in *SNCA*^*TRIP*^ hiPSC-DANs compared to isogenic control DANs (Fig. 8a, b), in the absence of any change in lysosome size or number (Supplementary Fig. 12a, b). We next assessed lysosomal Cathepsin B activity using the Magic Red® assay, a fluorogenic substrate that becomes fluorescent upon cleavage by active Cathepsin B within lysosomes. Consistent with impaired Cathepsin B synthesis, *SNCA*^*TRIP*^ hiPSC-DANs exhibited reduced Magic Red fluorescence intensity compared to isogenic control DANs at D80 (Fig. 8c). We then measured lysosomal acidification using a ratiometric live-cell lysosome pH assay in *SNCA*^*ISO*^ or *SNCA*^*TRIP*^ hiPSC-DANs at D45, D60 and D80 (Supplementary Fig. 12 c). Lysosomal pH in healthy hiPSC-DANs was maintained within the expected acidic range (pH 4.4–4.8) at all tested time points (Fig. 8d and quantified in Fig. 8e). In contrast, there was a progressive failure of lysosomal acidification in *SNCA*^*TRIP*^ hiPSC-DANs with higher (i.e. more basic) pH detected at D60 (average pH = 5.17) and D80 (average pH = 5.82), (Fig. 8d and quantified in 8e).

We then assessed whether the release of αSyn in EVs is affected by impaired lysosomal acidification as also predicted by our pseudotime trajectory (Fig. 3g). To this end, we collected conditioned media from *SNCA*^*TRIP*^ or isogenic control hiPSC-DANs at D45, D60 and D70 that were fractionated using size exclusion to separate neuronal EVs in F3 and F4 from free secreted proteins in later fractions (Fig. 8f) as we have done previously^19^. αSyn in EVs as quantified by the αSyn/CD81 ratio was progressively increased in *SNCA*^*TRIP*^ hiPSC-DANs with time in culture compared to isogenic control hiPSC-DANs (Fig. 8g). Pharmacological inhibition of v-ATPase with bafilomycin (200 nM) in dopaminergic M17D neuroblastoma cells stably overexpressing αSyn phenocopied these defects by increasing the release of αSyn in EVs (Supplementary Fig. 13). Thus, defective synthesis/trafficking of the v-ATPase V0a1 subunit and other translocon substrates impairs the acidification and function of lysosomes and potentially other organelles whose function is dependent on the v-ATPase pump, diverting αSyn in EVs for release outside the cell.

### Defective ER-associated translocation can be rescued with *SNCA* gene silencing or pharmacological activation of the proteasome

**Fig. 9:**
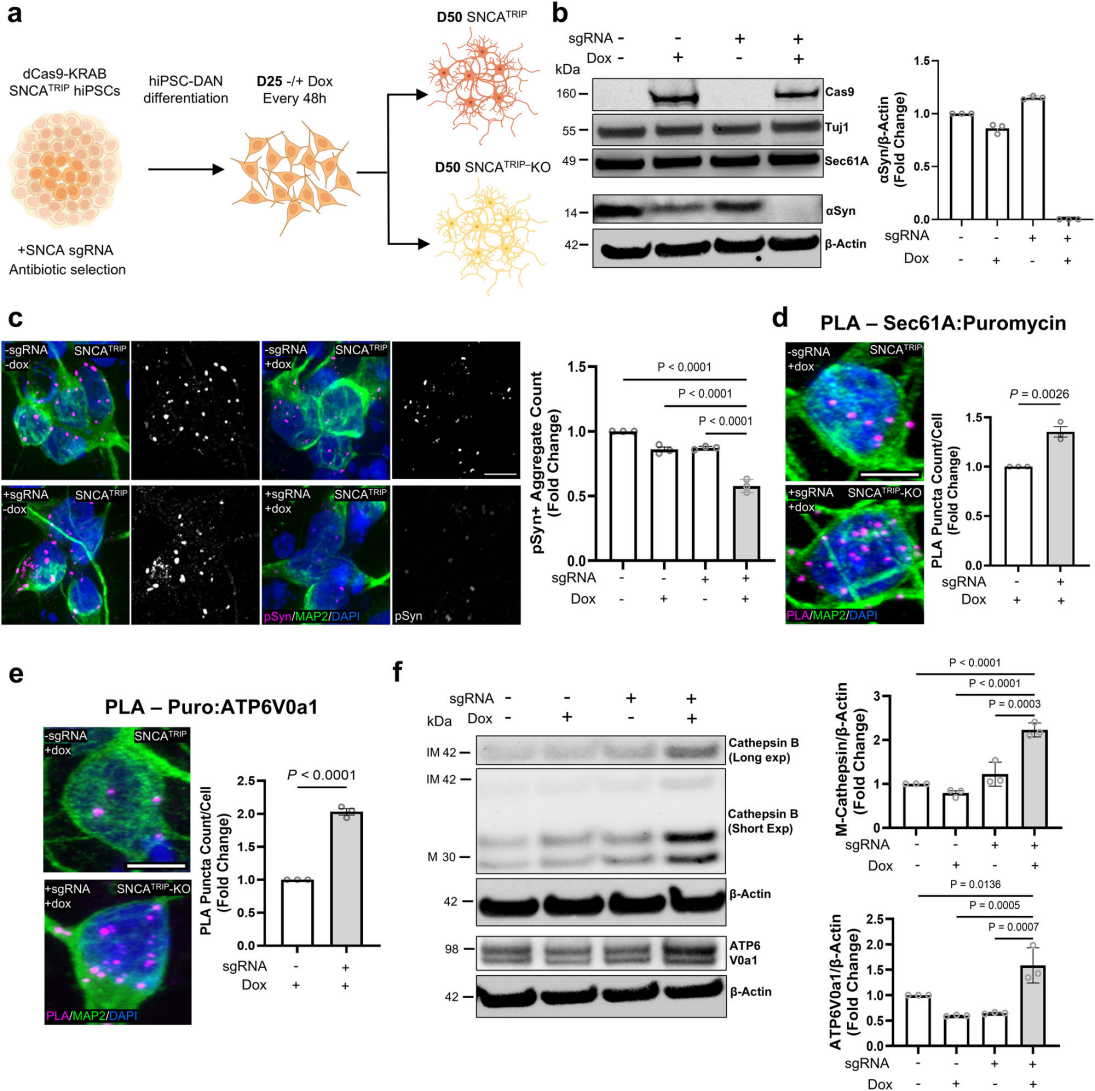
*SNCA* gene silencing improves co-translational translocation in *SNCA*^*TRIP*^ hiPSC-DANs. **a** Schematic detailing the doxycycline-inducible (Dox) CRISPR-dCas9 system and timeline for αSyn knockout (KO) in *SNCA*^*TRIP*^ hiPSC-DANs. Image Created with BioRender https://BioRender.com/699uz0t. **b** Representative immunoblots and quantification showing knockout of αSyn levels in *SNCA*^*TRIP*^ hiPSC-DANs treated with Dox compared to controls; Sec61A levels remain unchanged. **c** Representative iSIM images and quantification showing a decrease in aggregate count in *SNCA*^*TRIP*^ +sgRNA +Dox hiPSC-DANs compared to controls. Ordinary one-way ANOVA—F(3,8) = 99.60, *p* < 0.0001; *SNCA*^*TRIP*^ -sgRNA -Dox |*SNCA*^*TRIP*^ +sgRNA +Dox: *p* < 0.0001, *SNCA*^*TRIP*^ -sgRNA +Dox |*SNCA*^*TRIP*^ +sgRNA +Dox: *p* < 0.0001, *SNCA*^*TRIP*^ +sgRNA -Dox |*SNCA*^*TRIP*^ +sgRNA +Dox: *p* < 0.0001, *n* = 3. Scale bar = 5μm. **d** Representative images and quantification of PLA between Sec61A and puromycin-labelled chains, showing increased ER translation in *SNCA*^*TRIP*^ +Dox hiPSC-DANs compared to *SNCA*^*TRIP*^ -Dox hiPSC-DANs; *SNCA*^*TRIP*^ -Dox*|SNCA*^*TRIP*^ +Dox: Unpaired *t*-test—t (4) = 6.69, *p* = 0.0026, *n* = 3. Scale bar = 5 μm. **e** Representative images and quantification of PLA between puromycin-labelled nascent chains and ATP6V0a1, demonstrating increased translation; *SNCA*^*TRIP*^ -sgRNA +Dox|*SNCA*^*TRIP*^ +sgRNA +Dox: Two-sided Unpaired *t*-test—t (4) = 21.04, *p* < 0.0001, *n* = 3. Scale bar = 5 μm. **f** Representative immunoblots and quantification showing increased protein levels of Sec61A substrates upon αSyn knockout including mature (M) Cathepsin B (ANOVA—F(3,8) = 99.60, *p* < 0.0001; *SNCA*^*TRIP*^ -sgRNA -Dox |*SNCA*^*TRIP*^ +sgRNA +Dox: *p* < 0.0001, *SNCA*^*TRIP*^ -sgRNA +Dox |*SNCA*^*TRIP*^ +sgRNA +Dox: *p* < 0.0001, *SNCA*^*TRIP*^ +sgRNA -Dox |*SNCA*^*TRIP*^ +sgRNA +Dox: *p* < 0.0001) and ATP6V0a1 (ANOVA—F(3,8) = 20.88, *p* < 0.0001; *SNCA*^*TRIP*^ -sgRNA -Dox |*SNCA*^*TRIP*^ +sgRNA +Dox: *p* = 0.0136, *SNCA*^*TRIP*^ -sgRNA +Dox |*SNCA*^*TRIP*^ +sgRNA +Dox: *p* = 0.0005, *SNCA*^*TRIP*^ +sgRNA -Dox |*SNCA*^*TRIP*^ +sgRNA +Dox: *p* = 0.0007), *n* = 3. Each dot corresponds to one clone differentiated once and data are mean ± SEM from at least *n* = 3 differentiations per clone. Source data are provided as a Source Data file.

It is possible that early pathological forms of αSyn and their associated subcellular toxicity can be reversed by intervention. To address this issue, we generated an inducible dCas9 hiPSC line under the control of doxycycline in the *SNCA*^*TRIP*^ background (Supplementary Figs. 14–15). HiPSCs were transduced with a lentivirus expressing sgRNA targeting *SNCA* and differentiated into DANs (Fig. 9a). Doxycycline was added from D25 onwards, achieving almost a complete knockout of αSyn levels by D50 (Fig. 9b), and consequently a reduction in pSyn aggregate count by D60 as confirmed by iSIM (Fig. 9c). HiPSC-DANs were then pulsed with puromycin and processed for Sec61A/puromycin PLA as previously. We found that the PLA signal increased when αSyn levels and aggregation were reduced, signifying improved interaction between puromycin-labelled elongating nascent chains with Sec61A (Fig. 9d). We further corroborated this finding by measuring specifically the puromycylation of the Sec61A substrate v-ATPase V0a1 using PLA. These experiments confirmed that reduction in αSyn levels in *SNCA*^*TRIP*^ hiPSC-DANs improved the translation of v-ATPase V0a1 (Fig. 9e). Immunoblotting for translocon substrates in the membrane fraction of neuronal lysates also showed that reduction of αSyn increased the levels of v-ATPase V0a1 and Cathepsin B compared to controls (Fig. 9f).

**Fig. 10:**
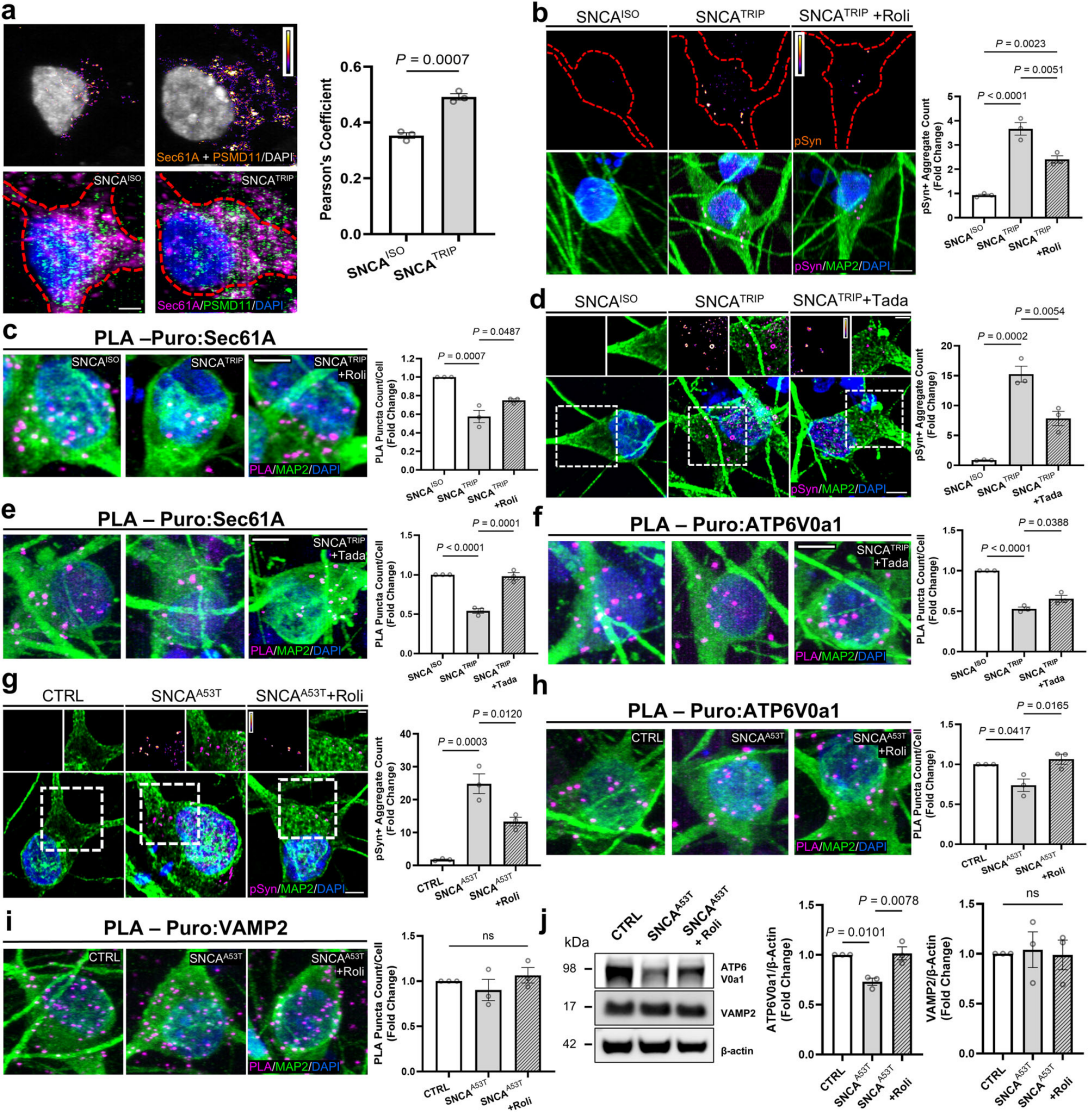
Proteasome activation rescues the ER-defect in *SNCA*^*TRIP*^ and *SNCA*^*A53T*^ hiPSC-DANs. **a** Representative images of combined Sec61A and PSMD11 (upper) staining and merged overlays (lower) with quantification (right) showing increased colocalisation between the 26S proteasome subunit PSMD11 and Sec61A; Data are presented as mean ± SEM, Scale bar = 1 μm. **b** Representative images and quantification showing reduced pSyn-positive puncta in *SNCA*^*TRIP*^ hiPSC-DANs treated with rolipram compared to untreated *SNCA*^*TRIP*^ hiPSC-DANs. Ordinary one-way ANOVA—*F*(2,6) = 62.58, *p* < 0.0001; *SNCA*^*TRIP*^|*SNCA*^*TRIP*^ +Roli: *p* = 0.0051, *n* = 3. Scale bar = 5 μm. **c** Representative images and quantification showing increased intensity of puromycin-labelled proteins in *SNCA*^*TRIP*^ hiPSC-DANs treated with rolipram compared to untreated *SNCA*^*TRIP*^ hiPSC-DANs. Ordinary one-way ANOVA—*F*(2,6) = 54.10, *p* = 0.0001; *SNCA*^*TRIP*^|*SNCA*^*TRIP*^ +Roli: *p* = 0.0052, *n* = 3. Scale bar = 5 μm. **d** Representative images and quantification showing reduced pSyn-positive puncta in *SNCA*^*TRIP*^ hiPSC-DANs treated with tadalafil compared to untreated *SNCA*^*TRIP*^ hiPSC-DANs. Ordinary one-way ANOVA—*F*(2,6) = 48.85, *p* = 0.0002; *SNCA*^*TRIP*^|*SNCA*^*TRIP*^ +Tada: *p* = 0.0054, *n* = 3. Scale bar = 5 μm. **e** Representative images and quantification of PLA between Sec61A and puromycin-labelled proteins, demonstrating increased ER-associated translation in tadalafil treated *SNCA*^*TRIP*^ hiPSC-DANs compared to untreated *SNCA*^*TRIP*^ hiPSC-DANs. Ordinary one-way ANOVA—*F*(2,6) = 73.31, *p* < 0.0001; *SNCA*^*TRIP*^|*SNCA*^*TRIP*^ +Tadal: *p* = 0.0001, *n* = 3. Scale bar = 5 μm. **f** Representative images and quantification showing reduced puromycin-labelled ATP6V0a1 in *SNCA*^*TRIP*^ hiPSC-DANs compared to *SNCA*^*ISO*^ and rescue with tadalafil. Ordinary one-way ANOVA—*F*(2,6) = 54.10, *p* = 0.0001; *SNCA*^*TRIP*^|*SNCA*^*TRIP*^ +Tadal: *p* = 0.0052, *n* = 3. Scale bar = 5 μm. **g** Representative images and quantification showing reduced pSyn-positive puncta in *SNCA*^*A53T*^ hiPSC-DANs treated with rolipram compared to untreated *SNCA*^*A53T*^ hiPSC-DANs. Ordinary one-way ANOVA—*F*(2,6) = 37.16, *p* = 0.0004; *SNCA*^*A53T*^|*SNCA*^*A53T*^ +Roli: *p* = 0.0120, *n* = 3. Scale bar = 5 μm. **h** Representative images and quantification showing reduced puromycin-labelled ATP6V0a1 in *SNCA*^*A53T*^ hiPSC-DANs compared to healthy control DANs and rescue with rolipram. Ordinary one-way ANOVA—*F*(2,6) = 9.03, *p* = 0.0155; *SNCA*^*A53T*^|*SNCA*^*A53T*^ +Roli: *p* = 0.0165, *n* = 3. Scale bar = 5 μm. **i** Representative images and quantification showing no effect on puromycin-labelled VAMP2. Ordinary one-way ANOVA—*F*(2,6) = 0.9613, *p* = 0.4344, *n* = 3. Scale bar = 5 μm. **j** Representative immunoblots and quantification showing reduced ATP6V0a1 levels in *SNCA*^*A53T*^ hiPSC-DANs compared to control DANs and rescue with rolipram. Ordinary one-way ANOVA—*F*(2,6) = 14.09, *p* = 0.0054; *SNCA*^*A53T*^|*SNCA*^*A53T*^+Roli: *p* = 0.0078, *n* = 3. Each dot corresponds to one clone differentiated once and data are mean ± SEM from at least *n* = 3 differentiations per clone. Source data are provided as a Source Data file.

To assess whether the ER-defect can be rescued with a pharmacological agent, we treated *SNCA*^*TRIP*^ hiPSC-DANs (wildtype without the dCas9 system) with rolipram which was previously shown to activate the proteasome^20^. Proteasomal subunits were detected in our proteomic analysis at later timepoints (see Fig. 2b), suggesting that proteasomes are recruited to this site of subcellular toxicity. We first tested and confirmed that proteasome colocalisation with Sec61A is increased in *SNCA*^*TRIP*^ hiPSC-DANs compared to controls using an antibody against the 26S subunit PSMD11 that was also detected in our proteomic analysis (Fig. 10a). We then treated the neuronal cultures with rolipram (25 μM) from D50, a timepoint with established nanoscale aggregates (see Fig. 3a), until D80. We found that rolipram treatment reduced the number of pSyn puncta as assessed Airyscan confocal microscopy (Fig. 10b). To assess nascent polypeptide chain synthesis at the translocon, we then performed Sec61A/puromycin PLA, which revealed increased signal intensity in rolipram treated hiPSC-DANs (Fig. 10c) as observed with *SNCA* knockout. Rolipram is an inhibitor of phosphodiesterase 4 (PDE4), causing an increase in cAMP levels and activation of PKA, which phosphorylates numerous targets beyond proteasome subunits. We therefore also investigated an alternative mechanism of proteasome activation by treating *SNCA*^*TRIP*^ hiPSC-DANs with tadalafil, which acts by increasing cGMP levels via inhibition of PDE5^21^. We found that tadalafil (2 μM) also reduced pSyn levels (Fig. 10d) and increased translation at the translocon based on either overall translation proximal to Sec61A (Fig. 10e) or v-ATPase V0a1 (Fig. 10f) puromycin-labelled polypeptide chains as measured by PLA.

Lastly, to assess the identified phenotype and effect of proteasome activation in a separate model of αSyn aggregation, we generated hiPSC-DANs from patients with the A53T mutation (*SNCA*^*A53T*^). In this model we detected de novo formation of pSyn+ aggregates by D80 (Fig. 10g). Treatment of the *SNCA*^*A53T*^ hiPSC-DAN cultures with rolipram (25 μM) from D50 to D80 reduced pSyn+ aggregates (Fig. 10g) and mitigated the ER-associated translation defect as measured by PLA of puromycylated v-ATPase V0a1 (Fig. 10h). VAMP2 translation used as control was unaffected by either the A53T mutation or rolipram treatment (Fig. 10i). Immunoblotting of neuronal lysates corroborated the puromycylation findings, showing a reduction in ATPase V0a1 levels in *SNCA*^*A53T*^ hiPSC-DAN that increased after treatment with rolipram (Fig. 10j). Thus, reducing pathological αSyn by knockout or proteasomal activation can partially mitigate the ER-translational defect.

## Discussion

Despite extensive investigations in animal and hiPSC models^1,22^, the subcellular localisation of early-stage pathology and the conformation of αSyn responsible for its cytotoxicity remain unresolved. The development of seeded-aggregation models has delineated cellular phenotypes in neurons with macroscopic aggregates that resemble Lewy bodies^3,23,24^ and identified damaged organelles induced by the entry of exogenously generated fibrils or oligomers, such as lysosomes^25,26^ and mitochondria^27^. However, these changes are limited by the temporal resolution of pathology and may not reflect the initial subcellular site of damage caused by de novo αSyn misfolding events. To bypass this limitation, we employed orthogonal multi-omic data-driven approaches to spatially and temporally resolve the consequences of pathological αSyn accumulation secondary to over-expression, *SNCA* gene triplication, *SNCA A53T* mutation or seeded aggregation.

A major finding of our proteomic analysis is that nanoscale foci of αSyn that are not detectable by conventional confocal microscopy were associated with more aberrant protein interactions whereas larger assemblies were comparatively inert. This finding supports the long-held notion that deposition of fibrillar assemblies into Lewy bodies may represent an attempt by the cell to isolate toxic intermediate conformations^28^. It is noteworthy that in human post-mortem brain tissue smaller scale αSyn aggregates are widespread when using more sensitive techniques compared to Lewy bodies and neurites^29,30^ and could represent the neuroanatomical equivalent of the foci we detected in hiPSC-derived neurons with iSIM. Although the precise structure of the most toxic conformer is unknown, our modelling suggests that even partial unfolding of the α-helix of αSyn may be sufficient to promote aberrant interactions as modelled here for the translocon. It was recently shown that a region within the helically folded N-terminus of αSyn is pivotal for its structural transition into fibrils and the G51D mutation induces a conformational fluctuation that could increase unfolding events in this region^31^. This suggests a biophysical basis for the generation of conformational intermediates that may be damaging as proposed by our model. Interestingly, antibodies against the N-terminus (aa 34–57) identified high burden of proteinase K sensitive (i.e. non fibrillar) αSyn pathology in postmortem PD brains with distinct morphologies, including a fine punctate pattern^8^ similar to the one we detected in hiPSC DANs with *SNCA* triplication or A53T mutation. We propose that unfolding of the helical structure and exposure of this N-terminal region, which is normally buried either in the membrane or the core of the fibril^32^ is a pathogenic event, in our hands by impairing translocon function. In this respect, the detection of nanoscale aggregates in our model most likely signifies the formation of pathogenic intermediates in the cell, rather than direct toxicity from these puncta. Although pSyn is also detected under physiological conditions and regulates membrane binding^33^, our observations were made in human DANs with pathological αSyn due to *SNCA* gene triplication or A53T mutation and supported by the detection of misfolded proteoforms under native conditions (i.e. dot blot). We previously found that Lys 45, 58, 60 which are adjacent to the aforementioned region are modified by ubiquitination^34^. This offers one mechanism by which partial unfolding of the helical N-terminus exposes these lysine residues, selectively and rapidly targeting potentially toxic forms of αSyn for destruction.

Another important finding of our work is the identification of co-translational translocation of nascent chains into the ER as the subcellular localisation of early-stage toxicity. αSyn aggregates were previously reported by electron microscopy in association with the rough ER in transgenic mice^35^. Our data suggest that early stage αSyn conformational intermediates may block the translocation of substrates across Sec61A, impairing their synthesis and/or trafficking, before macroscale aggregates form. This focus of pathology through impaired translocation of nascent chains into the ER offers a unifying mechanism for the previously described diverse PD cellular phenotypes and effectors in organelle homoeostasis. For example, we showed that the levels of translocon substrates encoded by the PD-associated genes *ATP6V01A*, *GBA1* and *CTSB* are reduced under conditions of pathological αSyn induced ER translational defects: *ATP6V0A1* encodes a membrane-associated subunit of the v-ATPase which is critical for organelle acidification, a prerequisite for optimal lysosomal hydrolase activation and function, vesicular trafficking, endocytosis and autophagy that are all implicated in PD pathogenesis^36,37^. Progressive decline in GCase protein levels and activity without changes in *GBA1* mRNA were previously detected in PD brain areas with early αSyn accumulation before substantial Lewy body formation^38,39^. Cathepsin B is a critical protease for the degradation of αSyn aggregates^40^ and a GWAS candidate risk gene^17^. Thus, an αSyn-induced co-translational translocation block at the ER could phenocopy lysosomal and other organelle dyshomeostasis that was reported with monogenic or polygenic PD risk modifiers or ageing. These intracellular lysosomal defects and our finding of related GO terms (e.g. organelle subcompartment, extracellular vesicles) in the pathological pseudotime trajectory, confirmed experimentally in hiPSC-DANs, also explain why αSyn is diverted to and released in neuronally-derived EVs that are detectable in blood^41,42^.

Unlike previous studies in hiPSC neurons^43^, our finding of an ER nascent chain translocation defect rather than an ER protein accumulation is consistent with the fact that αSyn-induced ER stress is not associated, at least in the early stage of pathology, with activation of the UPR as shown by us herein and others in αSyn hiPSC or animal models^43,44^. Instead, we observed increased UFMylation which is known to act as a posttranslational modification for the removal and degradation of stalled ribosomes and associated nascent chains^13^. Our spatio-temporal analysis does not contradict previous mechanisms such as defects in ER-to-Golgi transport^45^ or ER-associated calcium regulation^46^ but rather suggest that an ER translocation defect is detectable in hiPSC-derived DANs very early in the pathogenic cascade in the absence of inclusion body formation.

Despite offering a snapshot of protein interactions occurring in the living cell, the APEX tag may impede or modify the mode of interaction of αSyn with certain subcellular compartments. However, our orthogonal validation with transcriptomics and mechanistic investigations make it unlikely that the identified effectors are an artefact of the in vitro system. The interactome of other proteins such as mutant tau-APEX in hiPSC-derived neurons identified primarily mitochondrial protein interactions as mediators of pathology^47^, further suggesting that the ER-associated translocation defect we observed herein may be specific to αSyn. Previous studies using αSyn-APEX in primary cortical neurons identified ribosomal subunits and endocytic trafficking components^48^. However, subsequent studies by the same group suggested a physiological function of αSyn in cytosolic translation via interaction with Edc4 and related P-body complexes^49^. In our interactome analysis, Edc4 and related proteins were not the most prominent interactors. Although our data do not necessarily exclude a physiological or pathological role in translation via P-body or other interactions, they do unify defects in trafficking via a pathological effect on ER-associated rather than cytosolic translation. In agreement with this conclusion, differential gene expression analysis from the ROSMAP data identified ‘protein localisation to the ER’ as a top GO term being negatively affected by Lewy body pathology^49^ as seen in our transcriptomic analysis of hiPSC-derived neurons.

We showed that reducing αSyn levels in *SNCA*^*TRIP*^ DANs by dCas9-mediated *SNCA* gene silencing reversed the ER translocation defect and this was phenocopied by proteasomal activation with rolipram or tadalafil. Rolipram is a selective phosphodiesterase-4 inhibitor that raises intracellular cAMP, leading to proteasome activation by PKA mediated phosphorylation^20^ whereas tadalafil is a selective phosphodiesterase-5 inhibitor that activates the proteasome by raising intracellular cGMP^21^. Although we cannot exclude an alternative or additional pharmacological mechanism of action, the use of two different drugs with a convergent target supports the role of proteasomal clearance in our model, as previously shown for these compounds in other disease-causing proteins such as mutant huntingtin and tau^21^. Our extensive previous studies showed that under non-aggregation conditions, αSyn is degraded primarily by the lysosome^34^. However, given the decrease in lysosomal acidification and Cathepsin B activity reported herein, it is not surprising that proteasomes are recruited to degrade misfolded αSyn under pathological conditions. It is likely that proteasomal activation also degrades misfolded nascent chains or stalled ribosome subunits following αSyn-induced Sec61A blockade. Such function would be consistent with our detection of increased UFMylation, which serves as a signal for proteasomal degradation under such conditions^13^. Thus, because of lysosomal impairment in early stages of the disease, activation of the proteasome with repurposed drugs such as rolipram or tadalafil should be considered as a viable therapeutic strategy.

There are limitations to our study: The *SNCA* triplication is rare for multiple patient lines to be analysed but the use of an isogenic pair throughout this study, including independently derived clones and healthy control lines as well as the reversal of key phenotypes with CRISPRi in the same genetic background partly mitigate for this limitation. Although we have further validated the identified mechanism in hiPSC lines from PD patients with the A53T mutation, generalisation to sporadic synucleinopathies and different neuronal subtypes requires further investigation. In this respect, our post-mortem data demonstrating an interaction between pathological αSyn and Sec61A in the human substantia nigra irrespective of sex support relevance to sporadic PD. Currently, there are no specific tools to trace early misfolded αSyn intermediates in cells. Therefore, the precise high-resolution structure of the αSyn proteoform that blocks Sec61A as well as a structural confirmation of the proposed interaction remain a future challenge.

Collectively, our study of αSyn pathology in hiPSC-derived DANs identified αSyn-induced blockade of co-translational translocation at the ER as an early pathogenic mechanism that unifies aggregation with dysregulation of diverse organelle-associated proteins that are both translocon substrates and genetic modifiers. Our results also offer a mechanistic rationale for proteasomal activation with repurposed drugs already used in neurological practice as a therapeutic strategy to mitigate this defect.

## Methods

### Ethical approval

Human post-mortem tissue and associated clinical and demographic data were obtained from the Oxford Brain Bank. Written informed consent for research use and publication was obtained in accordance with the ethical protocol approved by the South Central–Oxford C Research Ethics Committee (REC reference 15/SC/0639).

### Generation of hiPSC-derived midbrain dopaminergic neurons

All hiPSC lines were maintained in mTeSR1 media (Stem Cell Technologies, 85857) and regularly tested negative for mycoplasma contamination prior to differentiation. All hiPSC lines have been characterised previously (Supplementary Table 1)^3,5^. HiPSCs were passaged at 90-95% confluency using Versene (Gibco, 15040066) onto Geltrex (Life Technologies, A1413202) coated plates until reaching a suitable morphology and confluency for differentiation. No hiPSCs beyond passage 20 were used for any differentiation. HiPSC-DANs were generated as previously described^3^. Briefly, hiPSCs were directed down a midbrain floor plate lineage for 12 days via stimulation with 100 nM LDN193189 (Sigma, SML0559), 2 μM A83-01 (Tocris, 2939), 300 ng/ml Sonic Hedgehog (Shh—R&D Systems, 464-SH-200), 2 μM purmorphamine (Tocris, 4551), 200 ng/ml FGF8α (R&D Systems, 4745-F8) and 3 μM CHIR99021 (Tocris, 4423). After which they were directed down a DAN lineage until D21+ via stimulation with 20 ng/ml BDNF (PeproTech, 450-02), 20 ng/ml GDNF (PeproTech, 450-10), 1 ng/ml TGFß3 (Life Technologies, PHG9305), 10 μM DAPT (Tocris, 2634), 200 μM ascorbic acid (Sigma, A4403), 500 μM db-cAMP (Sigma, D0627) and 1 μg/ml laminin (Sigma, L2020). At D25, hiPSC-DANs were plated on Geltrex coated plates and simultaneously transduced with αSyn-APEX lentivirus at an MOI of 7 (infectious units/ml [IFU]: 1.9 × 10^6^; determined by Lenti-X™ qRT-PCR Titration Kit per manufacturer’s instructions [Takara Bio, 631235]) and 1 μg/ml polybrene (Merck, TR-1003-G) to improve transduction efficiency. Transduced hiPSC-DANs were maintained beyond D45, at which point we previously showed they are electrophysiologically active^5^, until their experimental endpoints.

### Generation of dCas9-KRAB SNCA-KO hiPSC-derived midbrain dopaminergic neurons

dCas9-KRAB *SNCA*^*TRIP*^ hiPSCs were generated by lentiviral transduction of dCas9-KRAB and M2rtTA into the AAVS1 locus (Supplementary Fig. 14 and 15). dCas9-KRAB hiPSCs were transduced with lentivirus expressing sgRNA against *SNCA* (target sequence = GGGAGTGGCCATTCGACGA) at an MOI of 5 (Lenti-X^TM^ p24 Rapid Titer Kit, Takara Bio 632200). Transduced hiPSCs were then selected with puromycin (Sigma, P8833) for 5 days or until 90-95% confluency was reached. No residual puromycin was detected at the time of testing (D50) as shown in Supplementary Fig. 15c. Transduced hiPSCs were differentiated into DANs as described above. Half of the culture was treated with 0.7 µg/ml doxycycline (Sigma, D3447) every 48 h from D25 to induce dCas9-KRAB expression. Transduced hiPSC-DANs were maintained until their experimental endpoint at D50.

### Generation of clonal αSyn-knockout SH-SY5Y cell line

*SNCA* crRNA (target sequence = GCTGCTGAGAAAACCAAACA) and an invariant 67mer tracrRNA were purchased from IDT. The crRNA and tracrRNA were combined (both at 1 µM concentration) in Nuclease-Free Duplex Buffer (IDT) and incubated at 95 °C for 5 min. Wild-type SH-SY5Y cells were reverse transfected in a 12-well plate with Cas9/*SNCA* gRNA complexes using Lipofectamine CRISPRMAX (Thermo Fisher Scientific). Per well, reagent amounts were as follows: 3 µg Alt-R Cas9-GFP V3 (IDT), 480 ng gRNA duplex, 5 µl Cas9 PLUS Reagent (Thermo Fisher Scientific) and 3 µl CRISPRMAX Reagent (Thermo Fisher Scientific). Three days later, a top-up transfection was performed using fresh Cas9/*SNCA* gRNA complexes. After a further four days, the polyclonal culture was harvested for single-cell clone isolation by limiting dilution. Clones were expanded and screened by immunoblotting.

### αSyn fibril generation, characterisation and seeding

#### Production of de novo-generated αSyn fibrils

Exogenous αSyn fibril synthesis and purification was conducted as we described previously^34^. The bacterial expression construct pRK172 encoding recombinant wild-type human αSyn was transformed into BL21 (DE3) *E. coli* (New England BioLabs, C2527H). Transformed cells were incubated for 4 h at 37 °C and 220 rpm, then plated out on 100 μg/ml ampicillin (Sigma, A0166) LB agar plates (Sigma, L2897) and incubated overnight at 37 °C. The following day, all successfully grown colonies were scraped into 2 L of 100 μg/ml ampicillin inoculated Terrific broth (Sigma, T0918). Bacteria were then cultured for 2.5 h at 37 °C and 220 rpm before adding 0.4 mM isopropyl β-d-1-thiogalactopyranoside (IPTG—VWR, 437144 N) to stimulate αSyn expression. Bacteria were then incubated for a further 5 h at 37 °C and 220 rpm. Bacteria were harvested by centrifugation at 4000 × *g* for 20 min at 4 °C, resuspended in a small volume of culture media, combined into one tube and then pelleted at 4000 × *g* for 20 min at 4 °C. Bacteria pellets were fully resuspended in low salt buffer [50 mM Tris-HCl, pH 7.4 (Santa Cruz Biotechnology, sc-301950), 20 mM NaCl (Sigma, S9888), 1 mM EDTA, 0.1 mM DTT, 0.1 mM phenylmethylsulfonyl fluoride (PMSF—Calbiochem, 52332), and 2x cOmplete™ Protease Inhibitor Cocktail tablets (Roche, 04693116001)] with 1 mg/ml lysozyme (Thermo Scientific, 89833), DNase (Invitrogen, 18047019) and RNase (Thermo Scientific, EN0601). Lysis was performed by sonication on ice [5 s on/5 s off, 40% amplitude, for 2 min and debris was centrifuged at 15,000 × *g* for 20 min at 4 °C. The supernatant was then extracted and purified via a two-stage high-performance liquid chromatography (HPLC) protocol. The lysate was first fractionated through a HiTrap™ Q HP anion exchange chromatography column (Cytiva, 17115401) (Supplementary Fig. 2a). Fractions with high αSyn content, determined by SDS-PAGE Coomassie stain and immunoblot, were pooled then fractionated through a HiLoad™ 16/600 Superdex™ 75 pg size exclusion chromatography column (Cytiva, 28-9893-33) (Supplementary Fig. 2b). Fractions with high αSyn content (determined by SDS-PAGE Coomassie stain and immunoblot) were pooled then spin concentrated by centrifugation [4000 × *g* for 8-12 h at 4 °C] using a Vivaspin™ column (Cytiva, 28-9323-58). Low salt buffer was then exchanged for 150 mM KCl buffer using the same Vivaspin™ column and centrifugation protocol. Purified protein concentration was determined by Pierce™ BCA assay kit (Thermo Scientific, 23225) per manufacturer’s instructions and NanoDrop™ spectrophotometer (Thermo Scientific, ND-ONE-W). Purified protein was diluted to a working concentration of 1 mM, aliquoted, snap frozen on dry ice and stored at −80 °C until further use.

#### Thioflavin T assay

Fibril formation was assessed by Thioflavin T (ThT—Abcam, ab120751) αSyn monomer (1 mM) or generated fibrils (D7) were mixed with 25 μM ThT diluted in PBS and fluorescence was measured using a CLARIOstar Plus Microplate Reader (excitation 450 nm, emission 485 nm). Raw fluorescence intensity values were then normalised to monomer to assess fold change (Supplementary Fig. 2c).

#### Transmission electron microscopy

De novo-generated αSyn fibril morphology was assessed by transmission electron microscopy (TEM). Fibrils were applied to freshly glow discharged carbon coated 300 mesh copper grids (TAAB Laboratories, C267/050) for 2 min, and then blotted with filter paper and stained with 2% uranyl acetate for 10 s, gently blotted again and air dried. Grids were imaged with a JEOL 1400 TEM at 120 kV using a Gatan Rio CMOS camera. Fibril morphology was quantified by length measurement in ImageJ (v1.54 f), with an average length of 79 nm post-sonication (Supplementary Fig. 2d, e).

#### Homogenous time resolved fluorescence (HTRF)™

Intraneuronal αSyn aggregation was assessed using the anti-phospho S129 αSyn HTRF™ kit (Revvity, 6FSYNPEG) as per manufacturer’s instructions. Donor and acceptor FRET fluorophores were mixed with neuronal lysate and incubated for 24 h in the dark. Fluorescence intensity was measured using a CLARIOstar Plus Microplate Reader (BMG Labtech, Ortenberg, Germany), samples were excited at 340 nm and emission values were measured at 620 nm and 665 nm. FRET was determined by calculating the 665/620 nm ratio with αSyn aggregation being detected as a background-subtracted fold increase over unseeded controls (%∆F).

### Seeding of neuronal cultures

De novo-generated αSyn fibrils were tested in hiPSC-DANs^3^. Fibrils were diluted to 1 µM in neuronal media without growth factors and sonicated on ice for 2 min, 1 s on/1 s off, 40% amplitude. Fibril seeds were then diluted to a final concentration of 350 nM in neuronal media with growth factors for fibril seeding in hiPSC-DANs at D45. Media were changed 48 hrs later.

### Plasmid synthesis and lentivirus generation

A C-terminal APEX2-tagged αSyn construct was generated by introducing full-length wild-type human αSyn cDNA (ENST00000336904.7) into the mito-V5-APEX2 pcDNA3 vector (Addgene, 72480) after removal of the mito-tag using the restriction enzyme sites NotI and BamHI. The αSyn-APEX2 construct and an *SNCA* oligo (5′-CACC GGGAGTGGCCATTCGACGAC-3′), which targets the main *SNCA* promoter, were cloned into pLenti CMVie-IRES-BlastR (Addgene, 119863) and the CRISPRi vector LentiGuide-Puro (Addgene, 52963), respectively, for lentiviral expression. Separately, chemically synthesised gBlock gene fragments encoding wild-type, Δ10–20, Δ21–31 or Δ32–42 αSyn fused at its C-terminus via a GGGGS linker to FLAG (DYKDDDDK) were purchased from IDT. The αSyn open-reading frame in a pre-existing lentiviral expression plasmid, pLenti-CMV-WTαSyn-BlastR^19^, was replaced with the gBlock fragments using NEBuilder HiFi DNA Assembly Master Mix (NEB, E2621S). All constructs were validated by Sanger sequencing (Eurofins).

Lentiviruses were produced by transfecting HEK293T cells with the packaging plasmid psPAX2 (Addgene, 12260), the envelope plasmid pMD2.G (Addgene, 12259), and the appropriate transfer plasmid (CMVie-αSyn-APEX, the *SNCA* CRISPRi vector, or the FLAG-αSyn expression vectors) using polyethylenimine. The culture medium was replaced the following day and collected after an additional two days. Conditioned media were spun at 400 × *g* for 5 min, and the supernatant was filtered through 0.22 μm PES. Lentiviral particles were purified and concentrated 100-fold using the Lenti-X™ Concentrator kit (Clontech, PT441-1). Viral titre was determined by Lenti-X™ Provirus Quantitation Kit (Takara Bio, 631239) per manufacturer’s instructions.

### APEX proximity-dependent biotinylation labelling

At D3-PS, D7-PS, or D14-PS αSyn-APEX transduced hiPSC-DANs were treated with 500 μM Biotinyl Tyramide (Tocris, 6241) for 30 min following existing protocols^4^. Biotinylation was then stimulated with 1 mM H_2_O_2_ in PBS added directly to the media for 1 min followed by 3x quick rinses in quenching solution consisting of 10 mM sodium ascorbate (VWR, 95035-692) 5 mM Trolox (Sigma, 238813-5G), and 10 mM sodium azide (VWR, AA14314-22) in PBS with calcium and magnesium (DPBS—Gibco, 14040133). Cells were then rinsed once more with PBS before being lysed in RIPA [50 mM Tris-HCl, pH 7.4 (Santa Cruz Biotechnology, sc-301950), 500 mM NaCl (Sigma, S9888), 0.1% SDS (Sigma, L3771), 5 mM EDTA (Invitrogen, 15575020), 1 mM DTT (Roche, 10708984001) and 1x protease inhibitor tablet (Roche, 11697498001) in dH_2_O], sonicated [10 pulses, 1 s on/1 s off, 40% amplitude], and centrifuged [15 min, 15,000 rpm, 4 °C]. Soluble fractions were transferred to a new tube for co-immunoprecipitation with Pierce™ High-Capacity Streptavidin Agarose beads (Thermo Scientific, 20357) overnight at 4 °C on a rotator following manufacturer’s instructions. The following morning, samples were briefly centrifuged to pellet the beads after which the unbound fraction was removed and retained for later validation. Beads were then washed following an existing protocol optimised for APEX biotinylated proteins in neurons^48^. Briefly, beads were washed 2x in 0.1% RIPA, 1x in 1 M KCl (Sigma, P5405), 1x in 0.1 M sodium carbonate (Sigma, S7795), 2x in 2 M urea (Sigma, U5378), 2x in 0.1% RIPA; bound proteins were then eluted [95 °C, 1000 rpm, 10 min in 4X NuPAGE LDS Sample Buffer (Invitrogen, NP0007) supplemented with 2 mM Biotinyl Tyramide and 20 mM DTT. Successful immunoprecipitation of biotinylated proteins was confirmed by blotting with streptavidin-horseradish peroxidase conjugate (HRP—Invitrogen, S911) and the eluted fractions were submitted for mass spectrometry.

### Mass spectrometry data acquisition

Samples (*n* = 3 independent differentiations) were processed using an S-Trap™ micro kit (Protifi, K02-mini-10). Samples were first adjusted to 5% SDS final concentration. Reduction and alkylation were performed with 10 mM tris (2-carboxyethyl)phosphine (TCEP) and 50 mM iodoacetamide. Samples were then acidified with phosphoric acid to 1.2% final concentration and diluted (1:7 ratio) with 90% methanol/100 mM tetraethylammonium bromide (TEAB). Samples were transferred to S-TRAP spin columns, centrifuged [4000 × *g*], and then washed 4 times with 150 µl 90% methanol/100 mM TEAB [4000 × *g*]. 1.5 µg sequencing grade trypsin (Promega, V511A) in 50 mM TEAB was then added to the spin column to digest samples overnight at 37 °C. The following day, peptides were eluted with 150 µl 0.1% formic acid and 150 µl 50% acetonitrile/0.1% trifluoro acid and then dried in a centrifugal evaporator.

Peptides were resuspended in 5% formic acid and 5% DMSO and then trapped on an Acclaim™ PepMap™ 100 C18 HPLC column (300 µm × 5 mm, 5 µm particle size, Thermo Fisher Scientific) using solvent A (0.1% Formic Acid in dH_2_O) at a pressure of 60 bar and separated on an UltiMate™ 3000 UHPLC system (Thermo Fisher Scientific) coupled to a Q Exactive Orbitrap™ mass spectrometer (Thermo Fisher Scientific). The peptides were separated on an EASY-Spray PepMap RSLC column (75 µm i.d. × 2 µm × 50 mm, 100 Å, Thermo Fisher Scientific) and then electro-sprayed directly into a Q Exactive Orbitrap™ mass spectrometer (Thermo Fisher Scientific) through an EASY-Spray™ nano-electrospray ion source (Thermo Fisher Scientific) using a linear gradient (length: 60 min, 5% to 35% solvent B (0.1% formic acid in acetonitrile and 5% DMSO), flow rate: 250 nl/min). Raw data were acquired in data-independent mode (DIA). Full scan MS spectra were acquired in the Orbitrap with inclusion list; scan range 495–995 m/z, 20 m/z increments, with an overlap of ±2 Daltons, resolution 35,000, AGC target 3e6, maximum injection time 55 ms. MS scan peaks were selected for high-energy collisional dissociation (HCD) fragmentation at 28% of normalised collision energy (NCE)/stepped NCE. HCD spectra were also acquired in the Orbitrap (resolution 17,500, AGC target 1e6, isolation window 20 m/z).

### Proteomic network analyses and visualisation

Quantification was performed using DIA-NN (v1.8.1) in library free mode using standard parameters filtered at 1% false discovery rate. Database used: UPR_Homo sapiens_9606_UP000005640.fasta with carbamidomethyl C and oxidation (M) search modifications and match between runs enabled. Data were normalised by median centring and missing values were imputed by random selection form a down-shifted normal distribution. Peptides detected in 5 of 6 biological replicates in each condition were included for quantitative analysis. Biological replicates were grouped and a multiple correction Student’s *t* test using a permutation-based FDR of 0.05 was performed to determine statistical significance. Data were quantitatively analysed in Perseus (version 1.6.15.0). Proteins identified by mass spectrometry that were found to be ±1 log2 fold change in differential interaction were included for network analyses in all conditions. Gene Ontology (GO) analyses were performed using ShinyGO (v0.77) with a false discovery rate cutoff of 0.05 kept constant for all GO analyses. Network analyses were generated from two ontologies (GO: Biological Process and GO: Cellular Component) and KEGG pathway analysis. Protein-protein interaction (PPI) networks were mapped using STRING-db (v12.0). Only interactions with high confidence (0.700) were included with a medium false discovery rate of 5%. Disconnected nodes were removed from all networks. *K*-means clustering was then performed using STRING-db to identify communalities between PPI clusters. All STRING networks were imported into Cytoscape (v3.10.1) using the StringApp plugin for conversion into figure diagrams. None of the key interactome proteins of interest investigated herein were detected in the Contaminant Repository for Affinity Purification (CRAPome) database (v2.0—H. sapiens, Proximity Dependent Biotinylation dataset).

### AlphaFold structural interaction analysis

Structural data for the human Sec61A complex, were acquired from the RCSB Protein Data Bank (PDB - ID: 8DNV^50^). AlphaFold structures were initially visualised using the PyMOL Molecular Graphics System (Schrödinger, LLC; v4.6) then colour-coded in UCSF ChimeraX (v1.6.1). All structure predictions were generated used ColabFold, run locally on a Dell Precision 5820 workstation installed with an Nvidia RTX4000 GPU card. Each run of AlphaFold produces 5 structures and to generate a total of 100 structures, we ran each prediction 20 times, each with a different random seed. We used 10 recycles through the network to improve the prediction and the *--amber* flag to relax the predicted structure using the AMBER force field. The structures generated were ranked using pTM (estimate of the Template Modelling score).

### Bulk RNA sequencing

Bulk RNA-seq was performed as previously described^3^ for *SNCA*^*TRIP*^-DANs cultures at D52 and D60. RNA samples were prepared using standard poly-A enrichment, 250–300 bp insert cDNA library was prepared with the NEB Next® Ultra™ RNA Library Prep Kit (New England Biolabs, E7530) and sequenced on Illumina NovaSeq 6000 (S4) using 150 bp paired-end reads. Sequencing resulted in ~40 million reads per sample. Bulk RNA-seq data are available at GEO under accession numbers GSE276377 and GSE171999.

Kallisto (v0.46.2)^51^ was used to estimate abundance for Ensembl (release 88) protein-coding genes from RNA-seq reads. Count data were imported into R (v4.0.3) using Tximport (v1.18.0), genes with <10 reads across the six samples used in the comparison were filtered out. Differential gene expression was performed in DESeq2 (v1.30.0)^52^ using the Wald test with a FDR threshold of 0.05 and a log2 fold-change threshold of 0.25. GSEA was performed using the Fgsea package (v1.16.0)^53^ in R (v4.0.3). Genes were ranked for GSEA using the Wald statistic and Fgsea (v1.16.0) was run with 1000 permutations. Principal component analysis and all plotting for bulk RNA-seq data were performed in R (v4.0.3).

### Single cell RNA sequencing

Cells were dissociated in Accutase for 30 min at 37 °C to generate a single cell suspension. Cells were fixed in 100% methanol following the 10X Genomics established protocol. Cell suspensions containing a total of 36,689 cells were processed by the Chromium Controller (10X Genomics) and barcoded libraries constructed using the Chromium Single Cell 3’ Reagent Kit v3 (10X Genomics, PN-1000268), following manufacturer’s instructions. Libraries were sequenced with NovaSeq 6000 (Illumina) as summarised in Supplementary Data 4. Cells were annotated by mapping to Welch et al. 2019 reference dataset^54^ using SingleR^55^. Stressed cells were identified using Gruffi^56^. Only DANs that are not stressed were used for downstream analysis. Integration was performed using the STACAS package^57^ and clustering was done using the original Louvain algorithm implemented in the Seurat package^58^.

Single-cell RNA sequencing data were analysed using the Slingshot algorithm (slingshot package) to infer cell trajectories based on UMAP-reduced dimensions. Data were converted from Seurat to SingleCellExperiment format. The Slingshot wrapper function was used to simultaneously perform global lineage structure identification and fit principal curves to describe each lineage. This was achieved by running slingshot^59^ with UMAP-reduced dimensions and cluster labels for the two experimental conditions: *SNCA*^*ISO*^ and *SNCA*^*TRIP*^. Pseudotime trajectories were visualised using UMAP embeddings coloured by pseudotime values and trajectory lines.

To identify dynamically expressed genes, generalised additive models were fitted using the tradeSeq package. The association between gene expression and pseudotime was tested with associationTest, and the top 200 dynamically expressed genes were selected based on *p*-values. Gene expression patterns were visualised using heatmaps. Single cell RNA-seq data are available at GEO under accession number GSE276559.

### DSG crosslinking and co-immunoprecipitations

For sample crosslinking, cells transduced with FLAG-αSyn lentiviruses were detached using TrypLE and pelleted by centrifugation at 300 × *g* for 5 min. Cell pellets were washed with PBS before resuspension in PBS containing 0.5 mM DSG (Thermo Scientific, A35392) and 1x cOmplete™ Protease Inhibitor Cocktail (Roche, 11697498001). Cells were incubated with the DSG crosslinker for 30 min at 37 °C with rotation. Excess crosslinker was quenched by adding 1 M Tris, pH 7.4, to a final concentration of 50 mM and incubating for a further 15 min at room temperature with rotation. Efficient protein extraction was ensured by sonicating [3 pulses, 5 s on/5 s off, 40% amplitude], adding Triton X-100 to a final concentration of 0.5%, and incubating for 15 min at 4 °C with rotation. For co-immunoprecipitations, Dynabeads Protein G (Invitrogen, 10003D) were first incubated with 3 µg anti-Sec61A (Abcam, ab183046) per reaction according to the manufacturer’s instructions. Antibody-beads complexes were incubated with 800 µg cell lysate overnight at 4 °C with rotation. Washes and denaturing elution steps were performed according to the manufacturer’s instructions.

### Immunoblotting

Cells in all immunoblotting experiments were lysed in Pierce RIPA (Thermo Scientific, 89900), and 1x cOmplete™ Protease Inhibitor Cocktail (Roche, 11697498001) and PhosSTOP (Roche, 4906845001), sonicated [10 pulses, 1 s on/1 s off, 20–40% amplitude], and centrifuged [15 min, 15,000 rpm, 4 °C]. Protein concentration was determined by BCA. Samples were resolved by SDS-PAGE (Invitrogen, NW04127BOX), transferred to a nitrocellulose membrane (Invitrogen, IB23002) using an iBlot™ 2 Gel Transfer system (Invitrogen, IB21001). For dot blotting, samples were spotted directly onto nitrocellulose membrane. Membranes were blocked for 1 h in 5% bovine serum albumin (Melford, A30075) in 1X TBS with 0.1% Tween-20 (Sigma, P1379). Membranes were then immunoblotted with primary antibodies overnight at 4 °C, followed by incubation with anti-mouse or anti-rabbit horseradish peroxidase (HRP) conjugated secondary antibodies for 1 h at RT. Membranes were then incubated in ECL™ Start Western Blotting Detection Reagent (Cytiva, GERPN3244) for 5 min and subsequently scanned using a ChemiDoc Imaging system (Bio-Rad). Band intensity was quantified by densitometry using Image Lab software (Bio-Rad, v6.1). Primary antibodies used for immunoblotting are summarised in Supplementary Table 2.

### Proximity ligation assay

#### HiPSC-DANs

All PLA was carried out on 8-well Removeable Chamber Slides (Ibidi: 80841) or 13 mm 1.5H High Precision coverslips (Marienfeld, 0117530). Duolink™ In Situ Red Starter Kit Mouse/Rabbit was used (Sigma, DUO92101) for all PLA reactions. All PLA was carried out according to Duolink™ manufacturer protocol. Briefly, cells were fixed in 4% formaldehyde in DPBS solution with 4% sucrose for 15 min and permeabilized with DPBS (Sigma, T8787) containing 0.1% Triton-X-100 for 30 min at RT. Permeabilized neurons were blocked in Duolink™ Blocking Solution for 1 h at 37 °C. Primary antibodies were diluted in Duolink™ Antibody Diluent and neurons were incubated with primary antibodies overnight at 4 °C. The following day, neurons were washed twice with 1X Wash Buffer A for 10 min each. Duolink™ PLUS and MINUS PLA Probes (1:5 dilution) together with secondary antibodies were diluted in Duolink™ Antibody Diluent accordingly. Neurons were incubated in the PLA probe, washed with 1X Wash Buffer A for 10 min each and were subsequently incubated with 1X Ligation buffer with ligase for 30 min at 37 °C. After ligation, neurons were washed with 1X Wash Buffer A for 10 min each and were incubated in 1x Amplification buffer for 100 min. After amplification, neurons were washed twice with Wash Buffer B for 10 min each followed by a final wash with 0.01X Wash Buffer B for 1 min. Neurons were mounted with Duolink™ In Situ Mounting Medium with DAPI (Sigma, DUO82040) and then sealed with CoverGrip™ Coverslip Sealant (Biotium, 23005).

#### Human brain tissue

All PLA experiments for brain tissue samples were carried out according to the Duolink™ manufacturer protocol. Briefly, paraffin-embedded tissues were incubated at 60 °C for 30 min and subsequently dewaxed in xylene, followed by rehydration in alcohol correspondingly. Antigens were retrieved by microwave heating of Tris-EDTA buffer (pH 9) for 10 min. Endogenous peroxidase activity was quenched using Hydrogen Peroxide for 10 min and tissues were subsequently blocked in Duolink™ Blocking Solution for 1 h at 37 °C. Primary antibodies were diluted in Duolink™ Antibody Diluent and tissues were incubated were incubated with primary antibodies overnight at 4 °C. The following day, samples were washed twice with 1X Wash Buffer A for 5 min each and incubated in the PLA probe solution for 1 h at 37°, washed with 1X Wash Buffer A for 5 min each and were subsequently incubated with 1X Ligation buffer with ligase for 30 min at 37 °C. After ligation, tissues were washed with 1X Wash Buffer A for 2 min each and were incubated in 1X Amplification buffer for 120 min. Tissues were then washed twice with 1X Wash Buffer A for 2 min each and incubated with Brightfield detection solution for 60 min at room temperature. After washing with Wash Buffer A twice, tissues were developed in Substrate reagent for 20 min with the dilution of Substrate Reagents A (1:70), B (1:100), C(1:100) and D (1:50) in UltraPure™ DNase/RNase-free distilled water. Tissues were then counterstained with hematoxylin, dehydrated in alcohol and xylene, and mounted in DPX mounting media. Mounted slices were imaged on an EVOS™ M7000 microscope using a 40X objective (Invitrogen, AMF7000). Patient information on all human brain tissue samples used can be found in Supplementary Table 3.

### Isolation of extracellular vesicles

A total volume of 4 ml conditioned media was collected from *SNCA*^*ISO*^ and *SNCA*^*TRIP*^ DANs at D45, D60 and D70 timepoints. Removal of cells and cell debris was performed by serial centrifugation steps at 300 g for 10 min followed by 4000 g for 10 min. The pre-cleared conditioned media were then concentrated 4-fold to obtain a final volume of 1 ml. Size exclusion chromatography was performed on the concentrated conditioned media using the HiScreen Capto Core 700 column (17548115, Cytiva) and the NGC chromatography system (BioRad) for the isolation of EVs. The EVs were collected in fractions 3 and 4 as previously^60^. The EV fractions were then pooled and further concentrated from 2 ml to 50 µl using the Amicon Ultra centrifugal filters with a 3 kDa molecular weight cut off (UFC200324, Millipore) at 4000 g for 2.5 h at 4 °C. The EVs were then lysed by addition of 10 µL of 6% Triton X-100 (T8787, Sigma-Aldrich) containing protease inhibitors (78429, Thermo Scientific) with 900 RPM shaking for 30 min at room temperature. Lysates were stored in −80 °C until electrochemiluminescence measurement performed in 96-well Meso Scale Discovery U-Plex plates. Wild-type M17D or αSyn overexpressing M17D cells were plated into 10 cm dishes at a density of 5 × 10^5^ cells per dish. Upon reaching confluency, cells were then treated for 48 h with 200 nM of the lysosomal v-ATPase inhibitor bafilomycin A1 (1334, Tocris) in serum free media. The non-treated control cells received serum-free media without bafilomycin A1. Conditioned media were collected for EV isolation as previously described. Following media collection, cells were lysed with Pierce RIPA (89900, Thermo Scientific) containing protease inhibitors (78429, Thermo Scientific) and the cell lysates were used for western immunoblotting as previously described.

### Subcellular fractionation

Cytosolic lysis buffer (2% Saponin, 10 mM HEPES, 300 mM sucrose, 100 mM NaCl, 5 mM MgCl2, 5 mM EDTA) and membrane lysis buffer (0.5% Triton X-100,10 mM HEPES, 300 mM sucrose, 100 mM NaCl, 5 mM MgCl2, 5 mM EDTA) with Pierce™ Protease Inhibitor (Thermo Scientific, A32955) were prepared fresh. For subcellular fractionation, cells were rinsed briefly with DPBS and subsequently incubated with cytosolic lysis buffer at 4 °C for 20 min with gentle rocking. The cytosolic fraction was collected without disrupting the cell monolayer. Cells were rinsed briefly with DPBS solution again and then incubated with membrane lysis buffer at 4 °C for 15 min with gentle rocking. The cytosolic fraction and membrane fraction were then centrifuged at 1000 × *g* for 5 min at 4 °C to pellet cell debris. Extracted supernatants were then assessed by immunoblotting.

### Microscopy

#### Immunocytochemistry

Cells were rinsed 1x in DPBS, then immediately fixed in a 4% formaldehyde/2% sucrose in DPBS solution. Cells were then permeabilized and blocked simultaneously in DPBS containing 0.1% Triton-X-100 (Sigma, T8787) and 2% Normal Goat Serum (NGS—Cell Signalling Technology: 5425S) for 1 h at RT. Primary antibody was added overnight at 4 °C in a humidified chamber and washed in DPBS 3x for 15 min at RT the following day. Cells were stained with secondary antibodies in a lightproof humidified chamber for 1 h at RT, washed in DPBS 3x for 15 min at RT under lightproof conditions, then mounted in ProLong Glass Antifade Mountant with DAPI to stain nuclei (Invitrogen, P36981) and left to set in lightproof conditions for at least 48 h prior to sealing with nail varnish and subsequent imaging. Primary antibodies used for immunocytochemistry are summarised in Supplementary Table 2.

#### Immunohistochemistry

Antigens were retrieved by steaming of of Tris-EDTA buffer (pH 9, 0.05% Tween-20) for 30 min. Slides were then blocked with 5% BSA at room temperature. Primary antibody was added overnight at 4 °C in a humidified chamber, washed 2× in TBST and 1x in TBS, and subsequently incubated with secondary antibodies for 1 h 30 min at room temperature. Samples were then washed 2× in TBST and 1x in TBS, followed by autofluorescence quenching with Thermo Fisher Autofluorescence Quenching Reagent (Thermo, R37630) for 10 min. Samples were then washed and mounted with Prolong Glass Antifade Mountant.

#### Super-resolution microscopy

All images of fixed cells were acquired using a Zeiss LSM980 laser scanning confocal coupled to an Airyscan 2 module equipped with an Olympus Plan-Apochromat 63 × 1.43 NA oil-immersion lens and a 32-channel gallium arsenide phosphide (GaAsP-PMT) area detector. Z-stacks were acquired for all images, multiple images per stack were collected spaced apart by 0.13 μm (optimised for Airyscan acquisition) operated by a Wienecke & Sinske PiezoDrive CAN stage controller. All stacks were post-processed using an Airyscan algorithm built into Zen Blue (v3.3.89) and then quantified in Fiji (ImageJ). Super-resolved structured illumination microscopy (SIM) images were collected using a VisiTech-iSIM module coupled to an Olympus IX83 microscope with an Olympus UPlanApo 100x HR TIRF oil immersion objective (NA:1.50) and a Hamamatsu ORCA-Quest qCMOS camera. Imaging data were deconvolved using the constrained iterative Gold deconvolution algorithm for 10 iterations to increase contrast and resolution without inducing imaging artefacts in cellSens Dimension (Olympus, v4.2.1).

#### Live-cell microscopy

All live-cell imaging was conducted using a VisiTech-iSIM module coupled to an Olympus IX83 microscope with an Olympus UPlanApo 100x HR TIRF oil immersion objective (NA:1.50) and a Hamamatsu ORCA-Quest qCMOS camera. Temperature/humidity were maintained at 37 °C and 5% CO_2_ before and during image acquisition using a live-cell environmental chamber (OKO Lab Bold Line 3). All HiPSC-DANs for live imaging were plated on 8-well #1.5H µ-Slide glass bottom chamber slides (Ibidi, 80807-96). For the Magic Red® Cathepsin B Assay, cells were incubated with Magic Red (1:25 of the diluted stock) for 30 min followed by 10 min incubation of Hoechst 33342. Samples were washed 2 time with PBS before imaging in VisiTech-iSIM. Live-cell ratiometric lysosomal pH was quantified using established protocols^61^, modified for hiPSC-DANs. HiPSC-DANs were plated on 8-well #1.5H µ-Slide glass bottom chamber slides to generate calibration curves for each time point and each cell line or for their corresponding experimental conditions. HiPSC-DANs were first incubated at 37 °C and 5% CO_2_ overnight in neurobasal media supplemented with growth factors and 100 µM Dextran Alexa Fluor™ 555 (Invitrogen, D34679). This was then followed by 15 min incubation with 0.5 µM LysoSensor™ Green DND-189 (Invitrogen, L7535). Once loaded with dyes, chamber slides were moved to the VisiTech-iSIM microscope and incubated with calibration buffers (pH 4.5–6.5) for 5 min. Calibration buffers were made following existing recipes, including 10 mM nigericin and monensin. After 5 min, 3–5 Z-stacks of 5–15 cells were acquired per condition for all calibration and experimental conditions at all time points and all cell lines. For the experimental conditions, the imaging buffer was replaced with pH 7.4 artificial CSF without nigericin/monensin, following existing recommendations.^61^

### Image quantification

#### Identity marker, fibril seeding and colocalization quantification

HiPSC-DAN markers were quantified in ImageJ. Five images per condition per differentiated iPSC line for each experiment and 3 biological replicates were acquired, projected and converted to 8-bit images. Briefly, cell counts for all images were acquired by counting DAPI positive nuclei. Neuronal cell count was calculated by counting DAPI and MAP2 positive cells. Identity marker positive cells were counted as DAPI, MAP2 and identity marker positive cells and percentages of marker positive cells were calculated and averaged in Excel. An identical pipeline was used for V5 positive cells to calculate successful αSyn-APEX transduction percentage in hiPSC-DANs. For fibril seeding in hiPSC-DANs, 5 images per condition per differentiated iPSC line for each experiment and 3 biological replicates were acquired, projected, and converted to 8-bit images. Regions of interest were drawn containing DAPI and MAP2 positive cells. pSyn positive foci within each region of interest were counted and measured by area to quantify the degree of fibril seeding at each time point. For PLA, a total of 100–120 cells from 5 images per condition per differentiated IPSC line were acquired and this was repeated in total of 3–6 biological replicates. PLA Punta was quantified via find maxima function and was shown as fold changes by normalising to its control per set of differentiation. Colocalization of proteins of interest with key subcellular organelles was quantified in ImageJ via JACoP v2.0 (Just Another Colocalization Plugin). Five images per condition per protein/organelle of interest per iPSC line for each experiment and 3 biological replicates were acquired, projected and converted to 8-bit images. Images were subjected to a threshold to binarize the image intensity values; threshold values were kept consistent throughout the analysis where necessary. Colocalization was then determined by Pearson’s coefficient.

#### Subcellular pH

Calibration curves were generated by quantifying fluorescence intensities from lysosomal regions of interest and plotting these against the corresponding pH values, followed by linear regression fitting. Lysosomal pH values for *SNCA*^*ISO*^ and *SNCA*^*TRIP*^ hiPSC-DANs were then derived using their respective calibration curves. Background signal for 488 was also quantified per condition by averaging signal intensity from three independent regions of interest where no cell body or cell protrusions were visible. Background values were subtracted from the fluorescent signal to improve the signal-to-noise ratio of each dye probe.

#### 3D reconstruction

Three-dimensional reconstructions were generated from image stacks using Imaris software (Bitplane, Oxford Instruments). Z-stack image data were imported into Imaris and processed to create volumetric renderings of the sample. Image contrast and brightness were adjusted uniformly across all datasets for visualisation purposes, and the dataset was reconstructed in 3D using the Surpass view.

### Statistical analysis

All statistical analyses were conducted using GraphPad Prism (v9.0), excluding proteomic and RNA-sequencing analyses. Results were considered to be statistically significant when *p* < 0.05. All datasets were tested for normality prior to inferential statistical analyses. Differences between normally distributed groups were tested for statistical significance using independent-sample two-tailed *t*-test or one-way ANOVA with Tukey correction where applicable. Differences between non-normally distributed groups were tested for statistical significance using Mann-Whitney *U*-test or Kruskal-Wallis test with Dunn’s correction where applicable. All experiments were repeated for at least three biological replicates unless stated otherwise. All data and error bars are shown as mean ± standard error of the mean to two decimal places and each data point on all graphs represent one biological replicate. For all hiPSC-DAN experiments, one biological replicate was defined as one independent differentiation from hiPSC to DAN, the same clones for each line were used throughout. All investigators were blinded during data acquisition and analysis.

### Reporting summary

Further information on research design is available in the Nature Portfolio Reporting Summary linked to this article.

## Supplementary information

Supplementary Information

Description of Additional Supplementary Files

Supplementary Data 1

Supplementary Data 2

Supplementary Data 3

Supplementary Data 4

Reporting Summary

Transparent Peer Review file

## Source data

Source Data 1

## Data Availability

Supplementary Information is available for this paper. All data supporting the findings of this manuscript are available from the corresponding author upon request. Correspondence and requests for materials should be addressed to george.tofaris@ndcn.ox.ac.uk. Proteomic data have been deposited to the ProteomeXchange Consortium via the PRIDE partner repository with the dataset identifier PXD055581. Bulk RNA-seq data are available at GEO under accession number GSE276377 and GSE171999. Single cell RNAseq data are available at GEO under accession number GSE276559. Source data are provided with this paper. No custom code was generated in this study. Source data are provided with this paper.
