# Supplementary material for: α-Synuclein blocks endoplasmic reticulum co-translational protein translocation early in Parkinson’s disease
Source: Nat Commun. 2026 Aug 6;17:7768. doi: 10.1038/s41467-026-76173-4 (PMC13448182; doi:10.1038/s41467-026-76173-4)

Supplementary information for

α-Synuclein blocks endoplasmic reticulum co-translational protein translocation early in Parkinson's disease

Chor Lai Lam, Nicholas J. F. Gatford, Ana Aragón-González , Benedict Tanudjojo, Anis Sahoo Andrew R. Castle, Devika Agarwal, Ashwin Jainarayanan, Svenja S. Hester, Navoneel Sen, Justin L. P. Benesch, Roman Fischer, David Sims, George K. Tofaris*

* = corresponding author: george.tofaris@ndcn.ox.ac.uk

This PDF file includes:

Supplementary Tables 1-3

Supplementary Figures 1 -15

Source Data for Supplementary Figures

ID	Diagnosis	*SNCA* genotype	Sex	Biopsy age (years)	Original ID	Reprogramming method	iPSC clone characterisation	GEO
HC1	Healthy control	WT/WT	M	49	OX3-9	CytoTune1	Dafinca et al., 2016<br><br>doi:10.1002/stem.2388	GSE64584
HC2	Healthy control	WT/WT	F	67	SFC840-03-01 (AH017)	CytoTune1	Fernandez et al., 2016<br><br>doi:10.1016/j.stemcr.2016.01.013	GSM1831382
$SNCA^{ISO}$	PD	Isogenic WT/WT	F	55	Clone 1-13	CytoTune1	Heman-Askah et al., 2017<br><br>doi:10.1093/hmg/ddx331	GSE89886
$SNCA^{TRIP-3}$	PD	Triplication/WT	F	55	SFC831-03-05	CytoTune1	Haenseler et al., 2017<br><br>doi:10.1016/j.stemcr.2017.05.017	GSE89886
$SNCA^{TRIP-4}$	PD	Triplication/WT	F	55	ND34391G	CytoTune1	Heman-Askah et al., 2017<br><br>doi:10.1093/hmg/ddx331	GSE89886
$SNCA^{A53T}$	PD	A53T/WT	M	46	SFC829-03-06	CytoTune1	Haenseler et al., 2017<br><br>doi:10.1016/j.stemcr.2017.05.017	GSE89886
$SNCA^{A53T}$	PD	A53T/WT	F	51	SFC828-03-09	CytoTune1	Haenseler et al., 2017<br><br>doi:10.1016/j.stemcr.2017.05.017	GSE89886
dCas9-$SNCA^{TRIP-3}$	PD	Triplication/WT	F	55	SFC831-03-05	CytoTune1	This study	N/A

Supplementary Table 1: Cell line information for all hiPSC lines used in this study.

HC = healthy control, PD = Parkinson’s disease.

Epitope	Species	Clonality	Clone Number	Company Name	Catalogue Number	Dilution
Streptavidin, Alexa Fluor™ 488	-	-	-	Invitrogen	S11223	1:1000
Streptavidin, Alexa Fluor™ 568	-	-	-	Invitrogen	S11226	1:1000
V5	Mouse	Monoclonal	SV5-Pk1	Invitrogen	R960-25	1:500
V5	Rabbit	Monoclonal	SY30-01	Invitrogen	MA5-32053	1:500
Tuj1	Mouse	Monoclonal	TUJ1	BioLegend	801201	1:1000
Tuj1	Rabbit	Polyclonal	-	BioLegend	802001	1:1000
Alpha-synuclein	Mouse	Monoclonal	42	BD Transduction Laboratories	610787	1:1000
Alpha-synuclein (phospho S129)	Rabbit	Monoclonal	EP1536Y	Abcam	ab51253	1:1000
MAP2	Chicken	Polyclonal	-	Abcam	ab92434	1:1000
DAT	Rat	Monoclonal	DAT-Nt	Merck	MAB369	1:500
TH	Mouse	Monoclonal	LNC1	Merck	MAB318	1:1000
GFAP	Rabbit	Polyclonal	-	Novus	NB300-141	1:1000
SOX2	Rabbit	Polyclonal	-	Abcam	ab97959	1:1000
Ctip2	Rat	Monoclonal	25B6	Abcam	ab18465	1:500
Puromycin	Mouse	Monoclonal	12D10	Merck	MABE343	1:5000
Sec61A	Rabbit	Monoclonal	EPR14379	Abcam	ab183046	1:1000
Sec61A	Goat	Polyclonal	-	Abcam	ab1327	1:1000
Sec61B	Rabbit	Polyclonal	-	Proteintech	15087-1-AP	1:1000
ATP6V0a1	Rabbit	Polyclonal	-	Proteintech	13828-1-AP	1:1000
ATP6V0a1	Rabbit	Polyclonal	-	Novus	NBP1-89342	1:1000
UFM1	Rabbit	Monoclonal	EPR4264(2)	Abcam	ab109305	1:1000
UFM1	Sheep	Polyclonal	-	Bio-Techne	AF8237	1:1000
VAMP2	Rabbit	Monoclonal	EPR12790	Abcam	ab181869	1:1000
UGP2	Rabbit	Polyclonal	-	Proteintech	10391-1-AP	1:1000
LAMP2	Goat	Polyclonal	-	Bio-Techne	AF6228	1:1000
GBA	Rabbit	Polyclonal	-	Sigma	G4171	1:1000
SQS (FDFT1)	Rabbit	Polyclonal	-	Proteintech	13128-1-AP	1:1000
GM130	Sheep	Polyclonal	-	Bio-Techne	AF8199	1:1000
Tom20	Mouse	Monoclonal	F-10	Santa Cruz Biotechnology	sc-17764	1:1000
Calnexin	Mouse	Monoclonal	6F12BE10	abcam	ab112995	1:1000
Cathepsin B	Rabbit	Polyclonal	-	Proteintech	12216-1-AP	1:1000
Phospho-p38 MAPK (Thr180/Tyr182)	Rabbit	Polyclonal	-	Cell Signaling Technology	9211	1:1000
p38 MAPK	Rabbit	Polyclonal	-	Cell Signaling Technology	9212	1:1000
XBP1S	Rabbit	Polyclonal	-	Proteintech	24868-1-AP	1:1000
ATF4	Rabbit	Polyclonal	-	Proteintech	10835-1-AP	1:1000
CHOP	Rabbit	Polyclonal	-	Proteintech	15204-1-AP	1:1000
PSMD11	Mouse	Monoclonal	AT1F4	GeneTex	GTX50018	1:1000
β-Actin	Mouse	Monoclonal	AC-15	Sigma	A1978	1:5000
Cas9	Rabbit	Polyclonal	-	Takara Bio	632607	1:1000
TetR	Mouse	Monoclonal	-	Takara Bio	631132	1:1000
Anti-Aggregated a-Synuclein Antibody	Mouse	Monoclonal	5G4	Sigma	MABN389	1:5000
Alpha-synuclein aggregate - Conformation-Specific	Rabbit	Monoclonal	MJFR-14-6-4-2	Abcam	ab209538	1:20000
Alexa Fluor™ 488, 568, 647 Secondary	-	-	-	Life Technologies	Various	1:1000
Streptavidin, HRP	-	-	-	Invitrogen	S911	1:5000
HRP	Rabbit	-	-	Life Technologies	G-21234	1:10000
HRP	Mouse	-	-	Life Technologies	G-21040	1:10000
Anti-Aggregated a-Synuclein Antibody, clone 5G4	Mouse	Monoclonal	5G4	Sigma Aldrich	MABN389	1:5000

Supplementary Table 2: Information for all primary antibodies used for immunocytochemistry and immunoblotting in this study.

ID	Age	Sex	Clinical Diagnosis	PMI (hours)	Brain weight (g)
CTRL 1	59	M	Normal	24	1234
CTRL 2	82	M	Normal	29	1362
CTRL 3	88	M	Normal	24	1243
PD 1	92	M	PD	36	1372
PD 2	76	F	PD	96	954
PD 3	81	F	PD	72	1435

Supplementary Table 3: Patient information for post-mortem brain tissue used in this study.

PMI = post-mortem interval

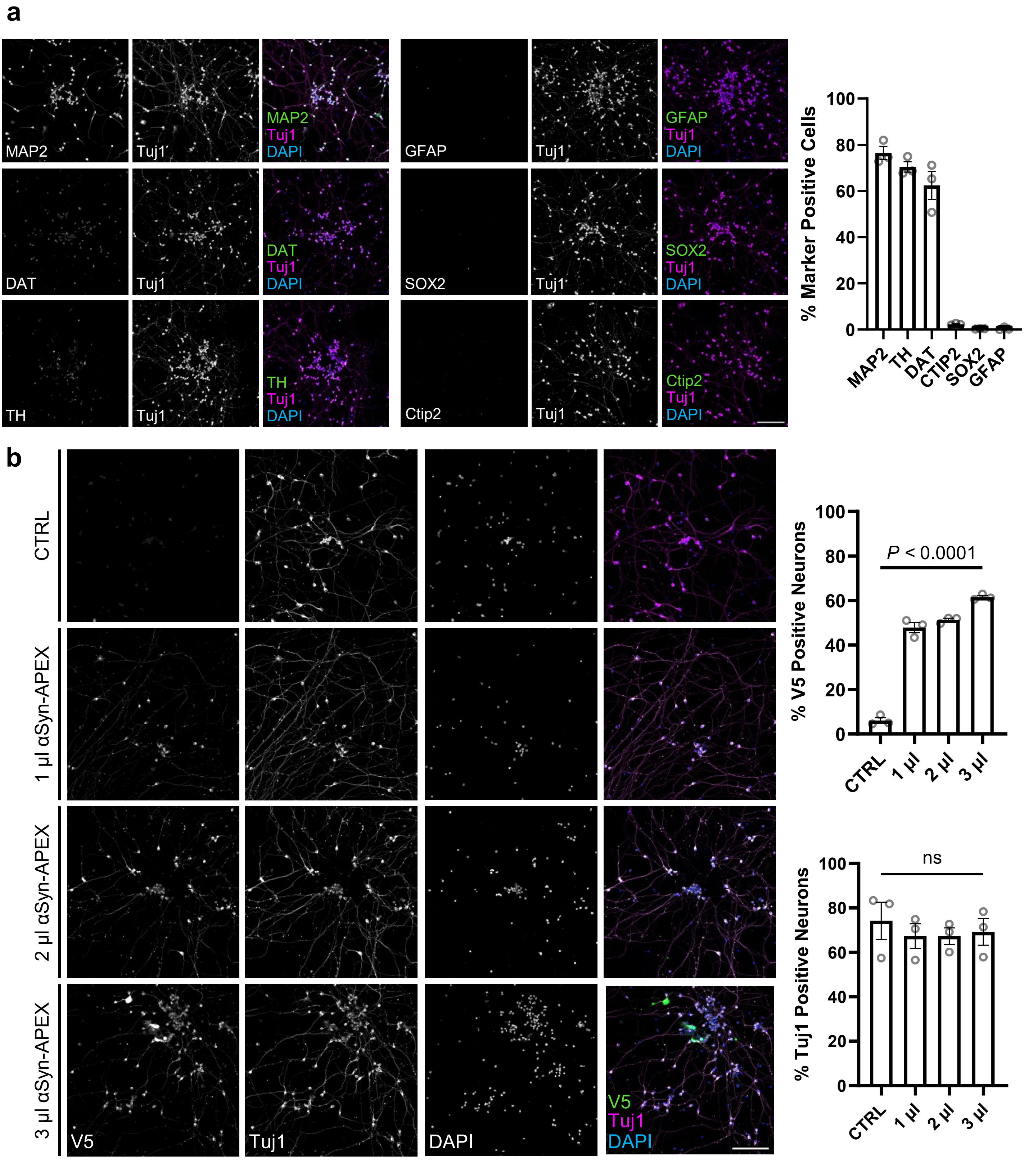

Supplementary Figure 1: αSyn-APEX was transduced into human dopaminergic neurons without impact on differentiation.

a, a, Representative images and data showing ~70-80% of differentiated cells expressed MAP2, TH, and DAT, indicating successful differentiation of hiPSCs into human dopaminergic neurons (n=3). Scale bar = 100 µm.

b, Representative images and data showing 3 µl of 7 MOI αSyn-APEX lentivirus is sufficient to transduce ~60% of hiPSC-DANs, which has no impact on the number of Tuj1-positive neurons. Ordinary one-way ANOVA – F(3,8)=291.0, p<0.0001; CTRL|3 µl αSyn-APEX: p<0.0001, n=3. Scale bar = 100 µm. Each dot corresponds to one clone differentiated once and data are mean ± SEM from at least n = 3 differentiations per clone

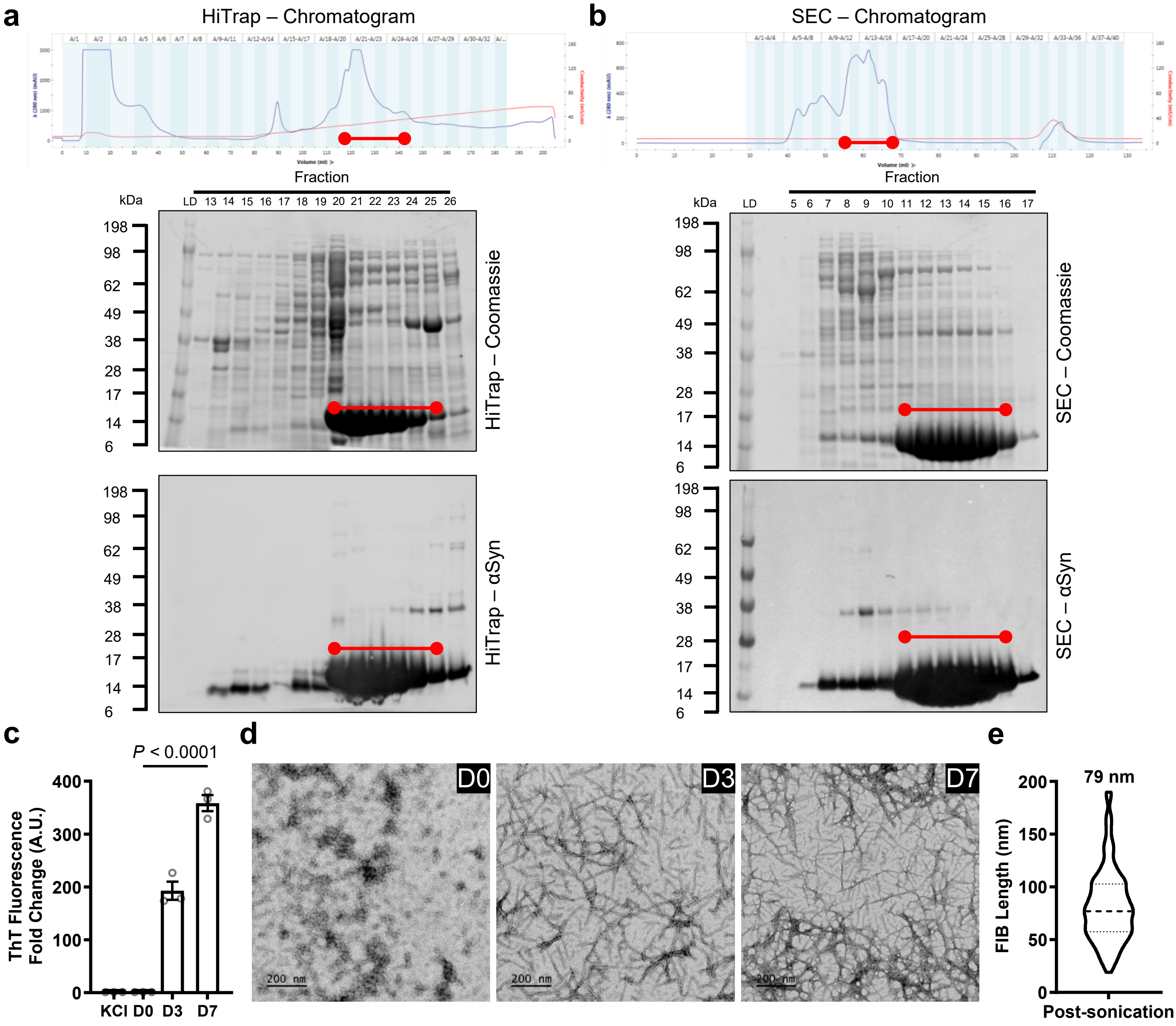

Supplementary Figure 2: αSyn fibril generation and characterisation.

a, Chromatograms, Coomassie stained gels, and αSyn stained immunoblots of HiTrap showing distinctive peaks in fractions 20-25 (HiTrap) and b, SEC peaks in 11-16 (SEC), confirming successful production of recombinant αSyn protein.

c, Thioflavin T (ThT) fluorescence intensity increases with time suggesting assembly into fibrils. Ordinary one-way ANOVA – $F(3,8)=225.8$, $p<0.0001$; D0|D7: $p<0.0001$, n=3 biological replicates. Data are presented as mean ± SEM

d, Representative TEM images showing fibrils at different time points during agitation.

e, Average length of fibrils post-sonication.

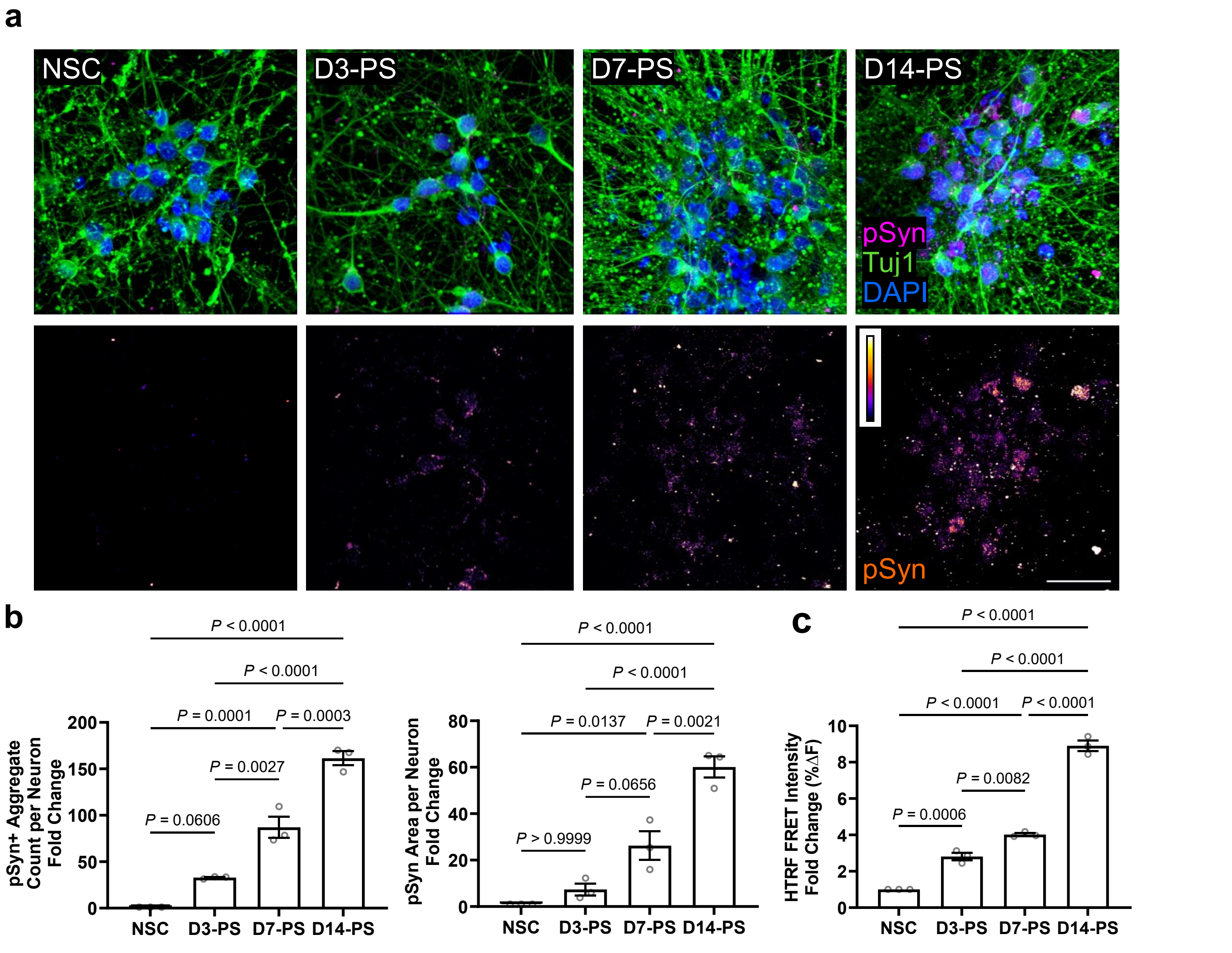

Supplementary Figure 3: Confirmation of seeding capacity of αSyn fibrils in untransduced healthy control hiPSC-DANs.

a, Representative images showing the increase in pSyn intensity at D3-PS (post-seeding), D7-PS, and D14-PS. Scale bar = 25 µm.

b, Data showing increased pSyn-positive aggregate count per neuron and aggregate area per neuron in hiPSC-DANs over time. Count: Ordinary one-way ANOVA – $F(3,8)=108.9$, $p<0.0001$; NSC|D14-PS: $p<0.0001$. Area: Ordinary one-way ANOVA – $F(3,8)=43.03$, $p<0.0001$; NSC|D14-PS: $p<0.0001$, n=3.

c, HTRF FRET data showing seeded aggregation leads to increased pSyn fluorescence intensity. HTRF: Ordinary one-way ANOVA – $F(3,8)=360.5$, $p<0.0001$; NSC|D14-PS: $p<0.0001$, n=3. Each dot corresponds to one clone differentiated once and data are mean ± SEM from at least n = 3 differentiations per clone

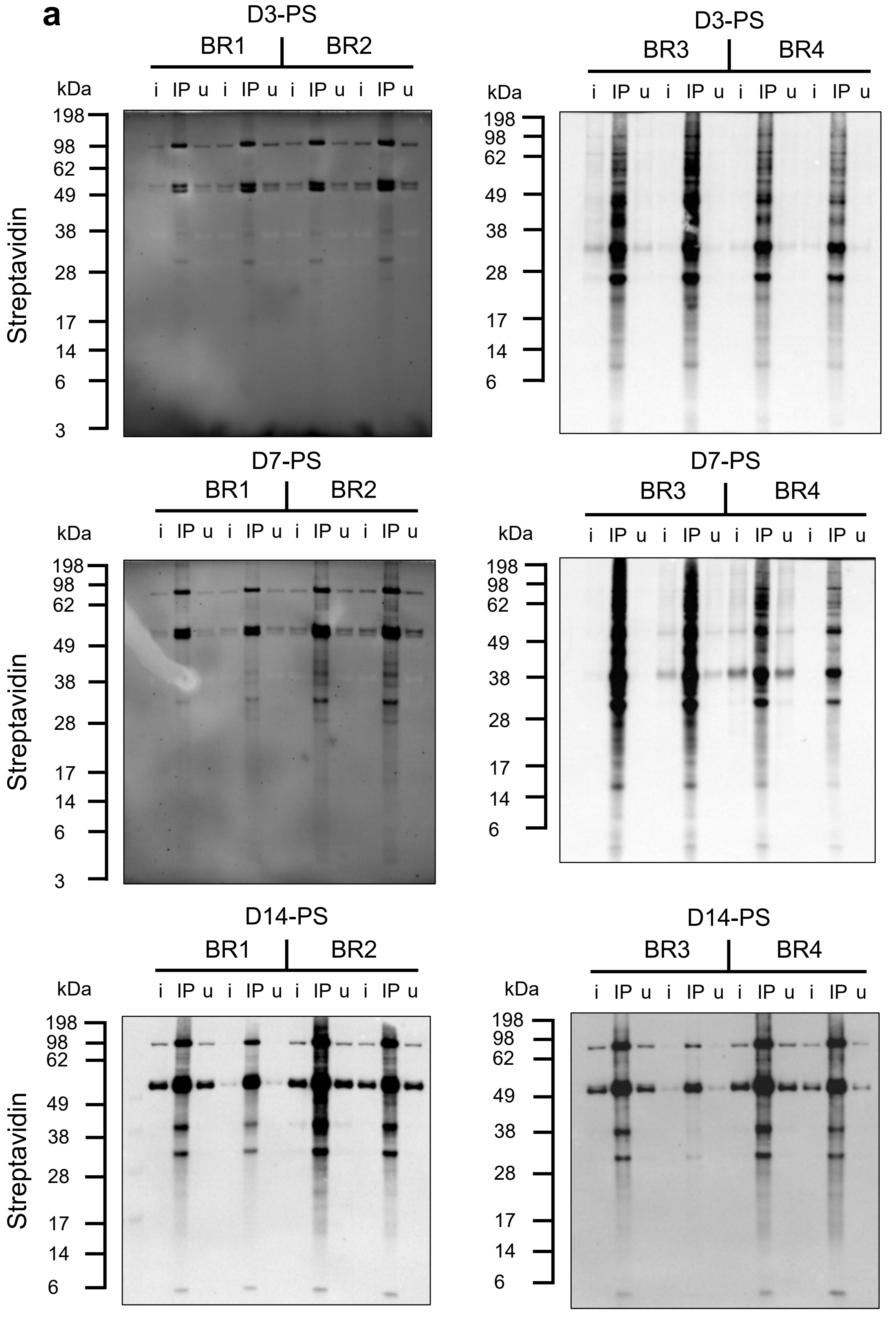

Supplementary Figure 4: Biotinylated protein immunoprecipitation.
a, All streptavidin-stained immunoblots for all biological replicates show successful immunoprecipitation at all time points prior to submission for mass spectrometry analysis. PS = post-seeding, BR = biological replicate, i = input, IP = immunoprecipitate, u = unbound.

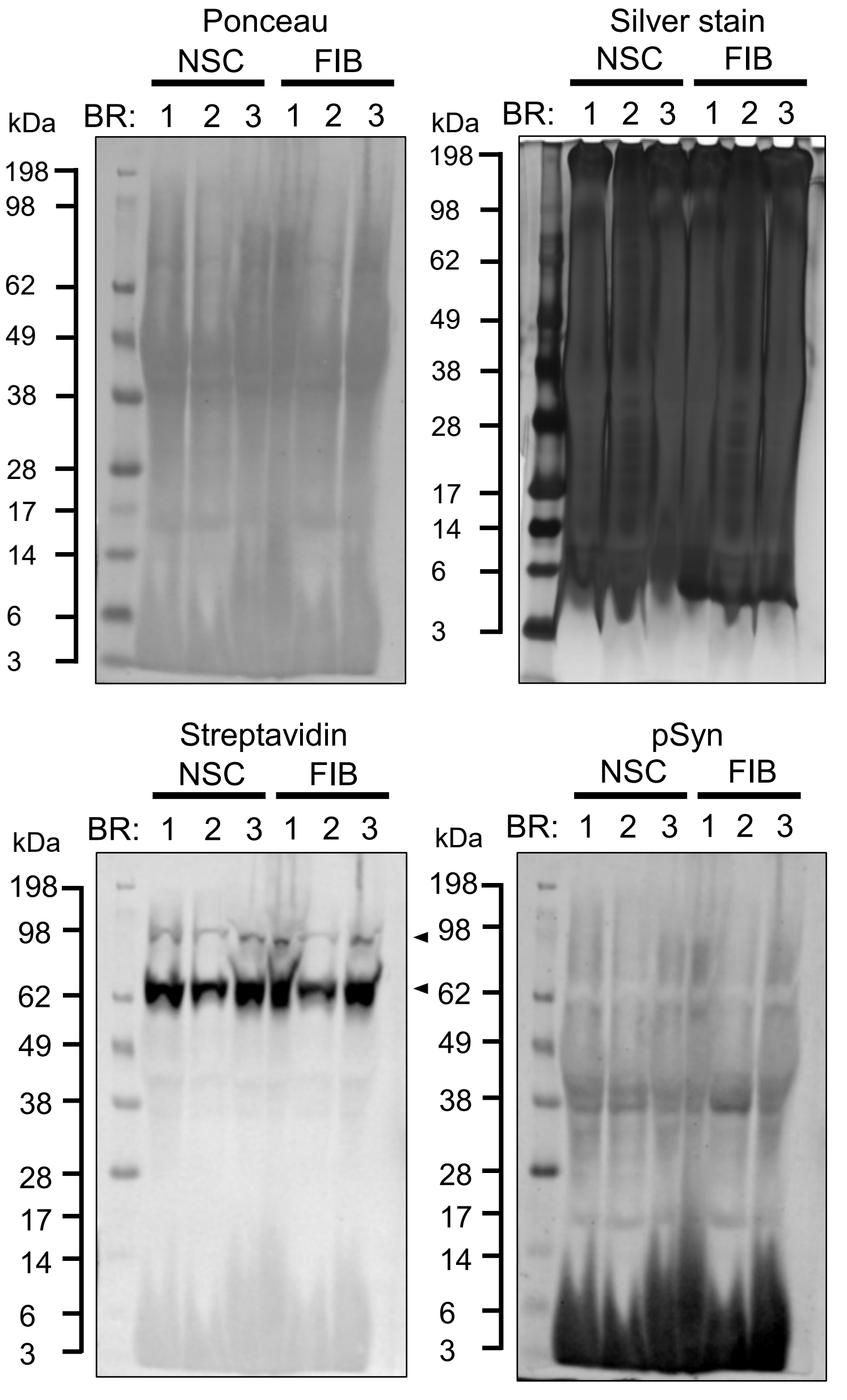

Supplementary Figure 5: No biotinylated proteins remain in pellets.

a, Ponceau-stained immunoblot, silver-stained gel, and streptavidin/pSyn-stained immunoblots demonstrating no further biotinylated proteins remaining in pellets when subjected to further lysis in 2% SDS, thus excluding partial solubilisation of aggregates as an explanation for the asymmetry observed in the D14-PS volcano plot. Immunoblot arrows indicate non-specific (ns) bands found in all hiPSC-DAN control samples probed for streptavidin – see Fig. 1f.

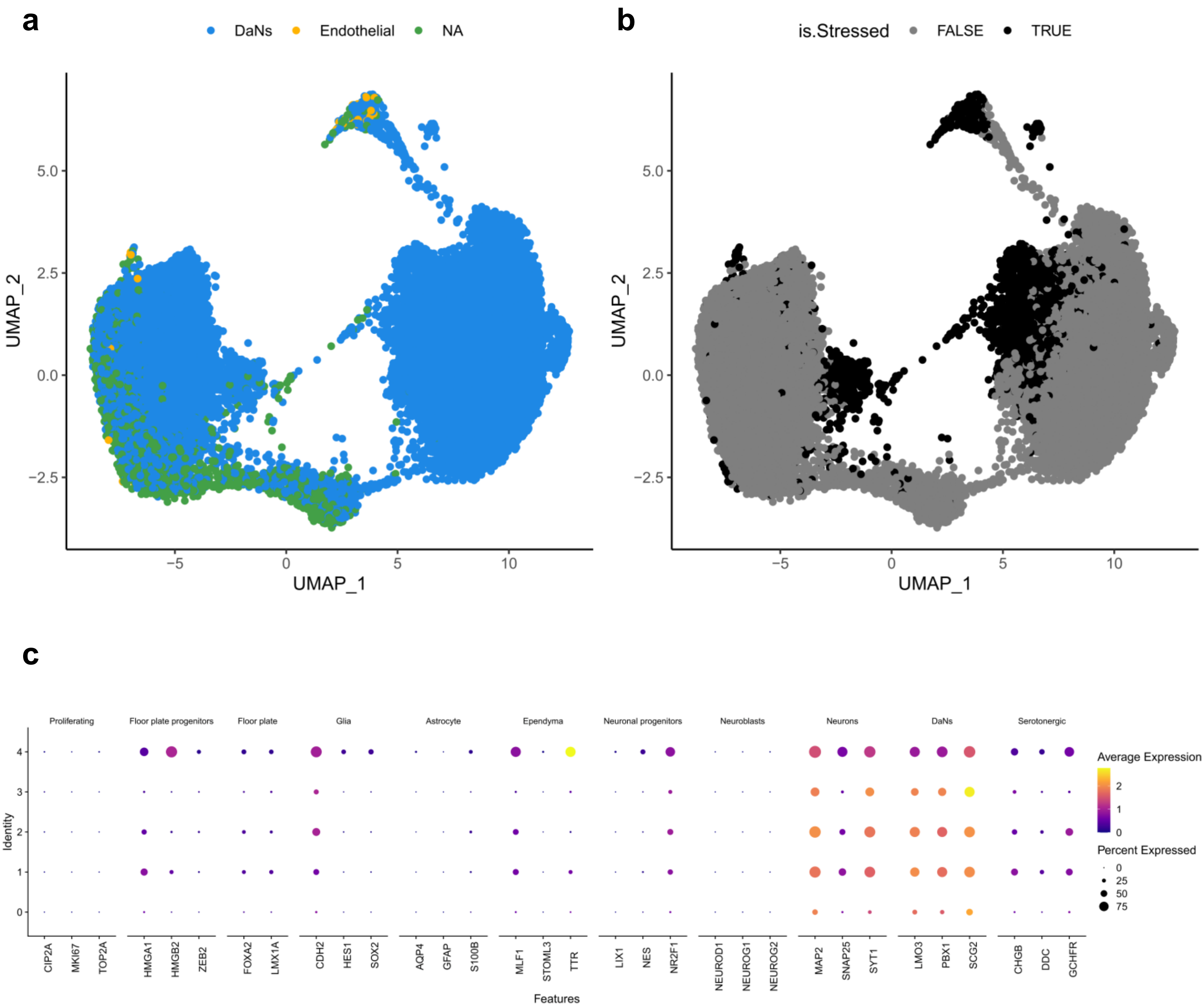

Supplementary Figure 6: Single-cell RNA-sequencing data confirming hiPSC-DAN identity and the removal of stressed hiPSC-DANs from downstream analysis.

a, UMAP showing hiPSC-DAN cell type annotations based on Welch et al. 2019.

b, UMAP showing stressed hiPSC-DANs removed from subsequent analyses.

c, Expression of cell type markers for each hiPSC-DAN cluster, confirming hiPSC-DAN identity.

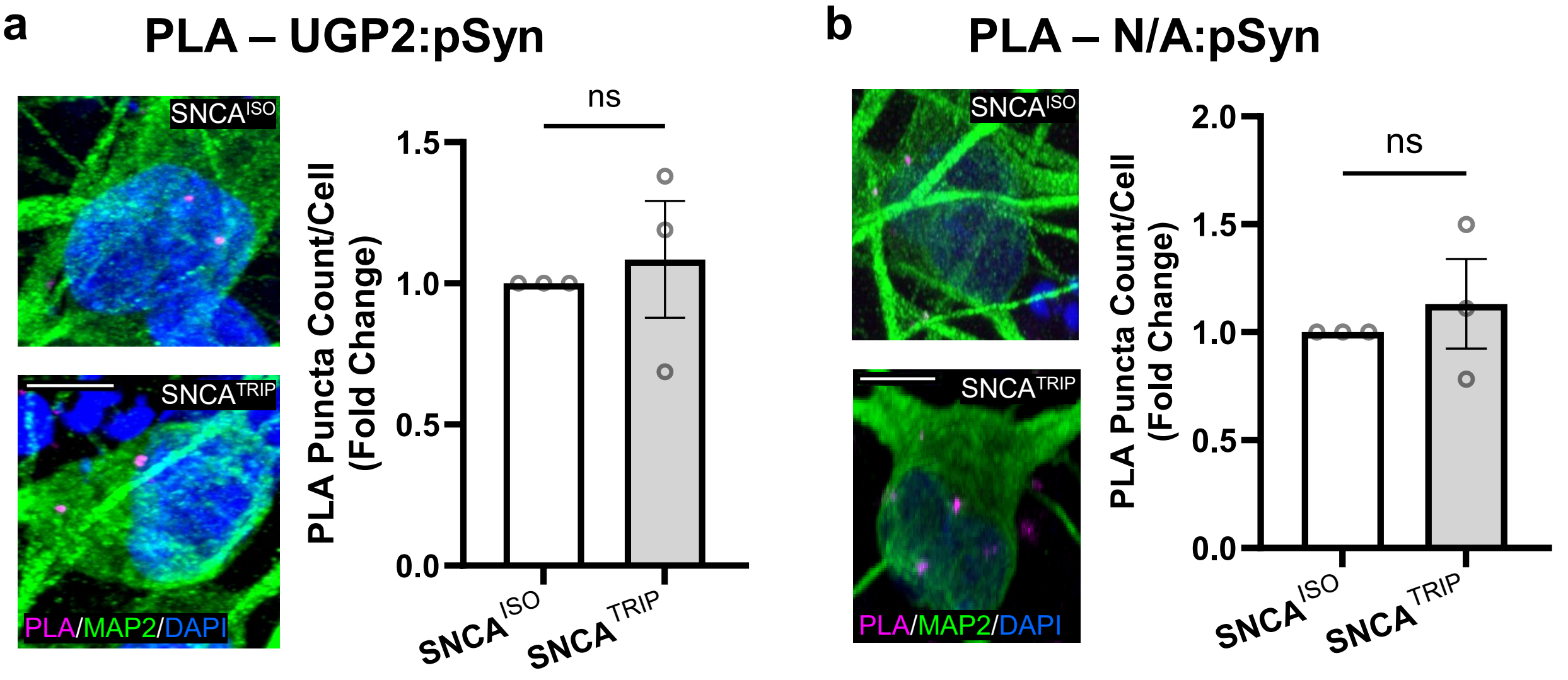

Supplementary Figure 7: Controls for proximity ligation assay (PLA) showing minimal off-target binding between PLA probes.

a, PLA for UGP2 and pSyn in SNCAISO and SNCATRIP hiPSC-DANs . Two-sided Unpaired t-test – t(4)=0.4114, p=0.7019, n=3. Scale bar = 5 µm.

b, PLA for no primary antibody control and pSyn showing limited amplification in SNCAISO and SNCATRIP hiPSC-DANs; Two-sided Unpaired t-test – t(4)=0.6330, p=0.5611, n=3. Scale bar = 5 µm. Each dot corresponds to one clone differentiated once and data are mean±SEM from at least n = 3 differentiations per clone

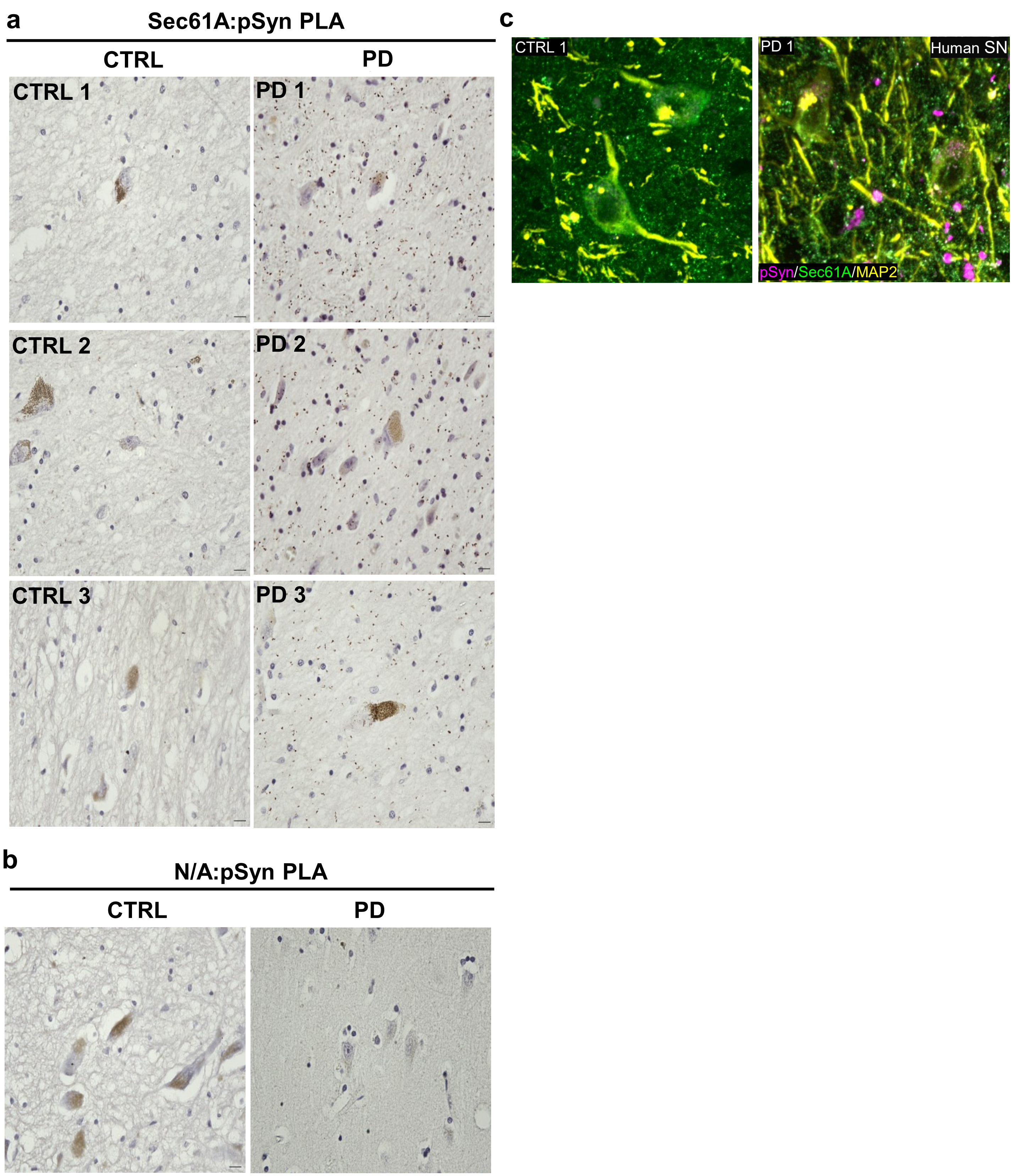

Supplementary Figure 8: Sec61A:pSyn interaction in postmortem human substantia nigra tissue.
a, Sec61A:pSyn PLA for three healthy controls (CTRL) and three patients with Parkinson's disease (PD) – see **Fig. 4f**. Scale bar = 13 µm
b, PLA for no primary antibody control and pSyn showing no amplification in CTRL and PD patient human brain tissue. Scale bar = 13 µm.
c, Representative immunofluorescence staining of Sec61A, pSyn, and MAP2 in human substantia nigra sections from a healthy control (CTRL) and a PD patient.

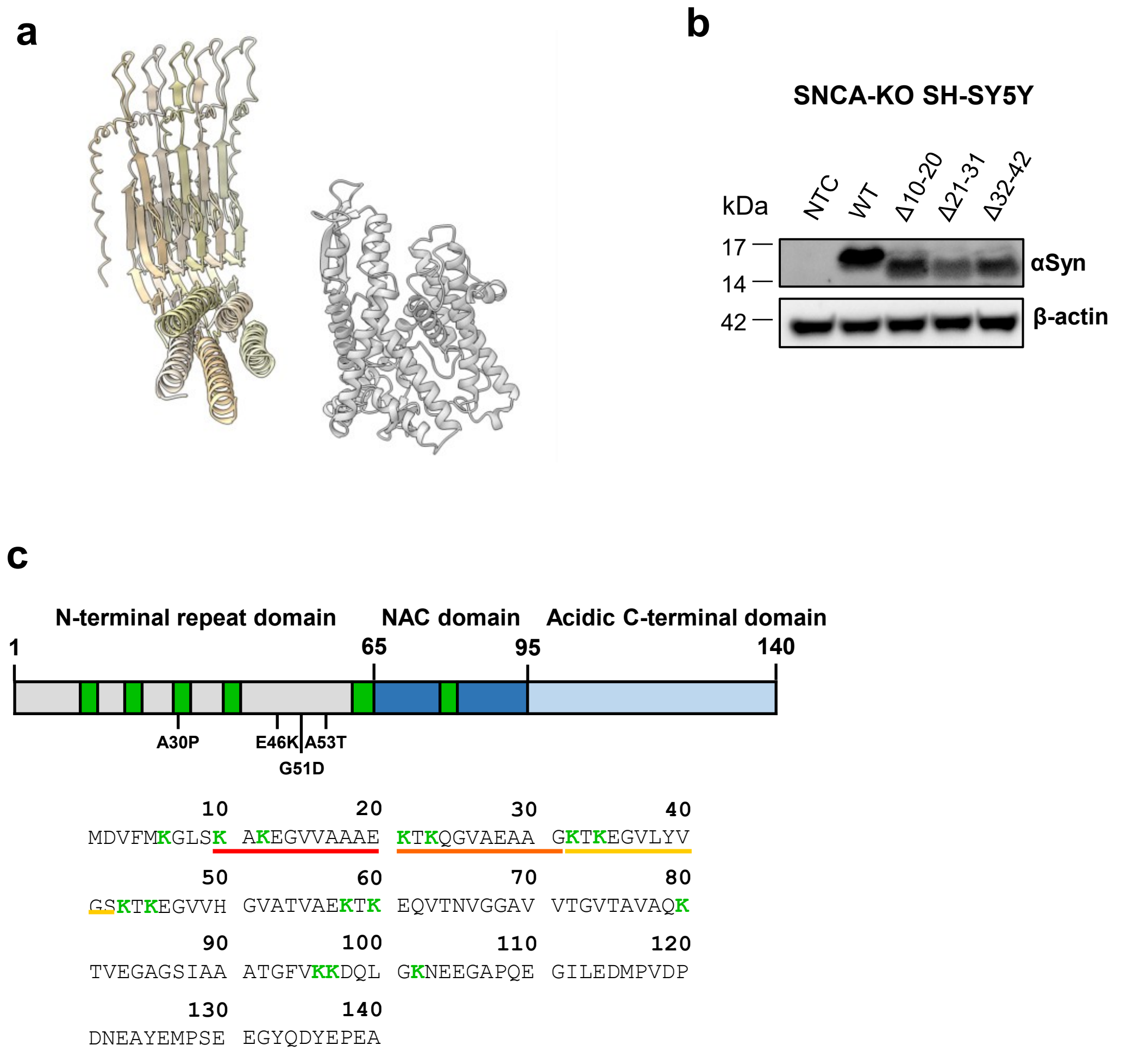

Supplementary Figure 9: Modelling pSyn:Sec61A interaction

a, AlphaFold 3 predicts that αSyn assemblies do not interact with Sec61A.

b, Immunoblots showing expression of all αSyn constructs in SY-5H cells knockout for endogenous αSyn

c, Diagram of αSyn domains and amino acid sequence highlighting known mutations, tandem repeat regions (green – schematic), lysine residues (green – sequence), and the deletion mutations (red, orange, yellow).

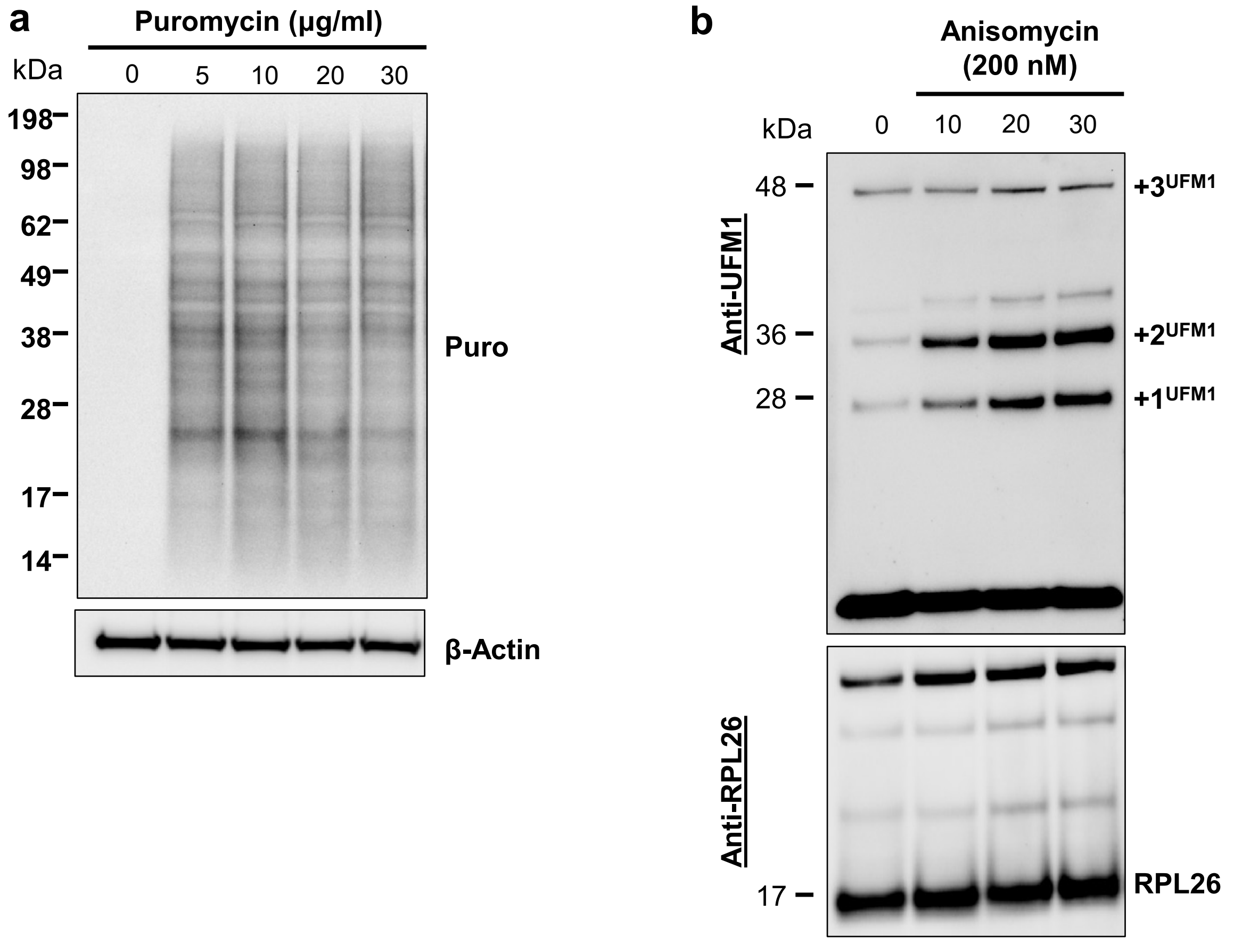

Supplementary Figure 10: Puromycilation and UFMylation.
a, Representative immunoblot demonstrating successful puromycin integration into nascent polypeptide chains at varying concentrations.
b, Representative immunoblot showing increased UFMylation occurs over time with Anisomycin treatment.

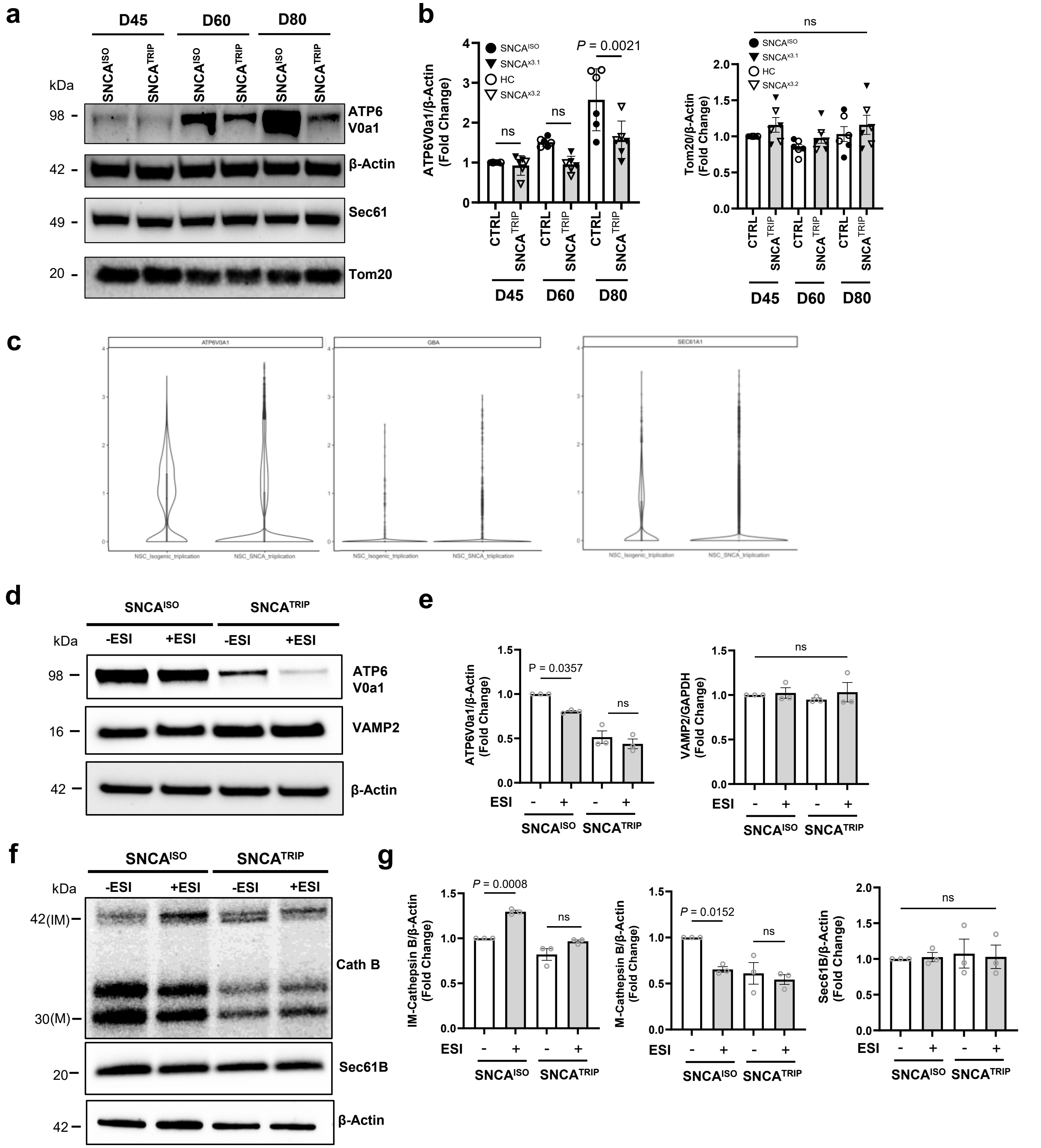

Supplementary Figure 11: Defective cotranslational translocation impairs delivery of organelle-associated proteins in SNCA^{TRIP} DANs and is phenocopied by Eeyarestatin I treatment.

a, Representative immunoblot and quantification in **b**, demonstrating a reduction of v-ATPase subunit ATP6V0a1 levels over time in SNCA^{TRIP} hiPSC-DANs compared to SNCA^{ISO} controls at all time points. Tom20, which is not a translocon substrate, remains unchanged. Ordinary one-way ANOVA – F(5,29)=7.976, p<0.0001, D80 CTRL|SNCA^{TRIP}: p<0.0021, n=6.**c,** mRNA transcript counts from scRNA-seq analysis for Sec61A and PD-related translocon substrates (ATP6V0A1, GBA) are similar in SNCA^{TRIP} DANs compared to SNCA^{ISO} DANs. **d,** Representative immunoblot and quantification in **e**, demonstrating a reduction of v-ATPase subunit ATP6V0a1 levels with Eeyarestatin I (ESI) treatment; VAMP2, which is a GET pathway substrate, remains unchanged. Ordinary one-way ANOVA – F(3,8)=33.69, p<0.0001, ATP6V0a1 D80 SNCA^{ISO}|SNCA$^{ISO\ +ESI:}$ p<0.0357, n=3.**f,** Representative immunoblot and quantification in **g**, demonstrating a reduction of Mature Cathepsin B and an accumulation of Immature (IM) Cathepsin B treatment with Eeyarestatin I (ESI) treatment. Sec61B, which is a GET pathway substrate, remains unchanged at Day80. Ordinary one-way ANOVA – F(3,8)=5.718, p=0.0217, Mature(M) Cathepsin B SNCAI^{SO}|SNCA$^{ISO\ +ESI}$: p<0.0359. Ordinary one-way ANOVA – F(3,8)=33.25, p<0.0001, Immature (IM) Cathepsin B D80 SNCA^{ISO}|SNCA$^{ISO\ +ESI}$: p<0.0008, n=3. Each dot corresponds to one clone differentiated once and data are mean ± SEM from at least n = 3 differentiations per clone

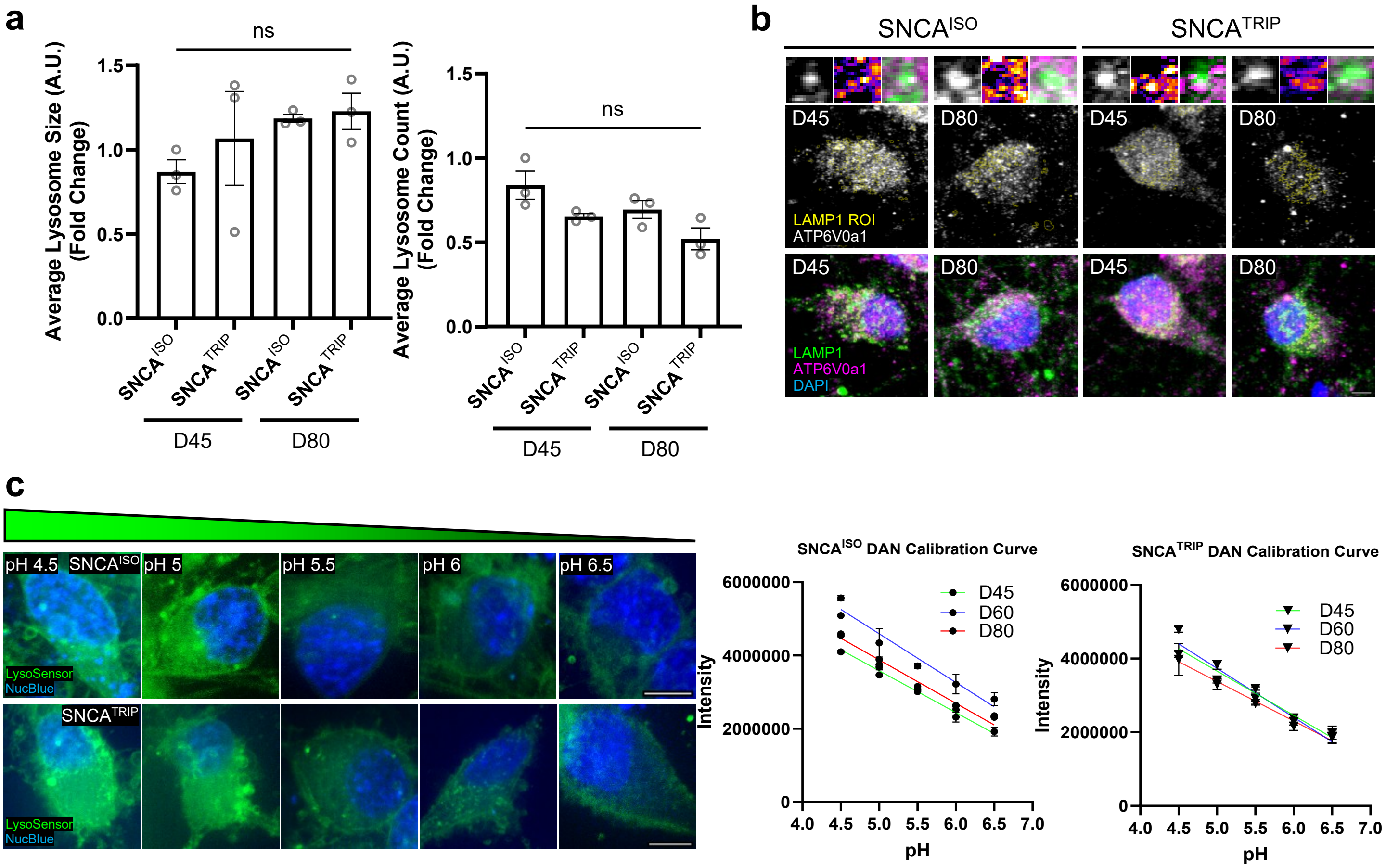

Supplementary Figure 12: Assessment of lysosomal size and acidification.

a, Data showing no difference in lysosome size or number between SNCA^{TRIP} and SNCA^{ISO} hiPSC-DANs over time.

b, Representative images showing ATP6V0a1 does not reach lysosomes at D80 in SNCA^{TRIP} hiPSC-DANs based on thresholded LAMP1 regions of interest (ROI) generated in ImageJ.

c, Representative images and quantification showing ratiometric intensity calibration curves for iPSC-derived SNCA^{TRIP} and SNCA^{ISO} DANs used for quantification of lysosomal pH at each time point.

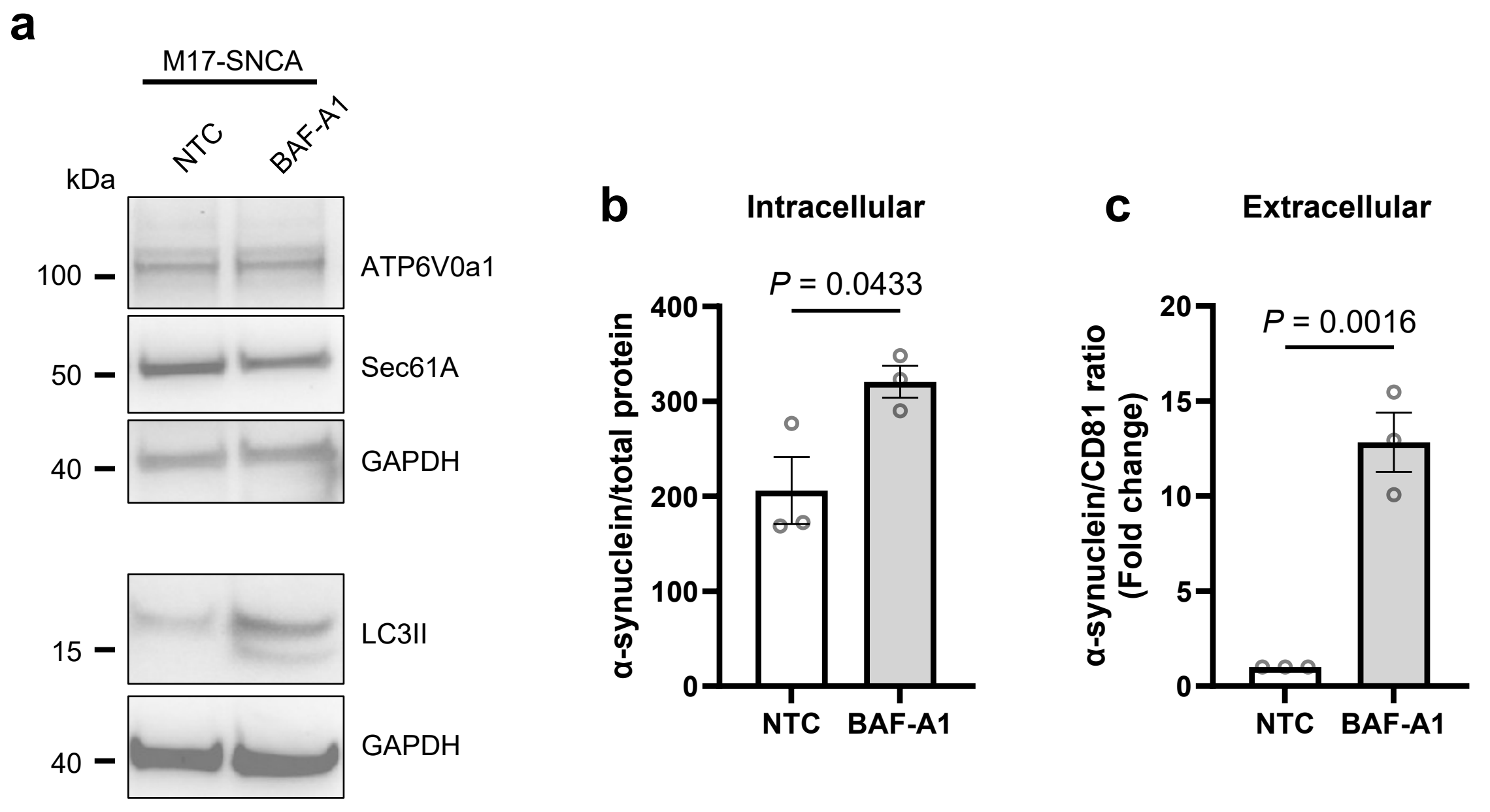

Supplementary Figure 13. Effect of v-ATPase inhibition on intracellular levels and extracellular vesicle (EV) release of α-synuclein.

a, Representative immunoblots of ATP6V0a1, Sec61A and LC3B demonstrating increased LC3II in M17 neuroblastoma cells stably overexpressing α-synuclein after treatment with bafilomycin (BAF-A1).

b, Intracellular α-synuclein levels as measured by MSD electrochemiluminescence are increased after treatment with BAF-A1. Data corrected to total protein levels in the cell lysates. NTC|+BAF-A1: Two-sided Unpaired t-test – $t(4)=2.919$, $p=0.0433$, n=3 biological replicates. Data presented as mean $\pm$ SEM.

c, Inhibition of v-ATPase using BAF-A1 increased the release of αSyn in EVs. NTC|+BAF-A1: Two-sided Unpaired t-test – $t(4)=7.584$, $p=0.0016$. n=3 biological replicates. Data presented as mean $\pm$ SEM.

a

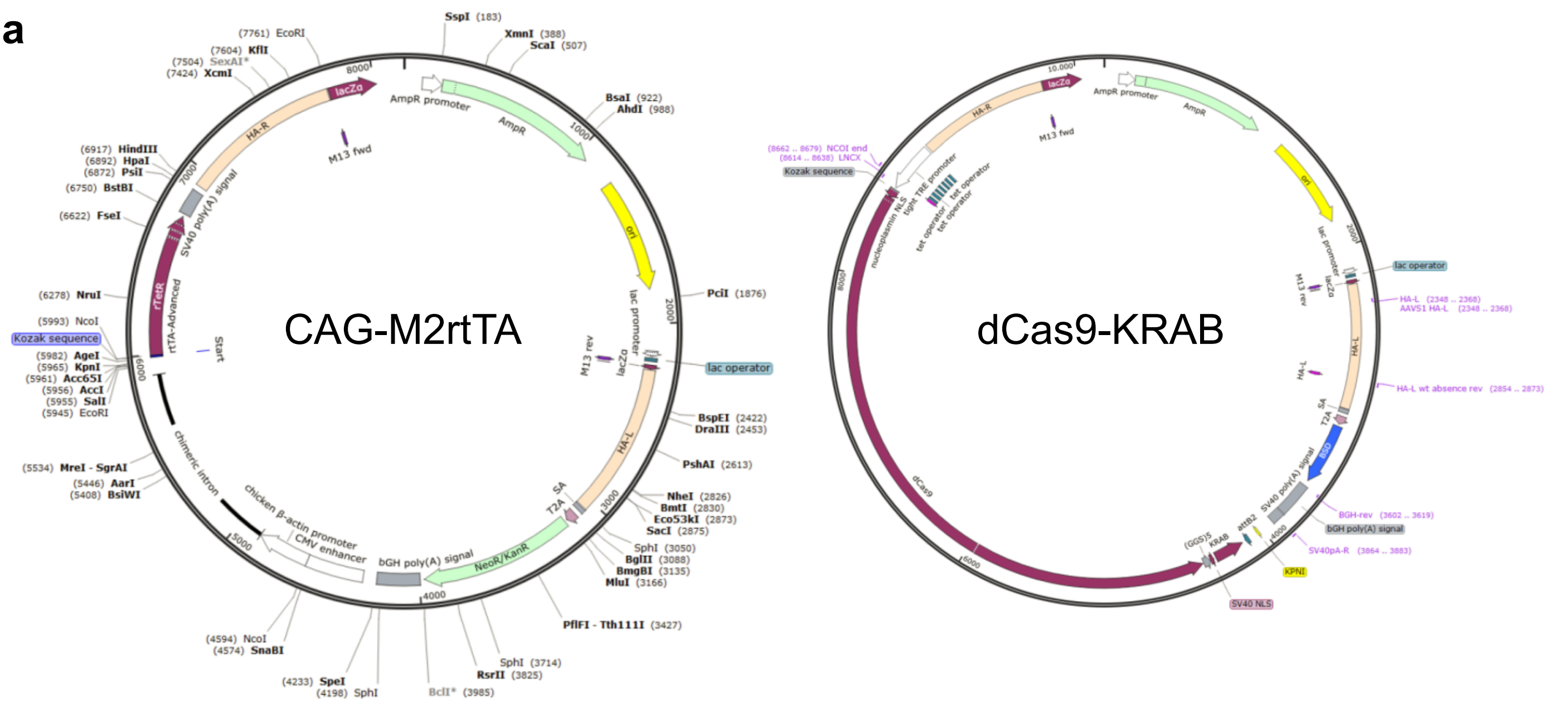

b

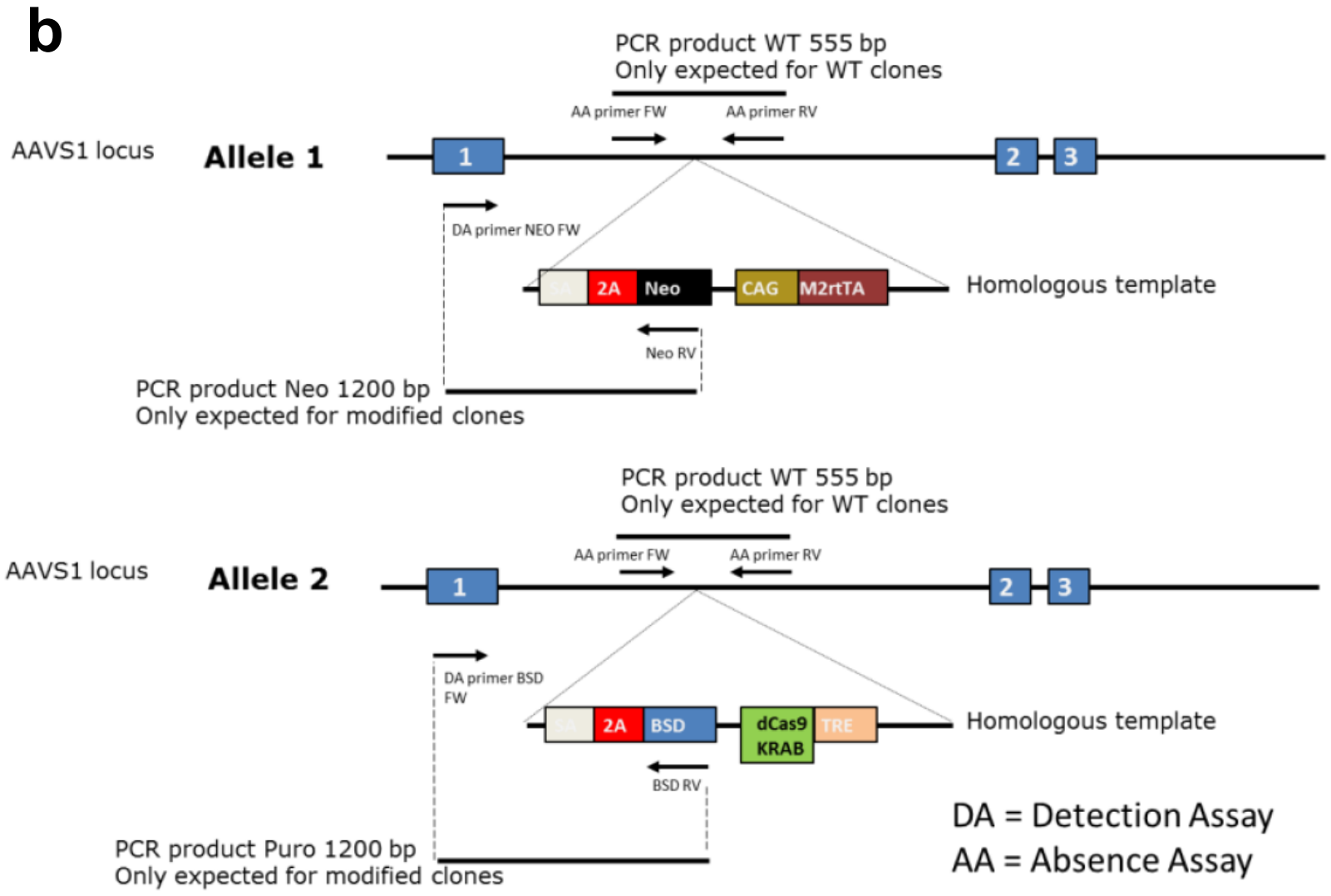

c

CRISPR	Sequence
CR2 AAVS1_rv	TGTCCCTAGTGGCCCCACTGTGG

Primer	Sequence	Product size
DA primer BSD_fw	CTCCGACGGATGTC TCCCTTGCG	1200
DA primer BSD_rv	CACGCTGTAATCCTC GCTGA	
DA primer NEO_fw	CCAGAGGCGGCGCA GAAGCCAG	1200
DA primer NEO_rv	ATCTTGTTCAATGGC CGATCCCAT	
AA primer_fw	GTCACCAGATAAGG ATCTGCC	555
AA primer_rv	CTCCGACGGATGTC TCCCTTGCG	

d

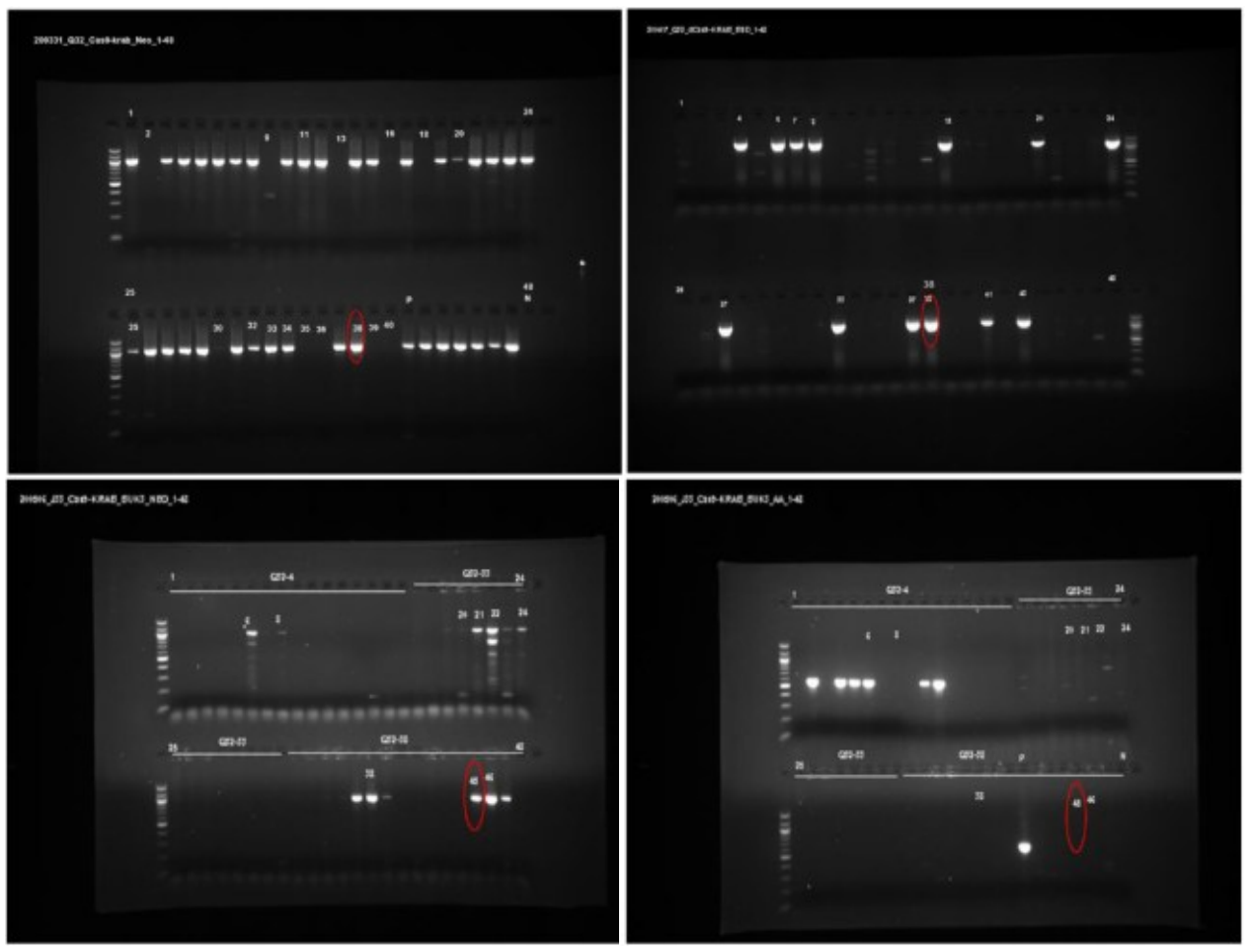

Supplementary Figure 14. Generation of doxycycline inducible dCas9-KRAB gene in SNCA^{TRIP} hiPSCs.

a, Plasmid maps of the reverse tetracycline-controlled transactivator (rtTA) and dCas9 sequences used to generate dead Cas9-KRAB hiPSCs. Dead Cas9-KRAB (dCas9-KRAB) cell line was generated by including a reverse tetracycline-controlled transactivator mutant 2 (M2rtTA) on one allele of the AAVS1 locus (left) and the dCas9-KRAB expression cassette on the other allele of the AAVS1 locus (right). The plasmid used for integration of the CAG promoter regulated M2rtTA contains a resistance gene against neomycin (NeoR). Expression of dCas9-KRAB is driven by a minimal CMV promoter including a TRE (tetracycline transactivator responsive element). SNCA^{TRIP} iPSCs were nucleofected with the CRISPR "CR2_AAVS1_RV" and 2 µg of each plasmid.

b, Schematic showing the integration sites of the rtTA and dCas9 sequences at the AAVS1 loci. 1, 2 and 3 represent the respective exon numbers in the AAVS1 locus. SA: splice acceptor, 2A: self-splicing protein, CAG: constitutively active promoter, TRE: tetracycline transactivator responsive element. Screening primers are shown as arrows. Neomycin selection was applied (250 ng/mL). After neomycin selection for 4 days, several clones were found and analysed by PCR using the following primer pairs: Insertion of the Blasticidin (BSD) resistance gene ("DA primer BSD FW", "BSD RV"), insertion of the Neo resistance gene ("DA primer NEO FW", "NEO RV"), and amplification of the unwanted empty locus ("AA primer FW", "AA primer RV").

c, Tables showing the primer sequences used to generate and confirm the doxycycline inducible dCas9 system in hiPSCs.

d, PCR gels confirming insertion of the M2rtTA and the dCas9-KRAB expression cassettes into the AAVS1 locus of SNCA^{TRIP} hiPSCs resulting in a doxycycline inducible dCas9 system.

a

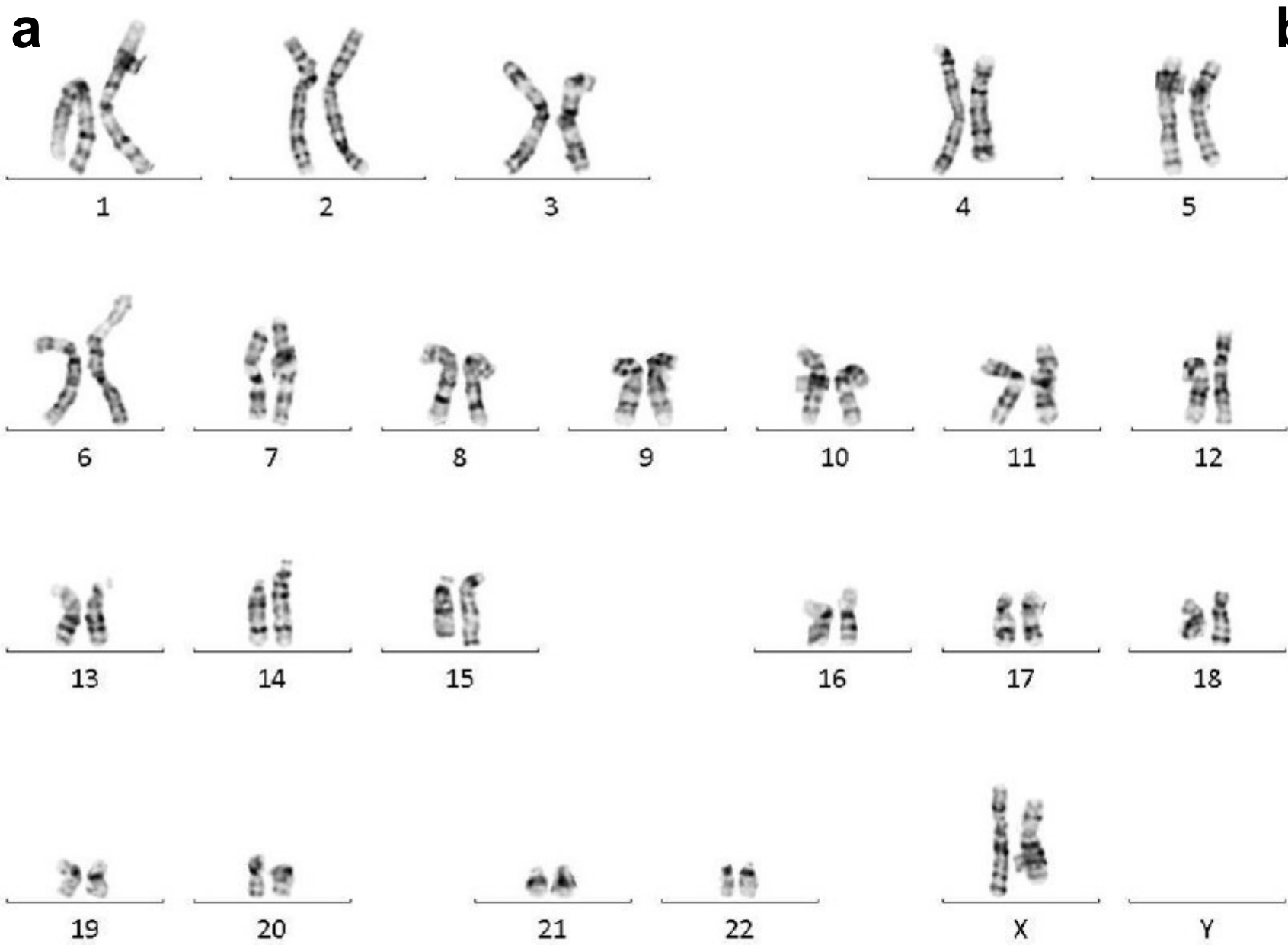

b

	$SNCA^{TRIP}$		$SNCA^{TRIP}$ + dCas9-KRAB	
**Marker**	**Allele 1**	**Allele 2**	**Allele 1**	**Allele 2**
AMEL	X		X	
CSF1PO	12		12	
D13S317	9	11	9	11
D16S539	11	12	11	12
D18S51	13	22	13	22
D19S433	14		14	
D21S11	30	31,2	30	31,2
D2S1338	21	23	21	23
D3S1358	16	17	16	17
D5S818	11		11	
D7S820	9	11	9	11
D8S1179	13	14	13	14
FGA	20	23	20	23
TH01	7	9,3	7	9,3
TPOX	8	11	8	11
vWA	15	18	15	18

c

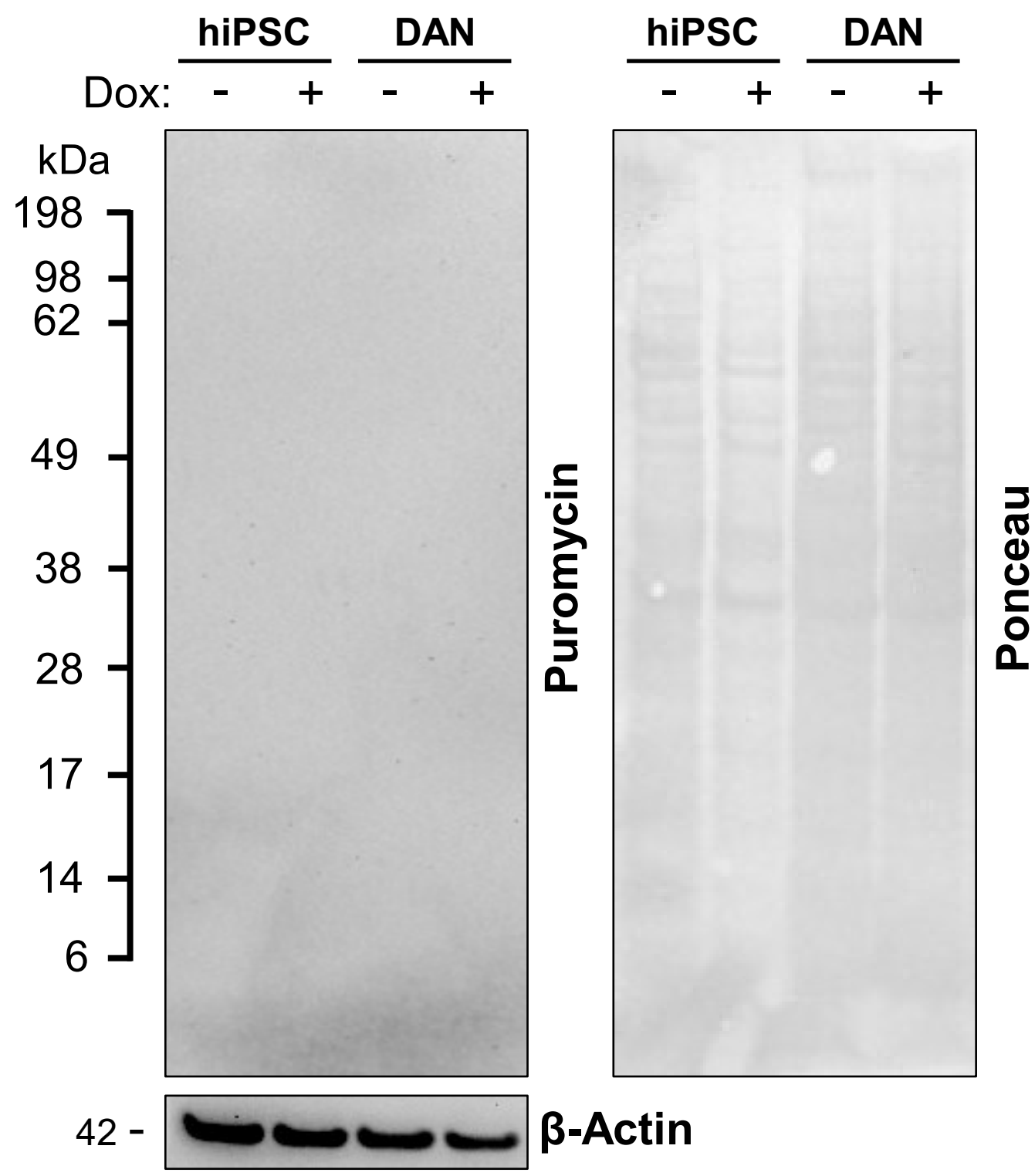

Supplementary Figure 15. Karyotyping and confirmation of parent cell lineage.

a, Karyotyping of the newly generated dCas9-KRAB $SNCA^{TRIP}$ hiPSCs.

b, Short tandem repeat (STR) analysis of the dCas9-KRAB $SNCA^{TRIP}$ hiPSC clone and its corresponding line of origin. The number of allelic repeats tested in each locus matched between both lines, confirming that $SNCA^{TRIP}$/dCas9 clone was derived from the parent $SNCA^{TRIP}$ hiPSC line.

c, Representative immunoblots showing puromycin used for initial sgRNA expression selection does not persist in cultured hiPSCs or hiPSC-DANs.

Source Data for Supplementary Figures (Uncropped image)

Fig S2A

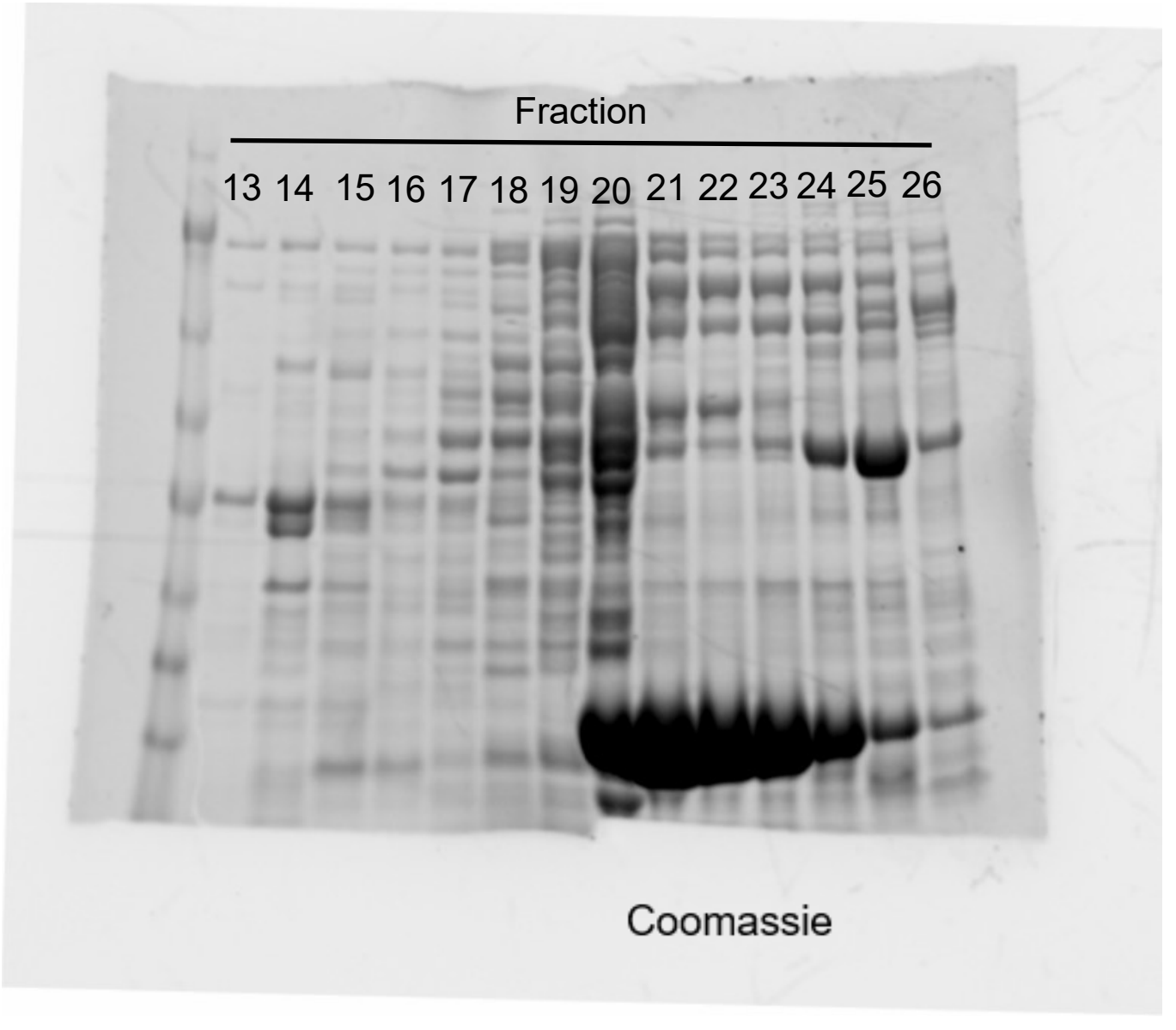

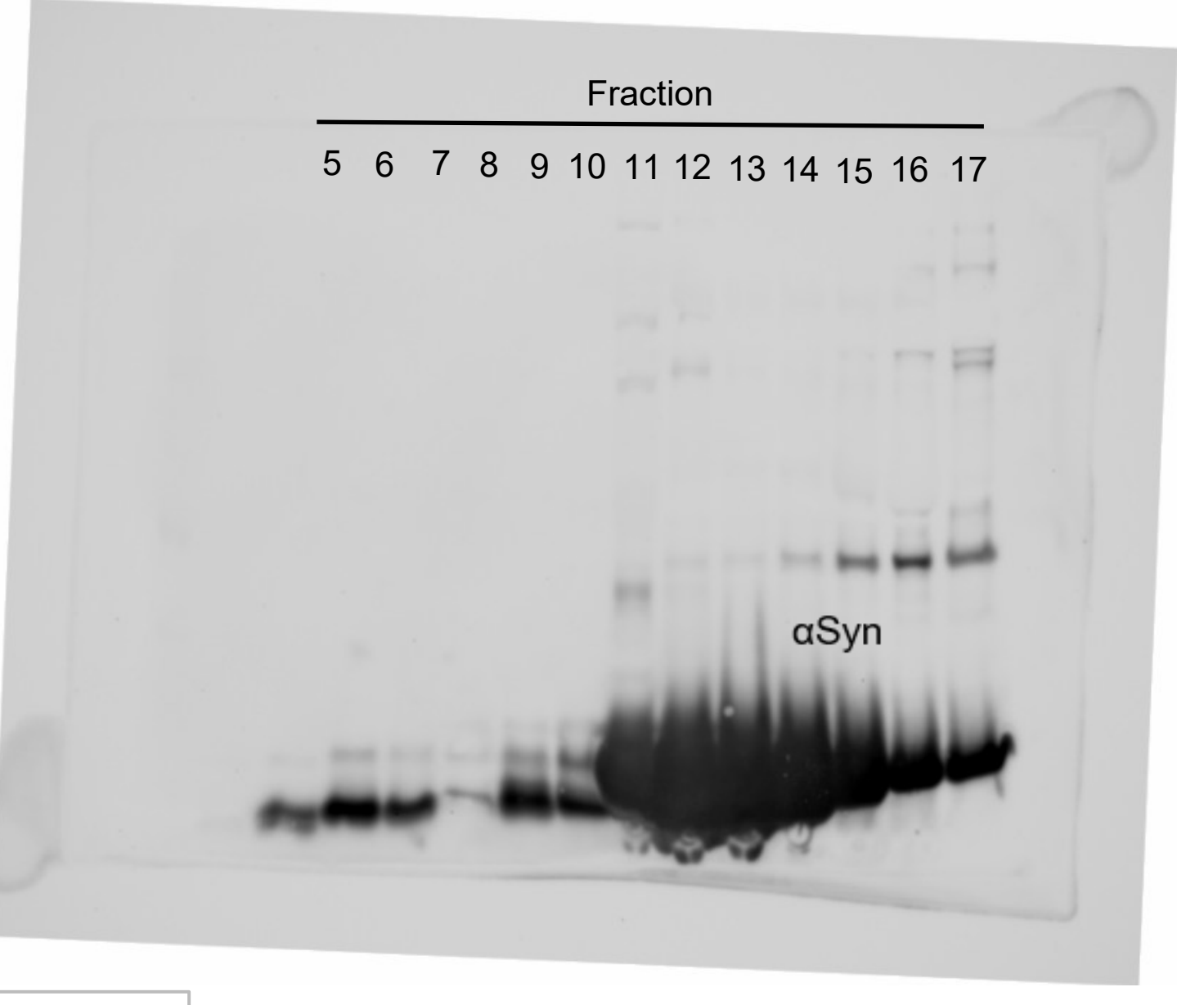

Fig S2B

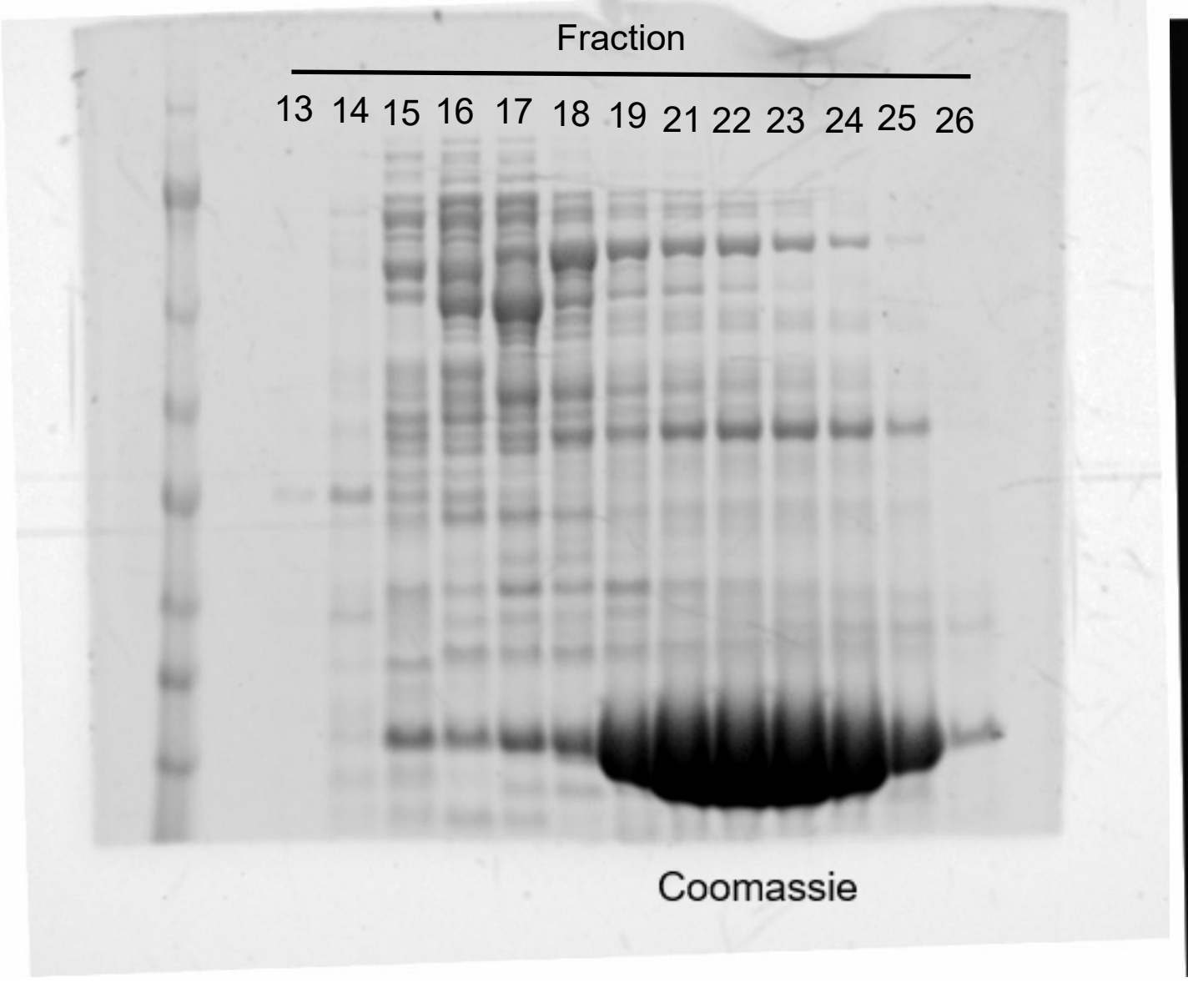

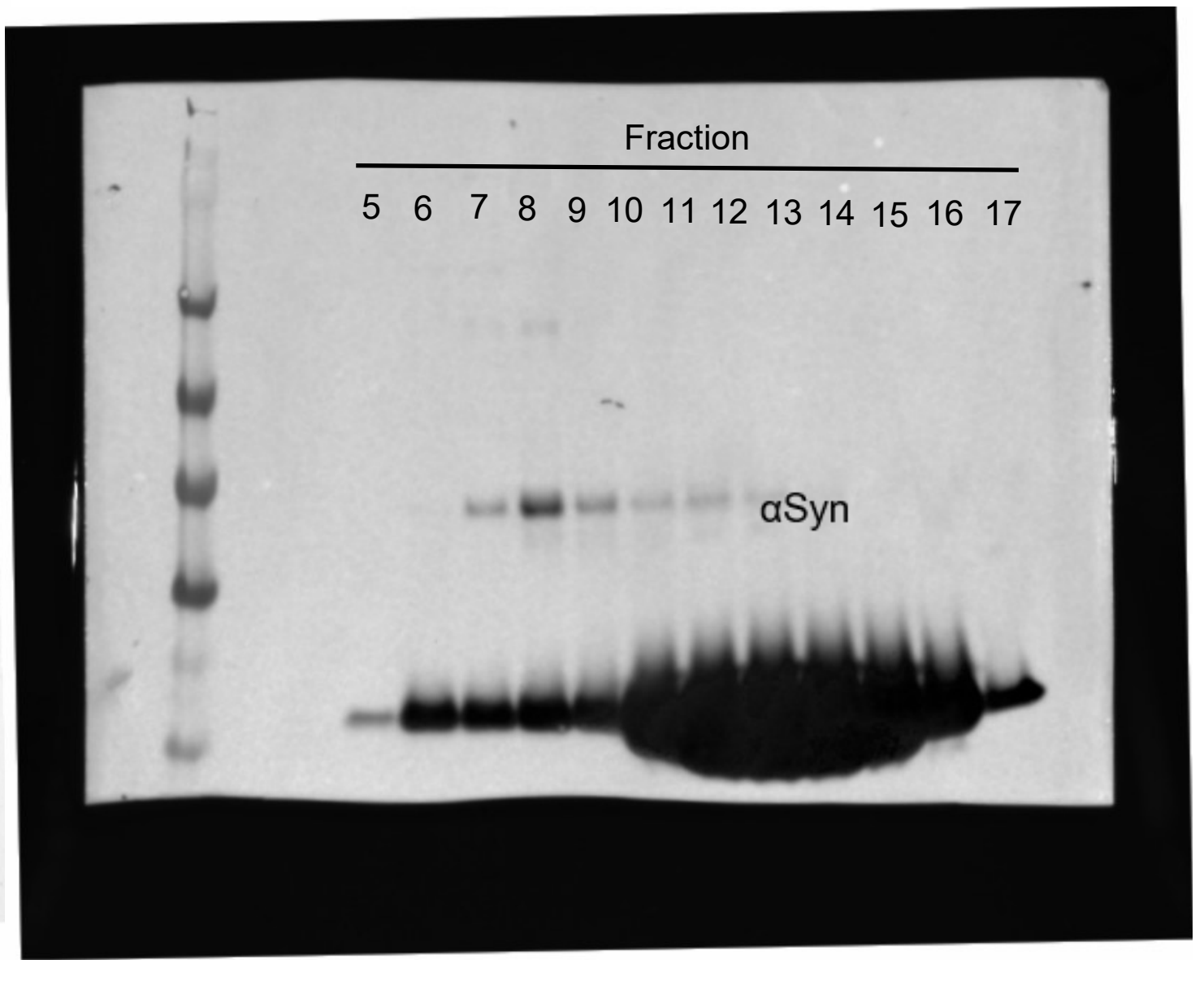

Fig S4

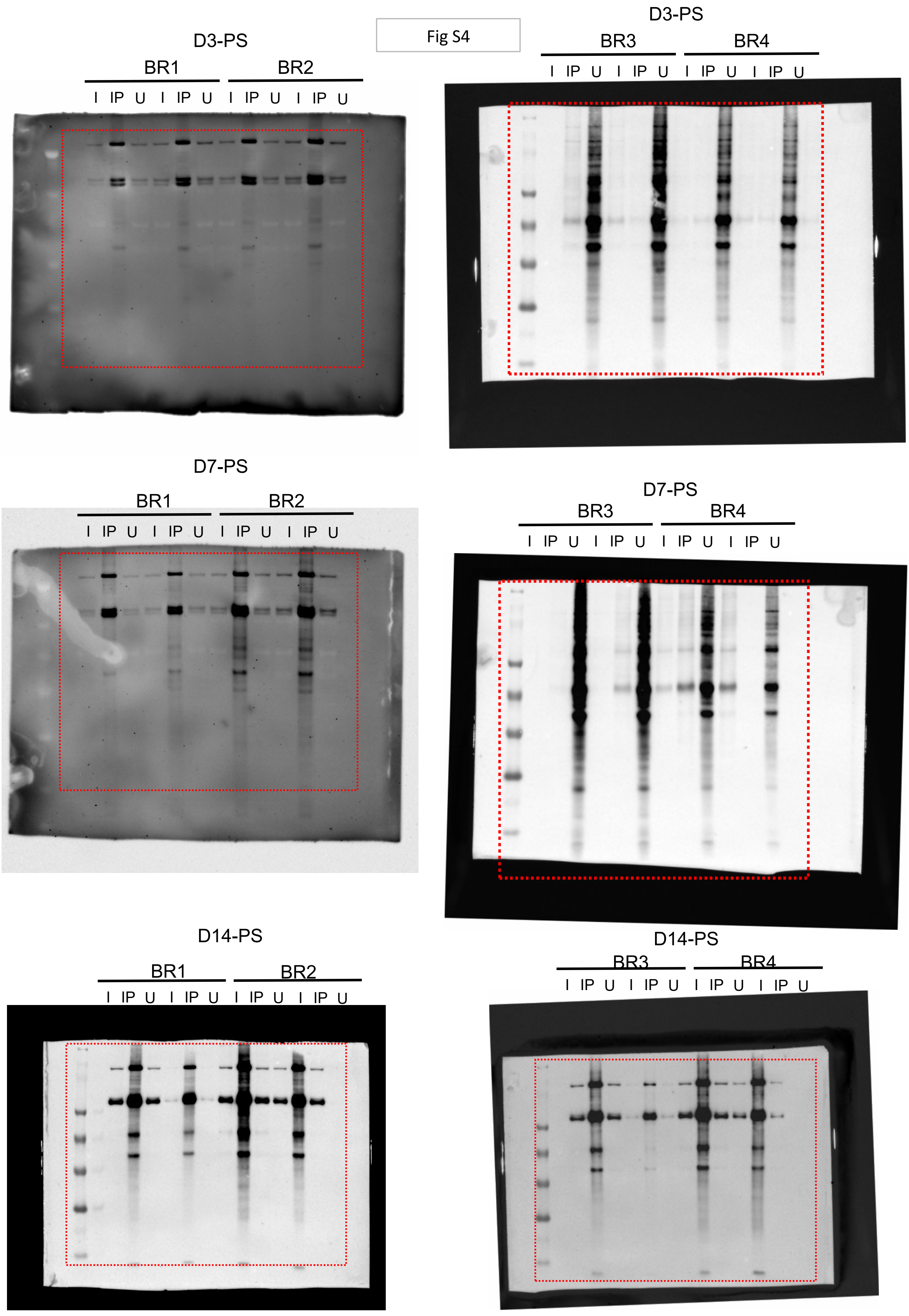
D3-PS
BR1
BR2
I IP U I IP U I IP U I IP U
D3-PS
BR3
BR4
I IP U I IP U I IP U I IP U
D7-PS
BR1
BR2
I IP U I IP U I IP U I IP U
D7-PS
BR3
BR4
I IP U I IP U I IP U I IP U
D14-PS
BR1
BR2
I IP U I IP U I IP U I IP U
D14-PS
BR3
BR4
I IP U I IP U I IP U I IP U

Fig S5

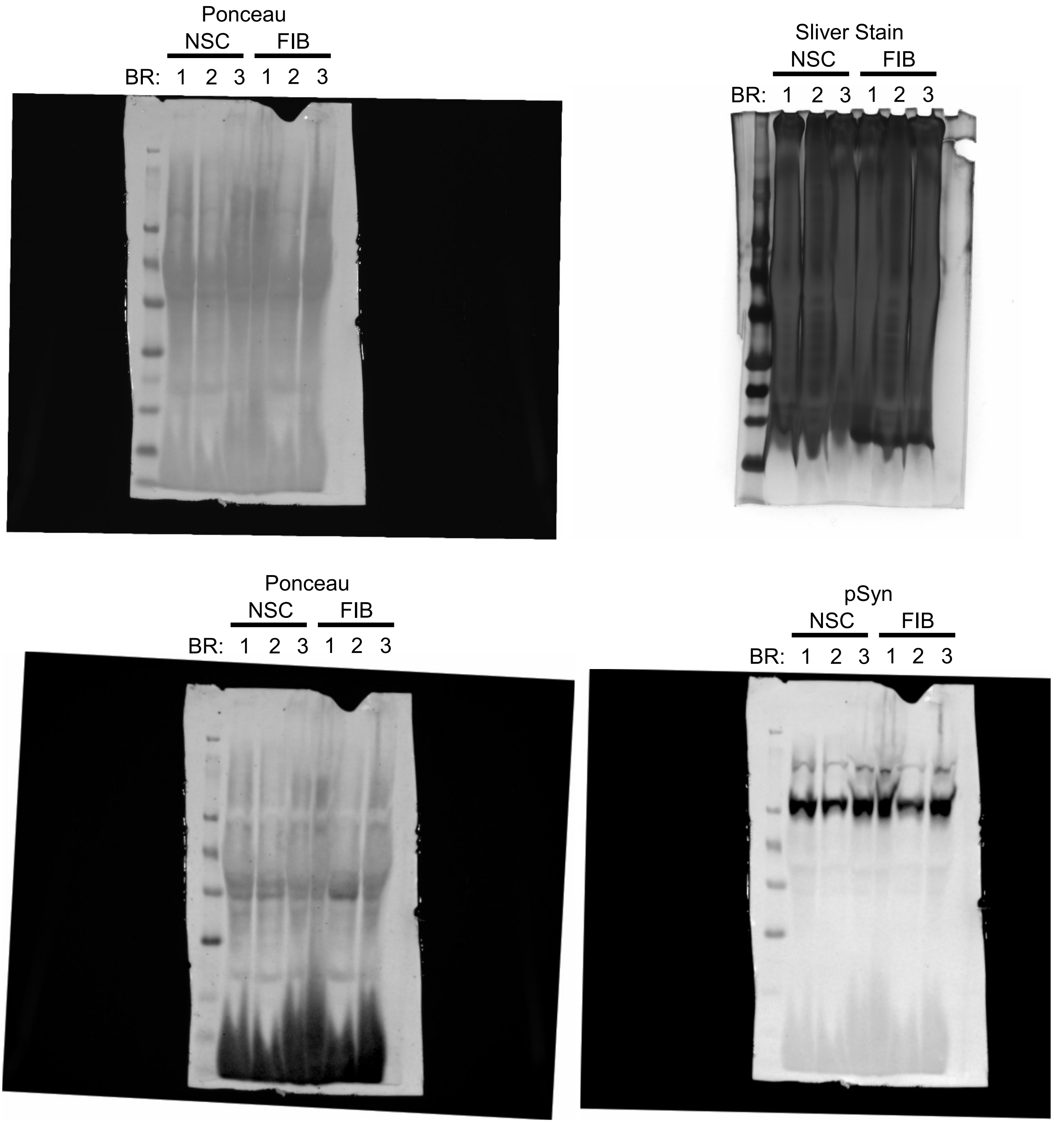
Ponceau
NSC
FIB
BR: 1 2 3 1 2 3
Sliver Stain
NSC
FIB
BR: 1 2 3 1 2 3
Ponceau
NSC
FIB
BR: 1 2 3 1 2 3
pSyn
NSC
FIB
BR: 1 2 3 1 2 3

Fig S9B

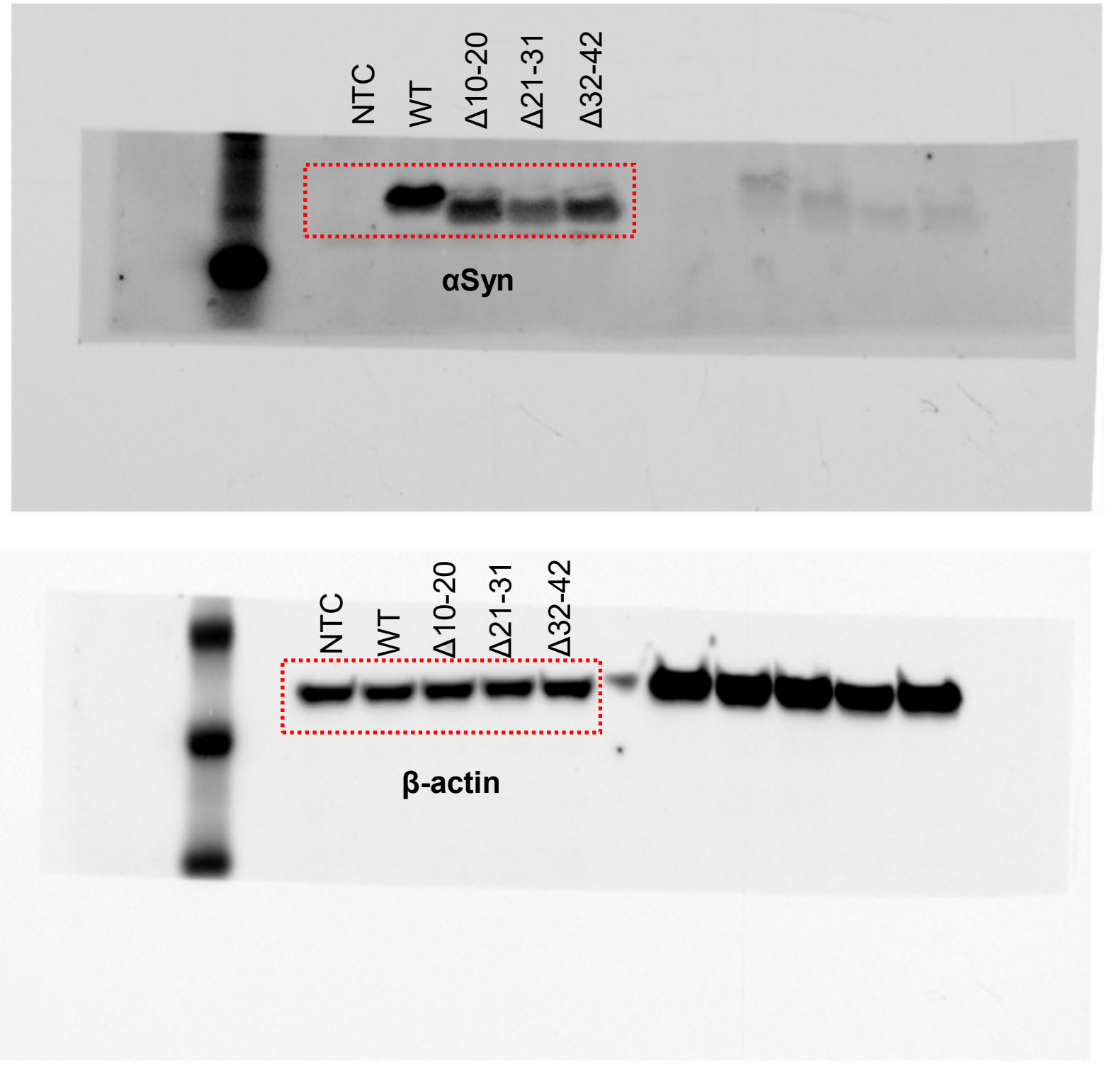
NTC
WT
Δ10-20
Δ21-31
Δ32-42
αSyn
NTC
WT
Δ10-20
Δ21-31
Δ32-42
β-actin

Fig S10A

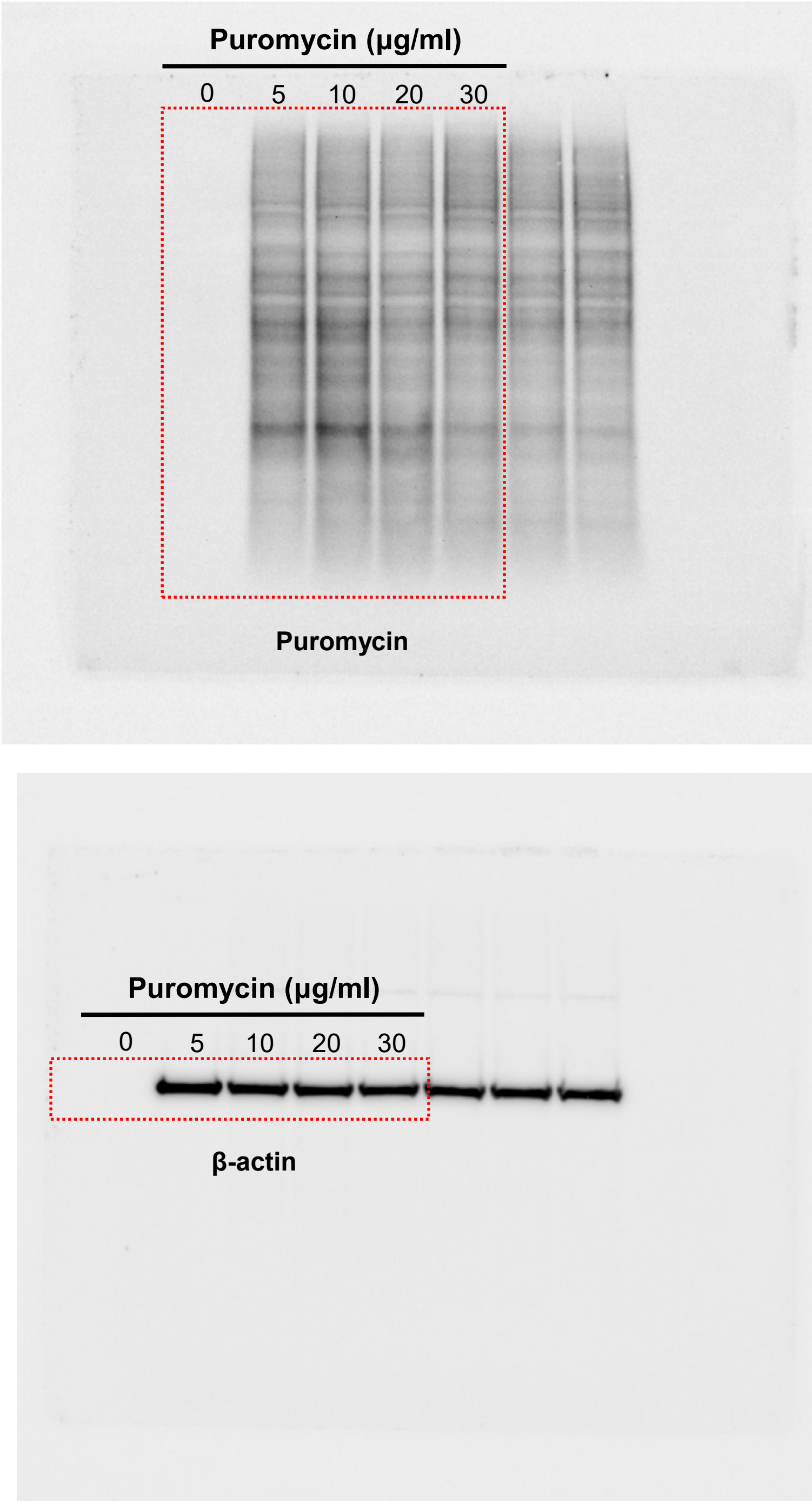

Fig S10B

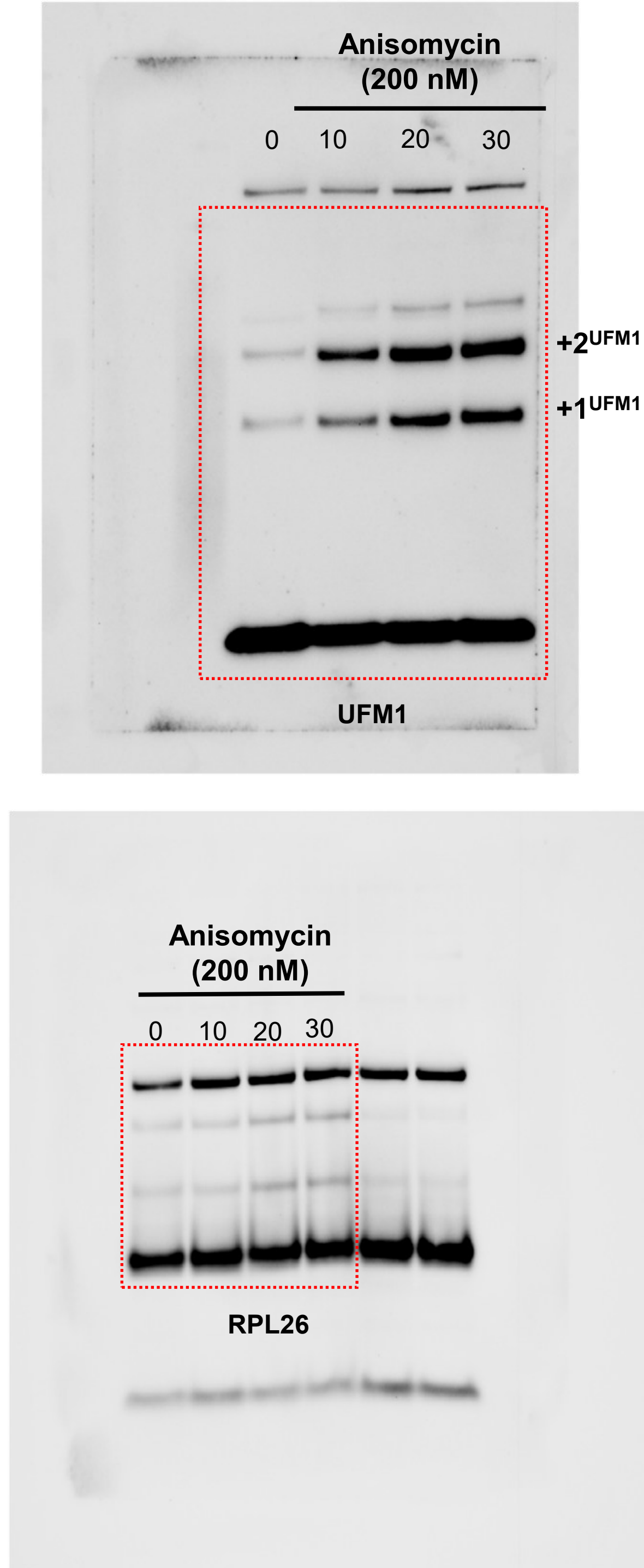

Fig S11A

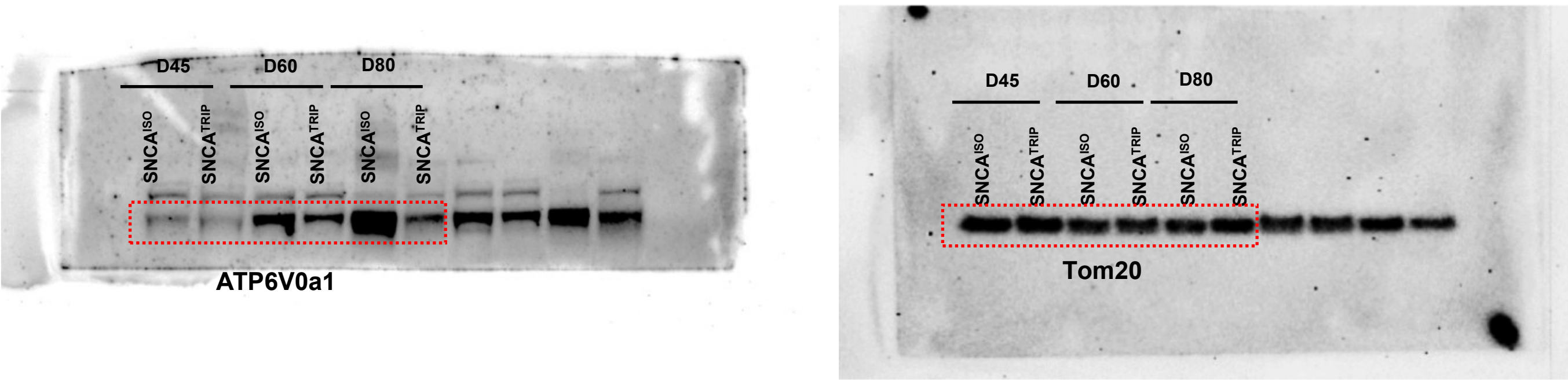

Fig S11D

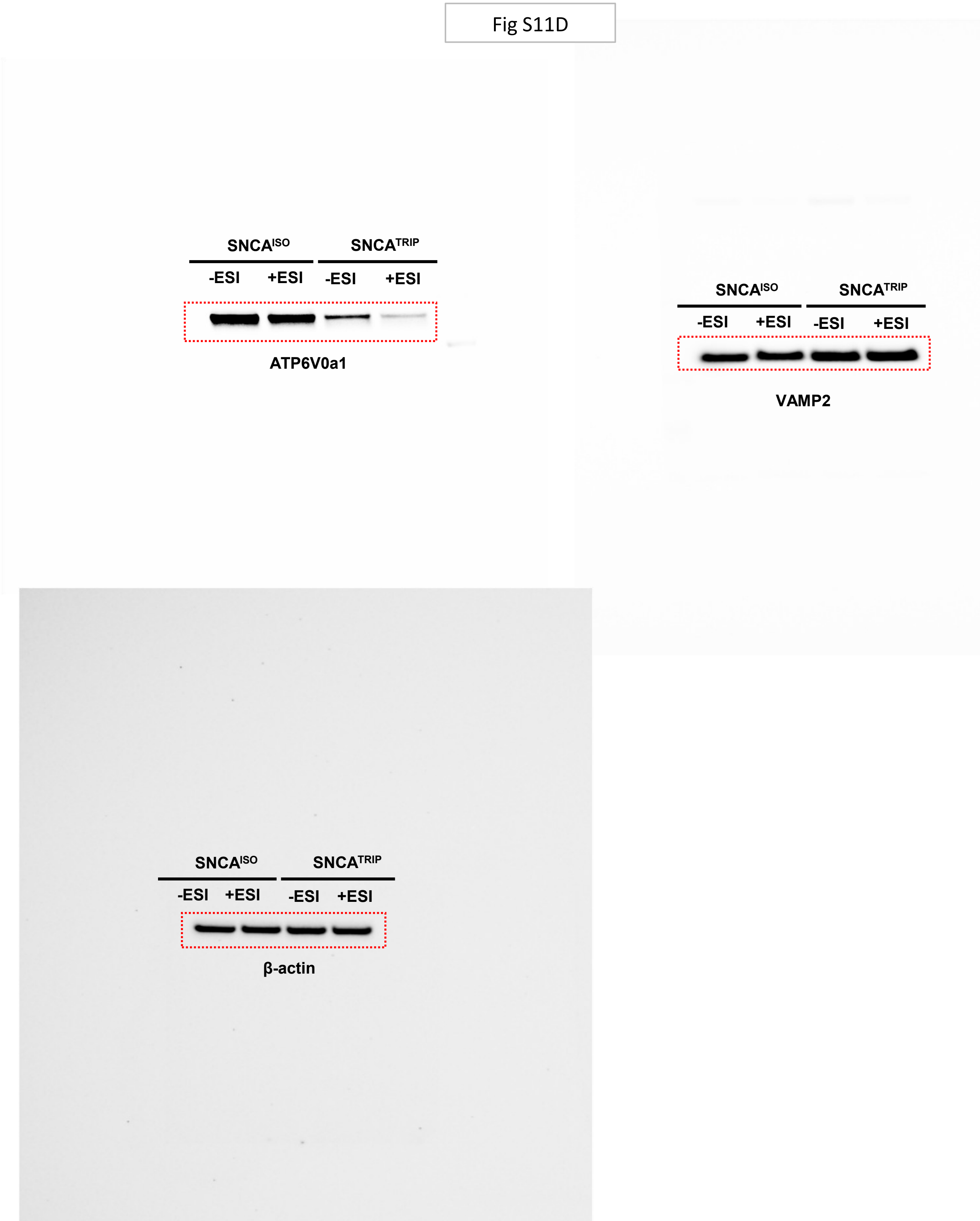

Fig S11F

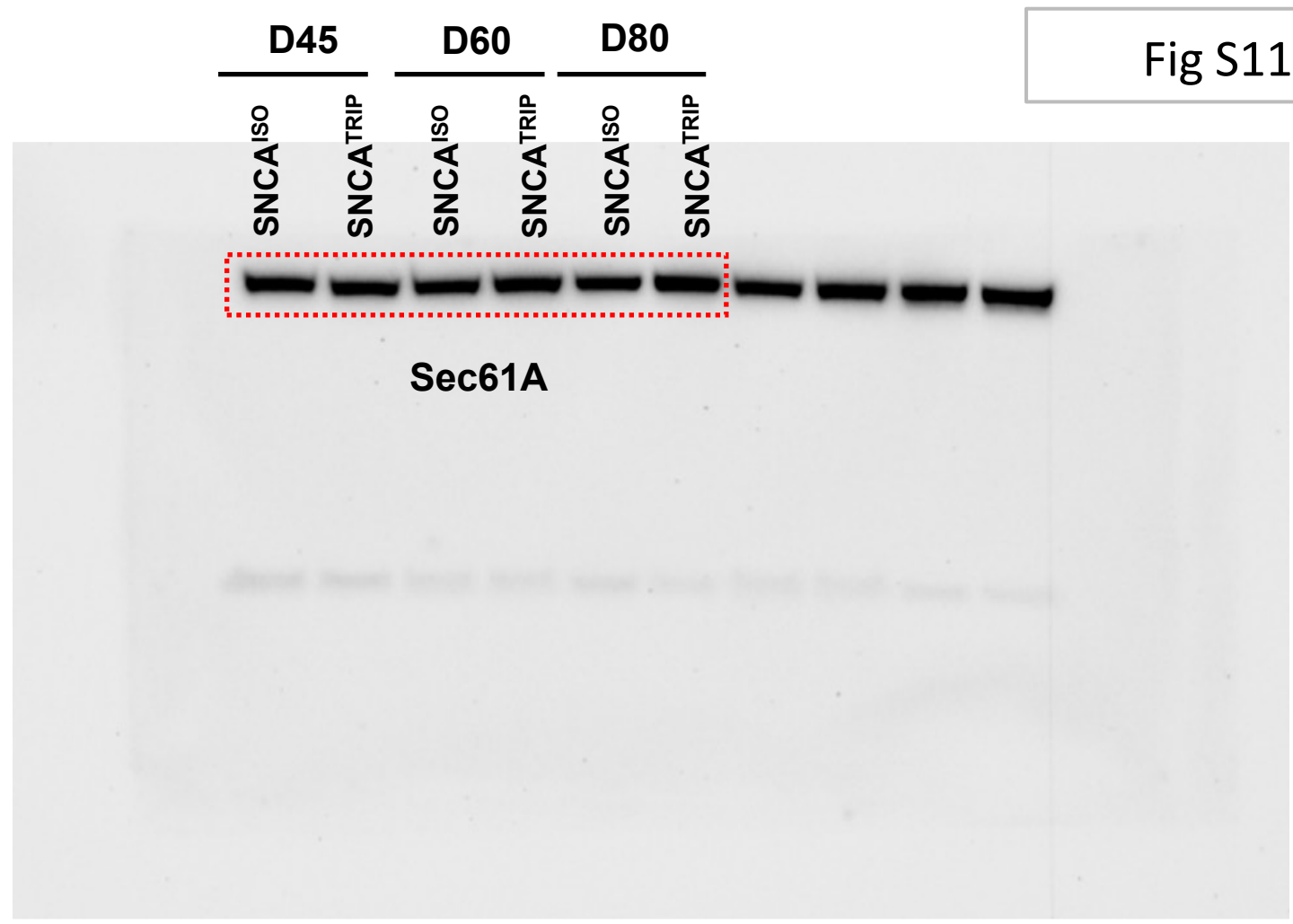
D45
D60
D80
SNCA^ISO
SNCA^TRIP
SNCA^ISO
SNCA^TRIP
SNCA^ISO
SNCA^TRIP
Sec61A

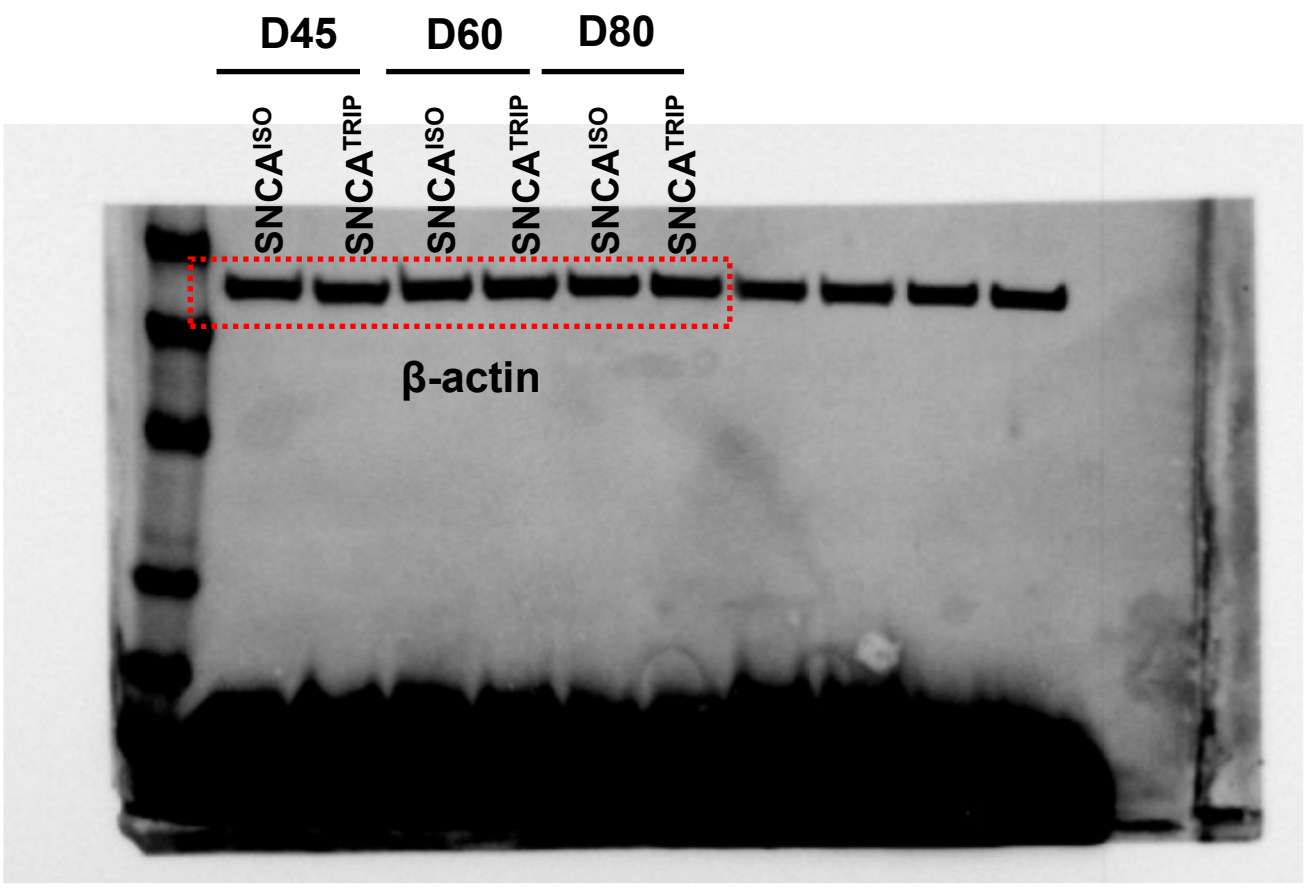
D45
D60
D80
SNCA^ISO
SNCA^TRIP
SNCA^ISO
SNCA^TRIP
SNCA^ISO
SNCA^TRIP
β-actin

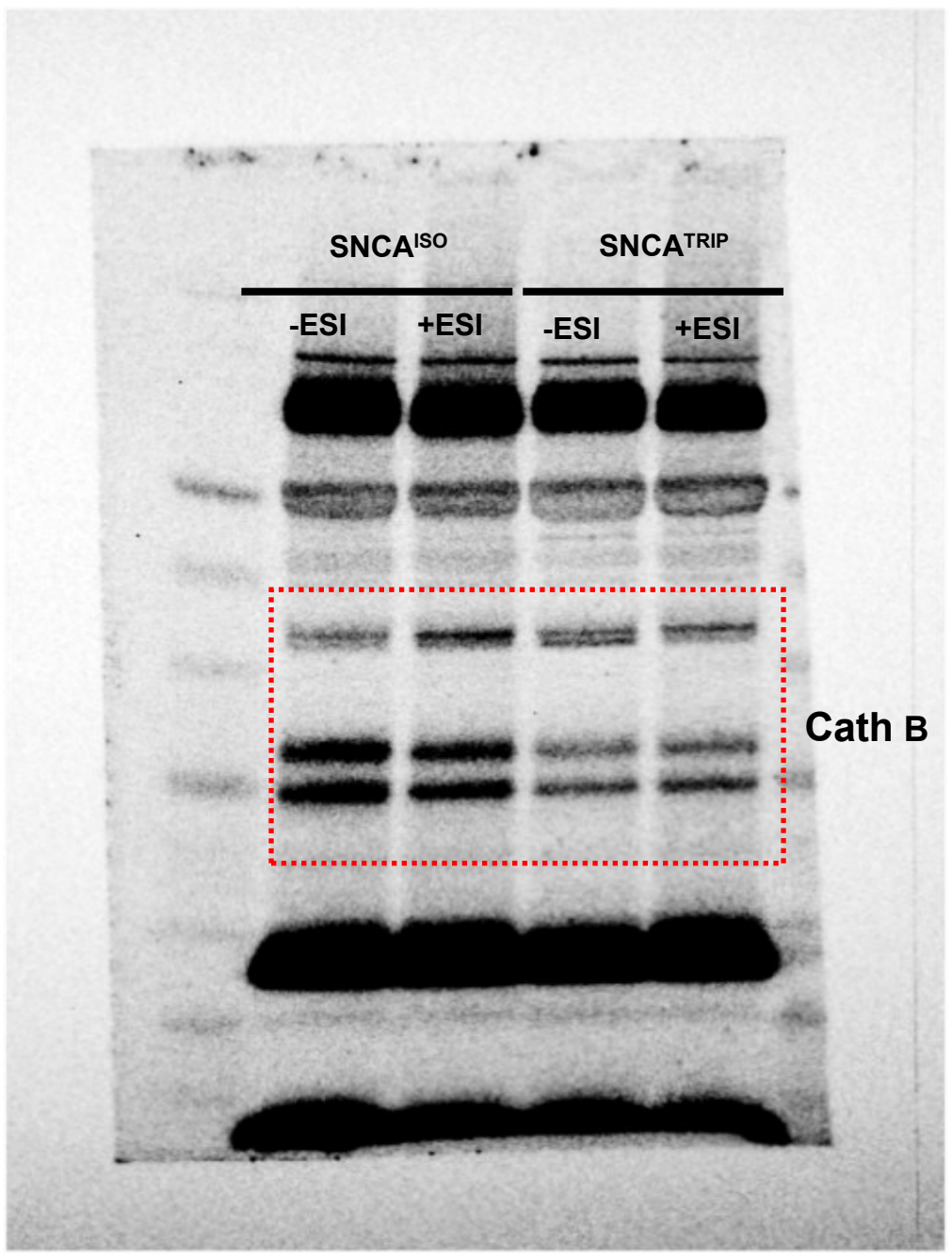
SNCA^ISO
SNCA^TRIP
-ESI
+ESI
-ESI
+ESI
Cath B

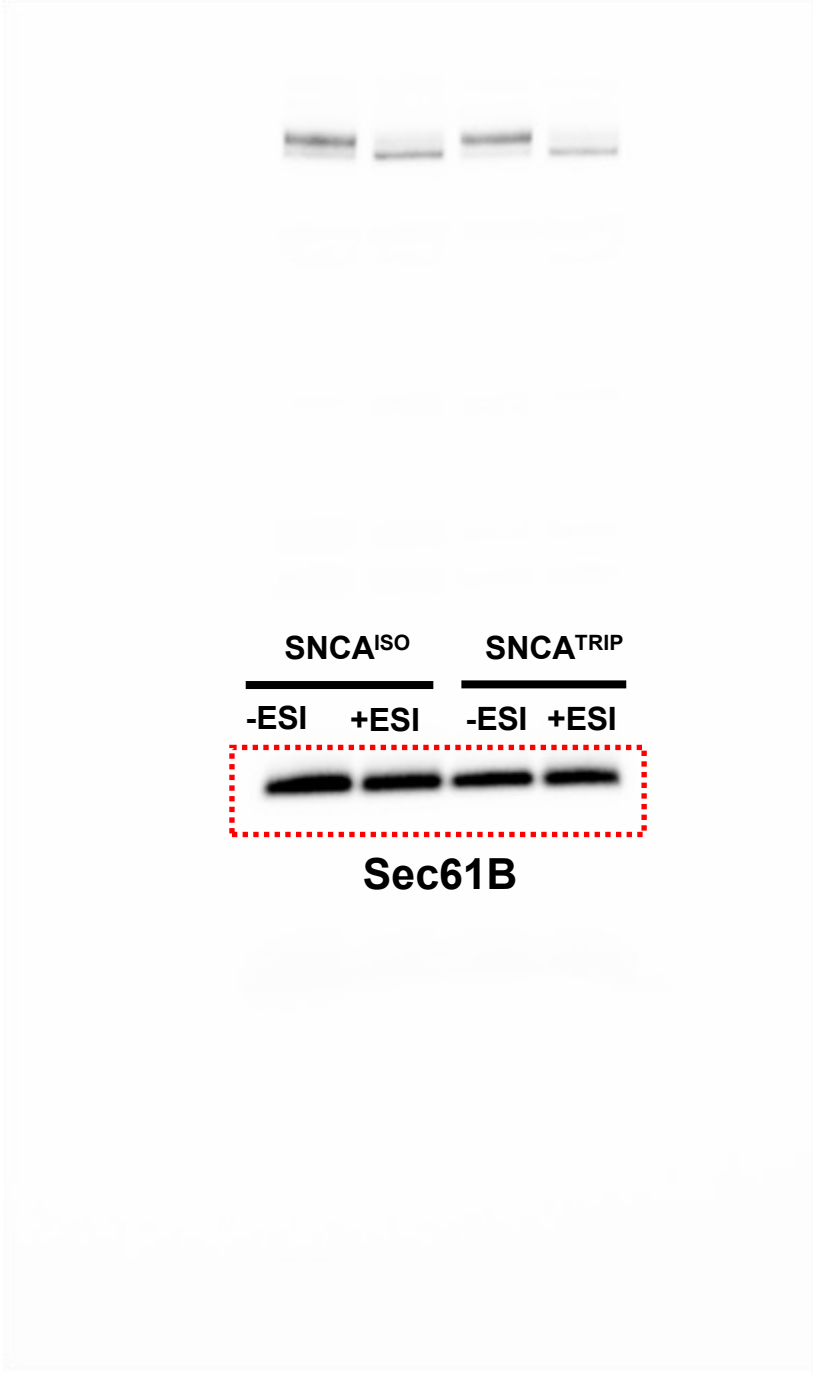
SNCA^ISO
SNCA^TRIP
-ESI
+ESI
-ESI
+ESI
Sec61B

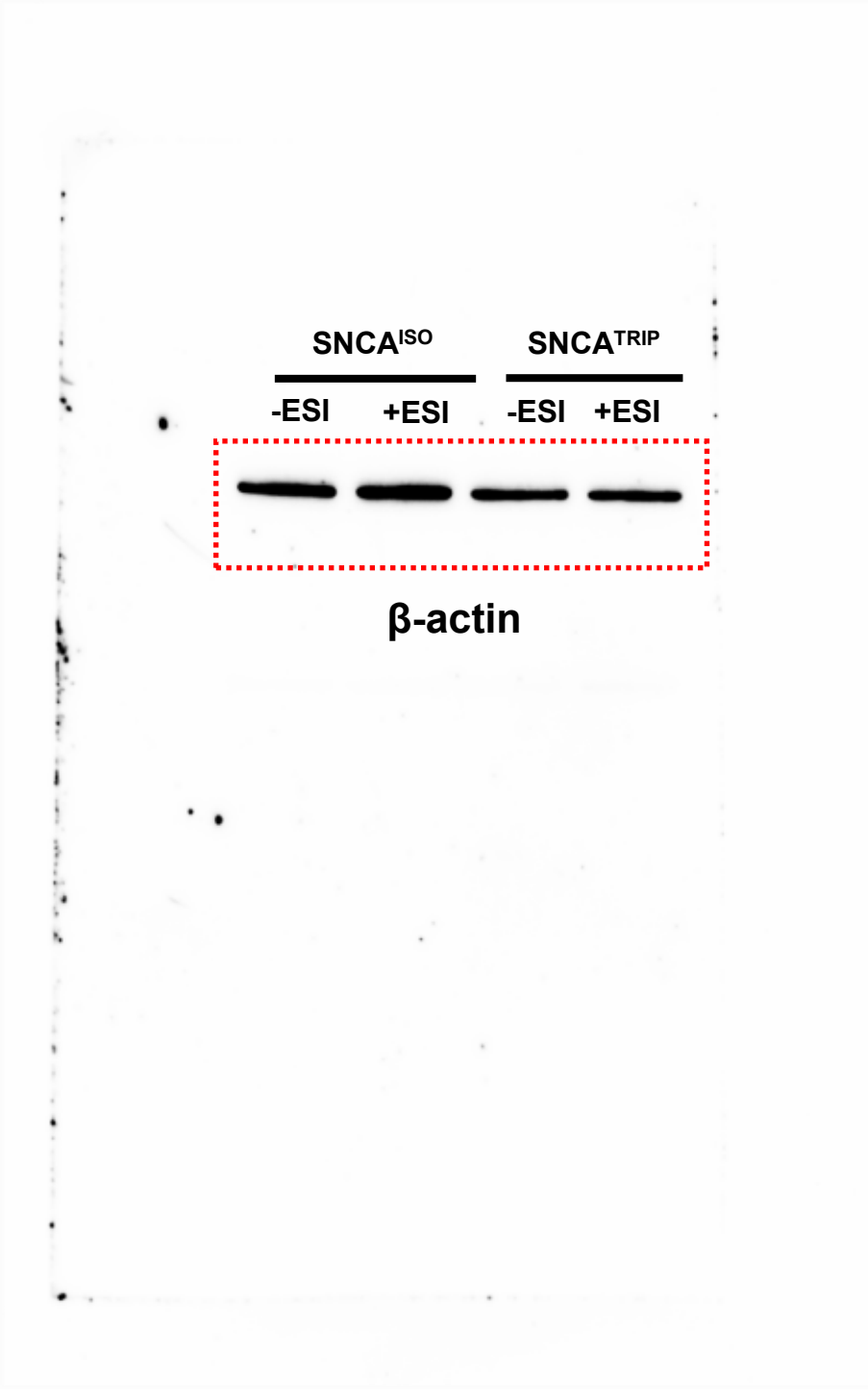
SNCA^ISO
SNCA^TRIP
-ESI
+ESI
-ESI
+ESI
β-actin

Fig S13A

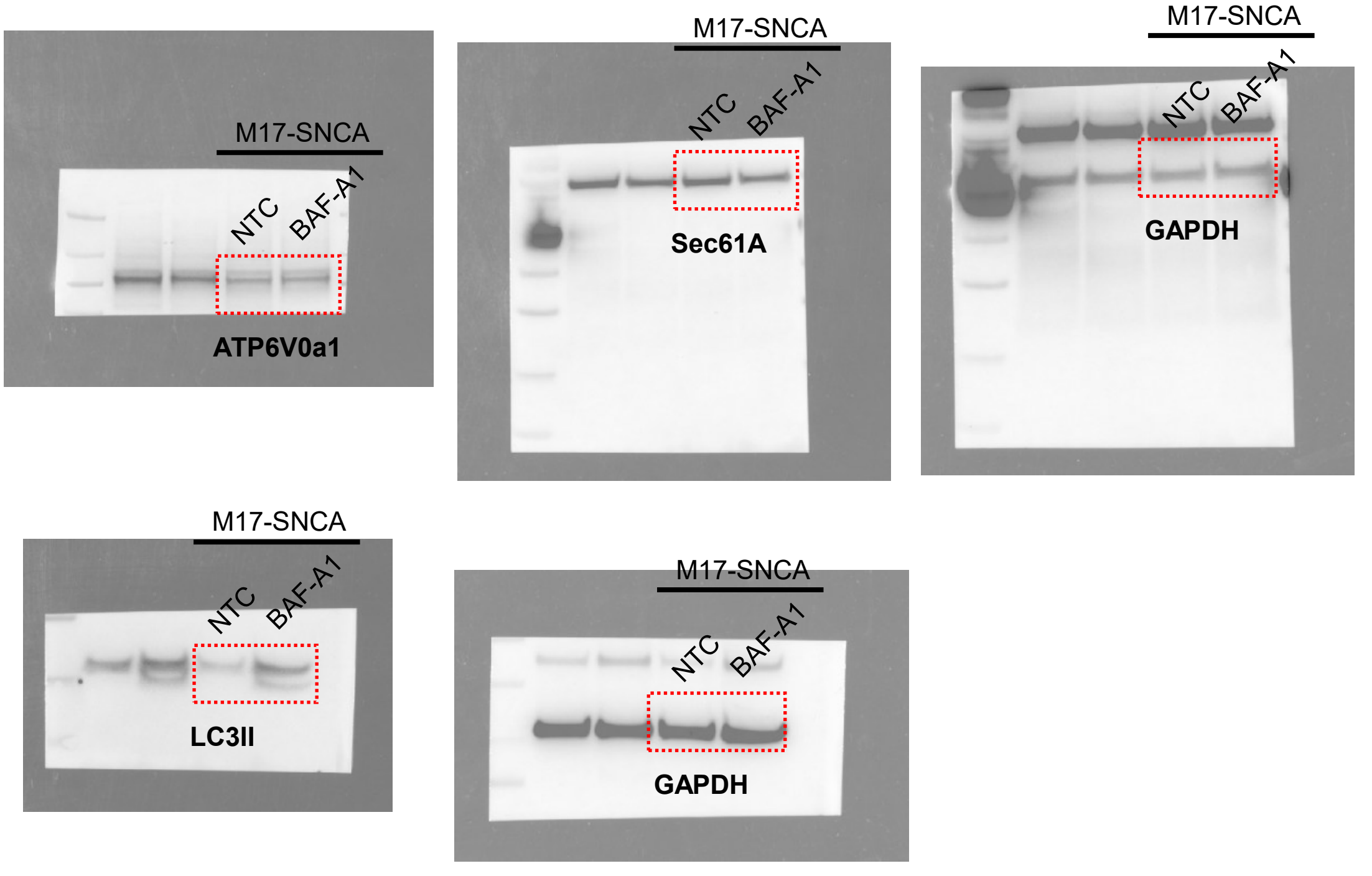
M17-SNCA
NTC
BAF-A1
ATP6V0a1
M17-SNCA
NTC
BAF-A1
Sec61A
M17-SNCA
NTC
BAF-A1
GAPDH
M17-SNCA
NTC
BAF-A1
LC3II
M17-SNCA
NTC
BAF-A1
GAPDH

Fig S15C

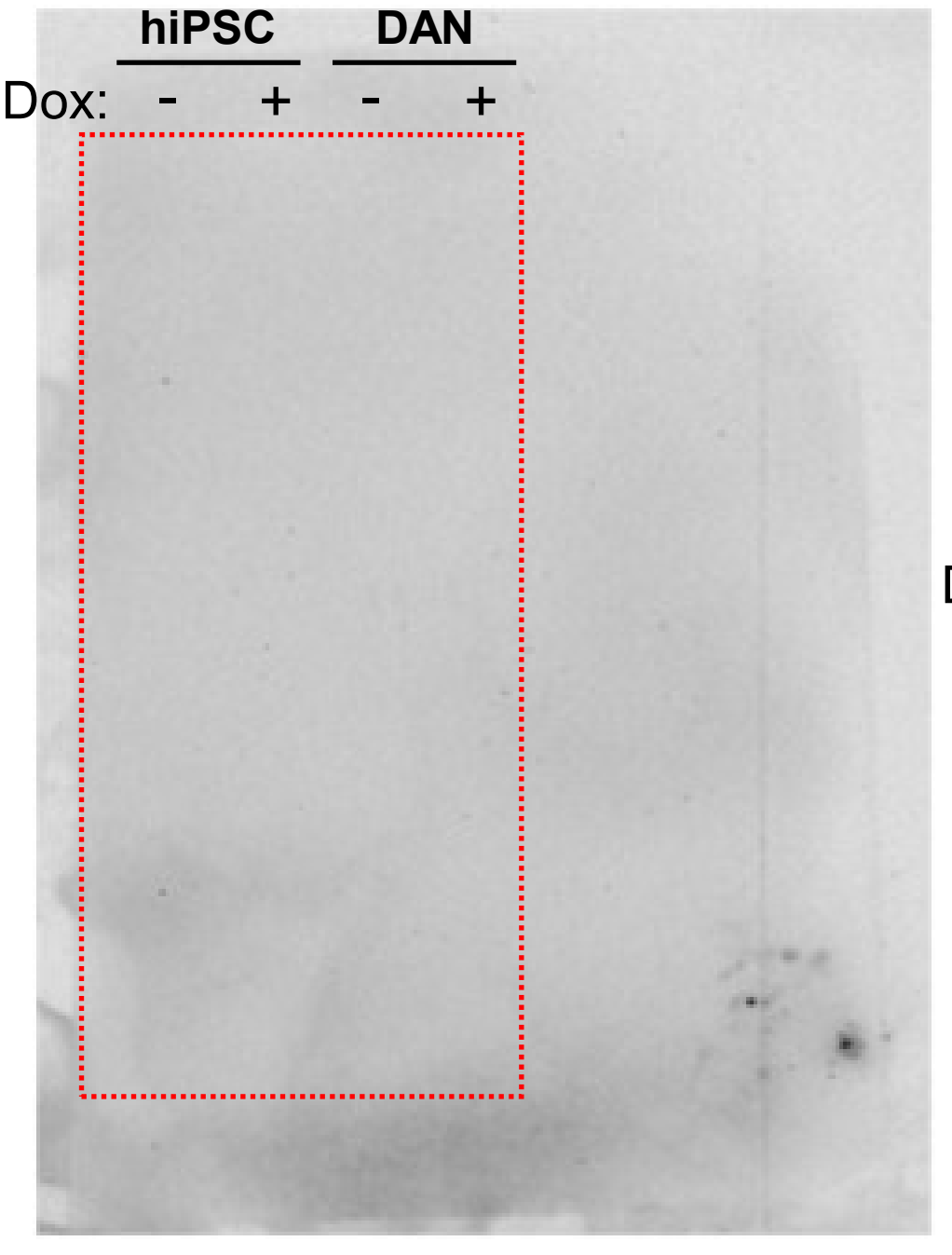

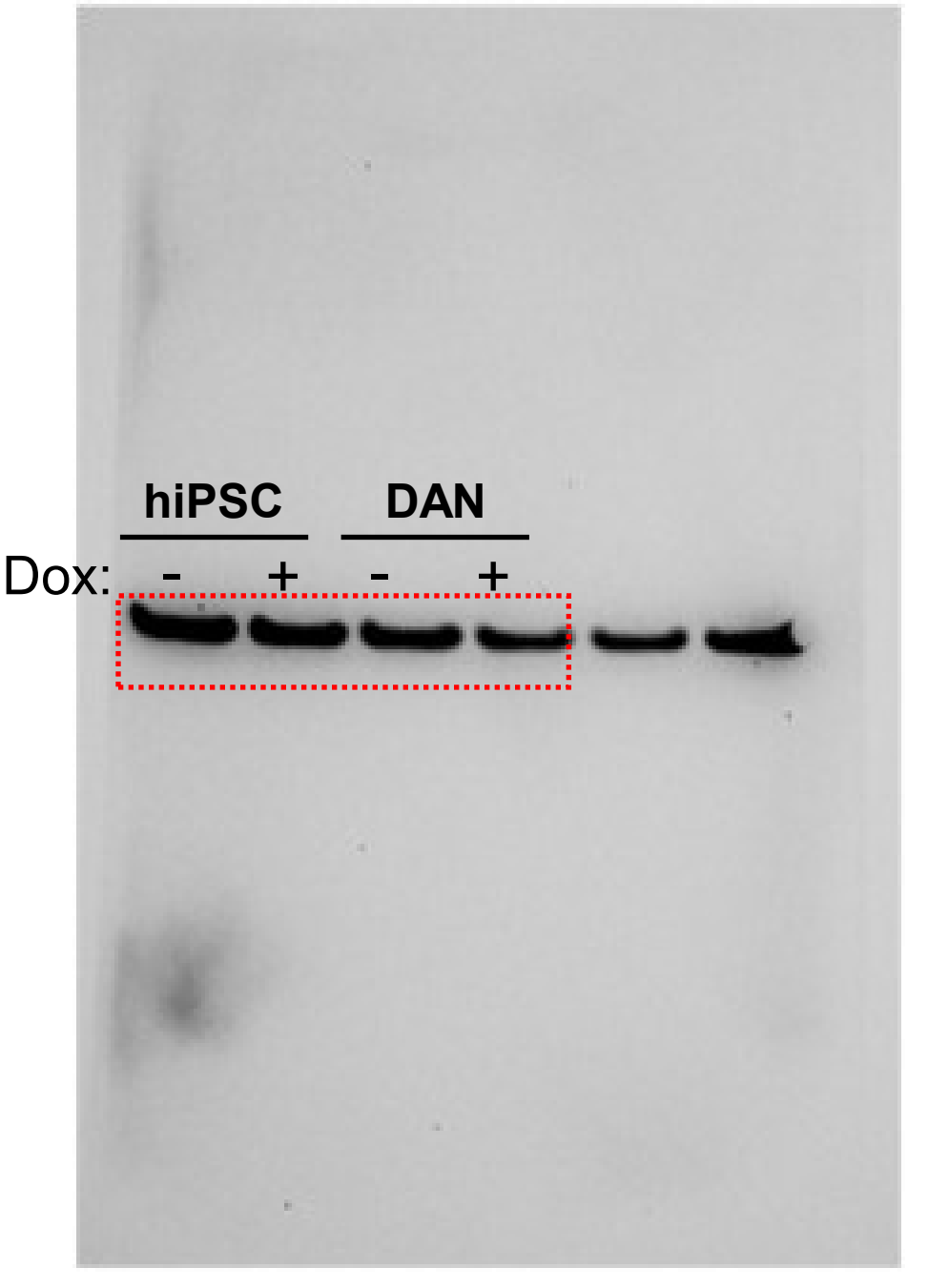

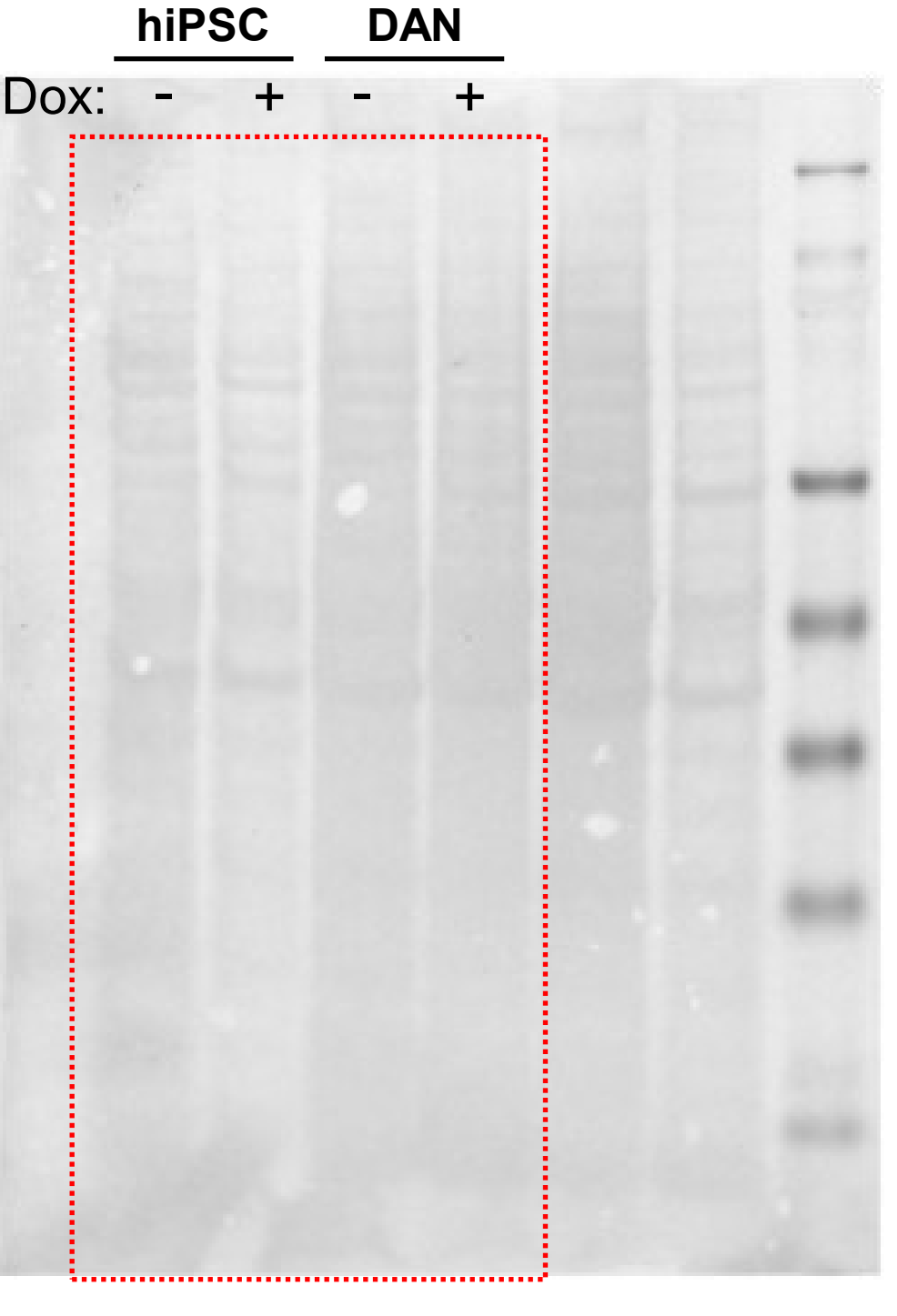

Supplement: Supplementary file 1 [file 41467_2026_76173_MOESM1_ESM.pdf]
