# Supplementary material for: α-Synuclein blocks endoplasmic reticulum co-translational protein translocation early in Parkinson’s disease
Source: Nat Commun. 2026 Aug 6;17:7768. doi: 10.1038/s41467-026-76173-4 (PMC13448182; doi:10.1038/s41467-026-76173-4)
Supplement: Supplementary file 2 [file 41467_2026_76173_MOESM2_ESM.pdf]

Title: Supplementary Data 1

Description: Proteins biotinylated by αSyn-APEX with and without seeded aggregation over time.

Title: Supplementary Data 2

Description: Differentially expressed genes (DEGs) in SNCATRIP hiPSC-DANs at D52 vs D60.

Title: Supplementary Data 3

Description: Differentially expressed genes (DEGs) in SNCATRIP and isogenic control hiPSC-DANs across pseudotime trajectory.

Title: Supplementary Data 4

Description: Information on single cell RNA-sequencing numbers and reads.
