# Supplementary material for: α-Synuclein blocks endoplasmic reticulum co-translational protein translocation early in Parkinson’s disease
Source: Nat Commun. 2026 Aug 6;17:7768. doi: 10.1038/s41467-026-76173-4 (PMC13448182; doi:10.1038/s41467-026-76173-4)
Supplement: Supplementary file 3 [file 41467_2026_76173_MOESM7_ESM.pdf]

nature portfolio

Corresponding author(s): George Tofaris

Last updated by author(s): May 16, 2026

Reporting Summary

Nature Portfolio wishes to improve the reproducibility of the work that we publish. This form provides structure for consistency and transparency in reporting. For further information on Nature Portfolio policies, see our Editorial Policies and the Editorial Policy Checklist.

Statistics

For all statistical analyses, confirm that the following items are present in the figure legend, table legend, main text, or Methods section.

n/a	Confirmed	
[ ]	[x]	The exact sample size (*n*) for each experimental group/condition, given as a discrete number and unit of measurement
[ ]	[x]	A statement on whether measurements were taken from distinct samples or whether the same sample was measured repeatedly
[ ]	[x]	The statistical test(s) used AND whether they are one- or two-sided<br>*Only common tests should be described solely by name; describe more complex techniques in the Methods section.*
[ ]	[x]	A description of all covariates tested
[ ]	[x]	A description of any assumptions or corrections, such as tests of normality and adjustment for multiple comparisons
[ ]	[x]	A full description of the statistical parameters including central tendency (e.g. means) or other basic estimates (e.g. regression coefficient) AND variation (e.g. standard deviation) or associated estimates of uncertainty (e.g. confidence intervals)
[ ]	[x]	For null hypothesis testing, the test statistic (e.g. *F*, *t*, *r*) with confidence intervals, effect sizes, degrees of freedom and *P* value noted<br>*Give P values as exact values whenever suitable.*
[x]	[ ]	For Bayesian analysis, information on the choice of priors and Markov chain Monte Carlo settings
[x]	[ ]	For hierarchical and complex designs, identification of the appropriate level for tests and full reporting of outcomes
[ ]	[x]	Estimates of effect sizes (e.g. Cohen's *d*, Pearson's *r*), indicating how they were calculated

Our web collection on statistics for biologists contains articles on many of the points above.

Software and code

Policy information about availability of computer code

Data collection

Confocal microscopy images were obtained using Zeiss LSM 980 microscope (Zeiss, Germany).
VisiTech-iSIM coupled to an Olympus IX83 microscope and a Hamamatsu ORCA-Quest qCMOS camera.
EVOS M7000 (Thermo Fisher).
TEM in a Jeol 1400 transmission electron microscope was used to assess morphology of the de novo assembled and PMCA amplified !SYN assemblies.
Time resolved FRET data were obtained using CLARIOstar Plus (BMG Labtech, Germany).
Q Exactive Orbitrap™ mass spectrometer (Thermo Fisher Scientific).
For RNASeq, sequencing was performed with Illumina NovaSeq 6000 (S4) using 150 bp paired end reads.
Immunoblots were visualised using a ChemiDoc Imaging system (Bio-Rad).
Structural data for human Sec61A were acquired from the RCSB Protein Data Bank (PDB); PDB ID: 8DNV.
All structure predictions were generated using ColabFold, a fast and reliable software variant of AlphaFold2.

Data analysis

Image analysis was performed using ImageJ (v1.54f).
3D renders were generated in ImarisViewer (v10.1.1).
Deconvolution was performed in cellSens Dimensions (v4.2.1).
Airyscan post-processing was performed in Zen Blue (v3.3.89).
Quantification of mass spectrometry data was performed using DIA-NN (v1.8.1).
Mass spectrometry peptide data were quantitatively analysed in Perseus (version 1.6.15.0).
Immunoblot band intensity was quantified by densitometry using Image Lab software (Bio-Rad, v6.1).
Gene Ontology (GO) analyses were performed using ShinyGO (v0.77).

Protein-protein interaction (PPI) networks were mapped and k-means clustering was performed using STRING-db (v12.0).
All STRING networks were imported into Cytoscape (v3.10.1) using the StringApp plugin for conversion into figure diagrams.
Structural data were visualized and colour-coded in UCSF ChimeraX (v1.6.1).
Cell-type deconvolution of the bulk RNAseq samples was conducted using MuSiC (v0.1.1).
Kallisto (v0.46.2) was used to estimate abundance for Ensembl (release 88) protein-coding genes from RNAseq reads.
Count data were imported into R (v4.0.3) using Tximport (v1.18.0), genes with <10 reads across the six samples used in the comparison were filtered out. Differential gene expression was performed in DESeq2 (v1.30.0).
Gene Set Enrichment Analysis (GSEA) was performed using the Fgsea package (v1.16.0)46 in R (v4.0.3).

For manuscripts utilizing custom algorithms or software that are central to the research but not yet described in published literature, software must be made available to editors and reviewers. We strongly encourage code deposition in a community repository (e.g. GitHub). See the Nature Portfolio guidelines for submitting code & software for further information.

Data

Policy information about availability of data

All manuscripts must include a data availability statement. This statement should provide the following information, where applicable:

- Accession codes, unique identifiers, or web links for publicly available datasets
- A description of any restrictions on data availability
- For clinical datasets or third party data, please ensure that the statement adheres to our policy

All data supporting the findings of this manuscript are available from the corresponding authors upon reasonable request. Correspondence and requests for materials should be addressed to george.tofaris@ndcn.ox.ac.uk.

Bulk RNAseq data are available at GEO under accession numbers GSE276377 and GSE171999.
To review GSE276377 go to https://www.ncbi.nlm.nih.gov/geo/query/acc.cgi?acc=GSE276377. Enter token uzolocootbkdtud into the box.
To review GSE171999 go to: https://www.ncbi.nlm.nih.gov/geo/query/acc.cgi?acc=GSE171999.

Single cell RNAseq data are available at GEO under accession number GSE276559.
To review go to https://www.ncbi.nlm.nih.gov/geo/query/acc.cgi?acc=GSE276559. Enter token mdevmkuoprsjdoz into the box.

Proteomic data have been deposited to the ProteomeXchange Consortium via the PRIDE partner repository with the dataset identifier PXD055581.
To review PXD055581 go to https://www.ebi.ac.uk/pride/login and log in using the following details under log in: Username: reviewer_pxd055581@ebi.ac.uk.
Password: r8PpJyeS2686

Research involving human participants, their data, or biological material

Policy information about studies with human participants or human data. See also policy information about sex, gender (identity/presentation), and sexual orientation and race, ethnicity and racism.

Reporting on sex and gender	N/A as hiPSC lines with relevant mutations are extremely rare. Sex of patient derived material is reported in extended data tables 2 & 4
Reporting on race, ethnicity, or other socially relevant groupings	N/A
Population characteristics	Fresh frozen tissue was dissected from 3 control brains and 3 PD patient brains. Substantia nigra was dissected from each PD and control brain. All PD cases exhibited alpha-synuclein positive inclusions on neuropathological examination.
Recruitment	All brain samples were obtained from the Oxford Brain Bank. Consent forms were signed by the subjects themselves of their next of kin in accordance with UK ethics laws.
Ethics oversight	Ethical approval for the use of human tissue was obtained from the Oxford C REC Ethics Committee (No. 15/SC/0639).

Note that full information on the approval of the study protocol must also be provided in the manuscript.

Field-specific reporting

Please select the one below that is the best fit for your research. If you are not sure, read the appropriate sections before making your selection.

[x] Life sciences   [ ] Behavioural & social sciences   [ ] Ecological, evolutionary & environmental sciences

For a reference copy of the document with all sections, see nature.com/documents/nr-reporting-summary-flat.pdf

Life sciences study design

All studies must disclose on these points even when the disclosure is negative.

Sample size	No sample size calculation was performed. Biological triplicates were selected as the minimum necessary for quantitative statistical analysis

Data exclusions	No data were excluded from any analyses
Replication	All experiments were repeated for at least three biological replicates and all replication was successful
Randomization	All cells in all images were randomly selected at the time of acquisition, all cells in all images were quantified for analysis
Blinding	Investigators were blinded during microscopy acquisition and analysis where possible. Blinding was not possible in conditions involving seeded aggregation as the observed phenotype was too binary to be ambiguous

Reporting for specific materials, systems and methods

We require information from authors about some types of materials, experimental systems and methods used in many studies. Here, indicate whether each material, system or method listed is relevant to your study. If you are not sure if a list item applies to your research, read the appropriate section before selecting a response.

Materials & experimental systems

n/a	Involved in the study	
☐	☒	Antibodies
☐	☒	Eukaryotic cell lines
☒	☐	Palaeontology and archaeology
☒	☐	Animals and other organisms
☒	☐	Clinical data
☒	☐	Dual use research of concern
☒	☐	Plants

Methods

n/a	Involved in the study	
☒	☐	ChIP-seq
☒	☐	Flow cytometry
☒	☐	MRI-based neuroimaging

Antibodies

Antibodies used	Streptavidin, Alexa Fluor™ 488 conjugate (Invitrogen, S11223, 1:1000), Streptavidin, Alexa Fluor™ 568 conjugate (Invitrogen, S11226, 1:1000), mouse anti-V5 monoclonal (Invitrogen, R960-25 [SV5-Pk1], 1:500), rabbit anti-V5 monoclonal (Invitrogen, MA5-32053 [SY30-01], 1:500), mouse anti-Tuj1 monoclonal (BioLegend, 801201 [TUJ1], 1:1000), rabbit anti-Tuj1 polyclonal (BioLegend, 802001, 1:1000), rabbit anti-alpha-synuclein (phosphor S129) monoclonal (Abcam, ab51253 [EP1536Y], 1:1000), chicken anti-MAP2 polyclonal (Abcam, ab92434, 1:1000), rat anti-DAT monoclonal (Merck, MAB369 [DAT-Nt], 1:500), mouse anti-TH monoclonal (Merck, MAB318 [LNC1], 1:1000), rabbit anti-GFAP polyclonal (Novus, NB300-141, 1:1000), rabbit anti-SOX2 polyclonal (Abcam, ab97959, 1:1000), rat anti-Ctip2 monoclonal (Abcam, ab18465 [25B6], 1:500), mouse anti-puromycin monoclonal (Merck, MABE343 [12D10], 1:5000), rabbit anti-Sec61A monoclonal (Abcam, ab183046 [EPR14379], 1:1000), goat anti-Sec61A polyclonal (Abcam, ab1327, 1:1000), rabbit anti-ATP6V0a1 polyclonal (Proteintech, 13828-1-AP, 1:1000), rabbit anti-ATP6V0a1 polyclonal (Novus, NBP1-89342, 1:1000), rabbit anti-UFM1 monoclonal (Abcam, ab109305, [EPR4264(2)], 1:1000), sheep anti-UFM1 polyclonal (Bio-Techne, AF8237, 1:1000), sheep anti-GM130 polyclonal (Bio-Techne, AF8199, 1:1000) goat anti-LAMP2 polyclonal (Bio-Techne, AF6228, 1:1000), rabbit anti-GBA polyclonal (Sigma, G4171, 1:1000), mouse anti-β-Actin monoclonal (Sigma, A1978 [AC-15], 1:5000), rabbit anti-Cas9 polyclonal (Takara Bio, 632607, 1:1000), mouse anti-TetR monoclonal (Takara Bio, 631132, 1:1000), mouse anti-alpha-synuclein monoclonal (BD Transduction Laboratories, 610787 [42], 1:1000), rabbit anti-Sec61B polyclonal (Proteintech, 15087-1-AP, 1:1000), rabbit anti-VAMP2 monoclonal (Abcam, ab18169 [EPR12790], 1:1000), rabbit anti-SQS (FDFT1) polyclonal (Proteintech, 13128-1-AP, 1:1000), mouse anti-Tom20 monoclonal (Santa Cruz Biotechnology, sc-17764 [F-10], 1:1000), rabbit anti-Cathepsin B polyclonal (Proteintech, 12216-1-AP, 1:1000), rabbit anti-Phospho-p38 MAPK (Thr180/Tyr182) polyclonal (Cell Signaling Technology, 9211, 1:1000), rabbit anti-p38 MAPK polyclonal (Cell Signaling Technology, 9212, 1:1000), rabbit anti-XBP1S polyclonal (Proteintech, 24868-1-AP, 1:1000), rabbit anti-ATF4 polyclonal (Proteintech, 10835-1-AP, 1:1000), rabbit anti-CHOP polyclonal (Proteintech, 15204-1-AP, 1:1000), mouse anti-PSMD11 monoclonal (GeneTex, GTX50018 [AT1F4], 1:1000), rabbit anti-alpha-synuclein aggregate - conformation-specific monoclonal (Abcam, ab209538 [MJFR-14-6-4-2], 1:20000), mouse Anti-Aggregated a-Synuclein Antibody (Sigma, MABN389[5G4], 1:5000). Alexa Fluor™ 488 (A11029, A11034, A11039, A11006), 568 (A11031, A11036, A11041), and 647 (A21236, A21245, A21449) (Life Technologies, 1:1000) fluorescent secondary antibodies, streptavidin, horseradish peroxidase conjugate (Invitrogen, S911, 1:5000), rabbit anti-HRP (Life Technologies, G-21234, 1:10000), mouse anti-HRP (Life Technologies, G-21040, 1:10000)
Validation	All antibodies used have been validated previously and are commercially available. All validation data is available on the respective company's website.

Eukaryotic cell lines

Policy information about cell lines and Sex and Gender in Research

Cell line source(s)	All hiPSC lines were previously generated and characterised in studies indicated in Extended Data Table 4
Authentication	All hiPSC lines were extensively authenticated by confirming their differentiation into dopaminergic neurons with immunofluorescence microscopy and RNA-seq analysis as shown in Extended Data Figure 1a and Extended Data Figure 6. Additionally, the dCas9-hiPSC line was karyotyped and STR profiled by the supplier as shown in Extended Data Figure 14 and Extended Data Figure 15.
Mycoplasma contamination	All hiPSC lines were tested and confirmed negative for mycoplasma contamination throughout experimentation.

Commonly misidentified lines (See ICLAC register)	No commonly misidentified cell lines were used in this study.

Plants

Seed stocks	*Report on the source of all seed stocks or other plant material used. If applicable, state the seed stock centre and catalogue number. If plant specimens were collected from the field, describe the collection location, date and sampling procedures.*
Novel plant genotypes	*Describe the methods by which all novel plant genotypes were produced. This includes those generated by transgenic approaches, gene editing, chemical/radiation-based mutagenesis and hybridization. For transgenic lines, describe the transformation method, the number of independent lines analyzed and the generation upon which experiments were performed. For gene-edited lines, describe the editor used, the endogenous sequence targeted for editing, the targeting guide RNA sequence (if applicable) and how the editor was applied.*
Authentication	*Describe any authentication procedures for each seed stock used or novel genotype generated. Describe any experiments used to assess the effect of a mutation and, where applicable, how potential secondary effects (e.g. second site T-DNA insertions, mosiacism, off-target gene editing) were examined.*
