# Supplementary material for: α-Synuclein blocks endoplasmic reticulum co-translational protein translocation early in Parkinson’s disease
Source: Nat Commun. 2026 Aug 6;17:7768. doi: 10.1038/s41467-026-76173-4 (PMC13448182; doi:10.1038/s41467-026-76173-4)
Supplement: Supplementary file 4 [file 41467_2026_76173_MOESM8_ESM.pdf]

nature portfolio

Peer Review File

α-Synuclein blocks endoplasmic reticulum co-translational protein translocation early in Parkinson's disease

Corresponding Author: Professor George Tofaris

This file contains all reviewer reports in order by version, followed by all author rebuttals in order by version.

Version 0:

Reviewer comments:

Reviewer #1

(Remarks to the Author)
Although the new data substantially strengthen the manuscript, several key mechanistic claims are still not directly demonstrated. In particular, there is no direct evidence that intermediate, partially unfolded α-synuclein proteoforms physically occlude the translocon, nor that ribosome stalling occurs in human neurons under the conditions tested. Likewise, proteasome activation is inferred from prior literature and pharmacological manipulation, but proteasome activity was not directly measured in this system. I would therefore ask the authors to slightly tone down their wording in the main text and Discussion, and to explicitly frame these points as models that are consistent with, but not conclusively demonstrated by, the current data (e.g. avoiding statements that these mechanisms are "demonstrated" or "sufficient," and instead using formulations such as "our findings are consistent with a model in which …").

Reviewer #2

(Remarks to the Author)

The authors have made a number of revisions and provided additional data to address the issues raised by myself and the other reviewers. Most of the revisions addressed the points I raised and the manuscript is improved. However, several issues are not fully resolved, outlined below. Furthermore, the experiments suggested by reviewer 1 to correlate Sec61 interaction to the N-terminus of α-Synuclein seem at present unconvincing. These would need to be addressed prior to recommendation for publication.

The suggestion to directly monitor translocation by detecting non-translocated precursor (point 3) was addressed with the N-glycosylated substate Cathepsin B, however, the authors refer to the 42 kD pro and pre-pro forms. However as pro-cathepsin-B has two N-glycans, the non-translocated (pre-pro) form will lack the glycans and be smaller, albeit compensated by the uncleaved signal sequence so I suspect 2-3 kD smaller, such a species wasn't obvious in the ESI or SNCA-TRIP blots. As mentioned, it may be that this is rapidly degraded hence the need for proteasome inhibitor to detect the non-translocated precursor.
While the reduction in translocated forms of Sec61 substrates, but not GET client, is supportive, the direct demonstration of Sec61-translocation block over reduced translation remains.

The suggestion to use of Sec61 inhibitors to investigate whether inhibition of translocation leads to UFMylation looks to be fairly convincing (and an interesting finding) from the UFM blot (Fig. 4H), although if the Rpl26 blot panel is from the same samples, the levels of the mono-UFM band don't seem to match well with the UFM panel.

I didn't understand the comment on the conA puromycin reduction, "it is not surprising that the difference was not as prominent as with individual substrates as not all
glycosylated membrane proteins are translocon substrates.' Does 'translocon' here mean Sec61? Only translocated proteins can be N-glycosylated hence they need a translocon, but they may use different translocases to Sec61.

As suggested by reviewer 1, the authors carry out experiments to correlate removal of the potential N-terminal interacting region of Syn with translocation inhibition and interaction with Sec61.
These experiments are very difficult to interpret; the proximity assay shows a marked reduction with the Δ10-20 construct

and showed modest loss of translocation inhibition, but then showed similar cross-linking to Sec61α in the IP– although the data are not of highest quality – and I assume this is a denaturing IP (not co-IP as mentioned) to rigorously demonstrate direct crosslinking to Sec61α. In contrast, the Δ21-31and Δ32-42 showed similar interaction in the proximity assay yet even stronger crosslinking in the case of Δ21-31 but no cross-link at all in Δ32-42. Similarly, Δ21-31 construct has strong loss of inhibition while the Δ32-42 is similar to Δ10-20, thus there is no good correlation of Sec61 binding with translocation inhibition. The use of the lysine-based cross-linker DSG is perhaps not the best as there is the additional complication that the lysine residues that form the cross-links are lost in some truncations. Overall, I didn't find this aspect convincing

Reviewer #3

(Remarks to the Author)
The relevance, quality, and soundness of the presented research was already highlighted in my original assessment. All my suggestions to improve the work further have been sufficiently addressed in the revised version. The work should be published.

Version 1:

Reviewer comments:

Reviewer #1

(Remarks to the Author)
The authors have adequately addressed the concerns raised in my previous review. The manuscript has been significantly improved, and I believe that the revised version is suitable for publication in Nature Communications.

Reviewer #2

(Remarks to the Author)
My major concerns were that the authors have yet to formally distinguish between a reduced rate of ER translocation and reduced translation (with efficient translocation) both of which lead to the observed reduced delivery of proteins into the ER. The authors have still not been able to directly address this point by detecting the untranslocated form(s) of substrate protein(s). This may be as this species is rapidly degraded. It seems from tests their system does not tolerate proteasome inhibition well, which I suggested may help to visualise the untranslocated forms. If this is not possible to do this in this system, then the discussion could be altered to specifically raise this caveat. The new data with the Magic Red assay doesn’t really address this point, rather it just provides an independent validation that there is less translocated active cathepsin B which was largely established.

My other concern regarded the interpretation of the Sec61alpha interaction studies with the N-terminal a-Syn deletion mutants. Especially, as cross-linking, proximity and inhibition assays showed different and poorly consistent findings making it hard to make firm conclusions. The authors now have removed the proximity data and added quantification of the cross-link data. I still have the same caveats regarding the interpretation of the cross-link data due to the impacts of removing different lysines. But the cross-link data does provide independent evidence for the Sec61alpha/a-Syn interaction so could still be concluded with softened interpretation and mentioning that changes might be due to the removal of the lysines involved in cross-linking rather than a loss of interaction.

The other more minor points are now addressed.

Open Access This Peer Review File is licensed under a Creative Commons Attribution 4.0 International License, which permits use, sharing, adaptation, distribution and reproduction in any medium or format, as long as you give appropriate credit to the original author(s) and the source, provide a link to the Creative Commons license, and indicate if changes were made.
In cases where reviewers are anonymous, credit should be given to 'Anonymous Referee' and the source.
The images or other third party material in this Peer Review File are included in the article's Creative Commons license, unless indicated otherwise in a credit line to the material. If material is not included in the article's Creative Commons license and your intended use is not permitted by statutory regulation or exceeds the permitted use, you will need to obtain permission directly from the copyright holder.
To view a copy of this license, visit https://creativecommons.org/licenses/by/4.0/

RESPONSE TO REVIEWERS

Reviewer #1

Although the new data substantially strengthen the manuscript, several key mechanistic claims are still not directly demonstrated. In particular, there is no direct evidence that intermediate, partially unfolded α-synuclein proteoforms physically occlude the translocon, nor that ribosome stalling occurs in human neurons under the conditions tested.

We thank the reviewer for this important point. We agree that our data do not directly demonstrate that intermediate, partially unfolded α-synuclein proteoforms physically occlude the translocon or induce ribosome stalling in human neurons. At present, there are no established methods to directly assess such intermediate proteoforms in this context.

To address this limitation, we have performed additional experiments to strengthen evidence for an interaction between pathological α-synuclein species and the Sec61 translocon. Specifically, we assessed co-localisation using the conformation-selective 5G4 antibody, which preferentially recognises misfolded α-synuclein (doi:10.1016/j.nbd.2020.105086). We observed increased co-localisation between 5G4-positive α-synuclein species and Sec61, but not GET1, in human iPSC-derived dopaminergic neurons carrying SNCA triplication compared to isogenic controls (n = 3 independent differentiations). Notably, 5G4-positive puncta were smaller than classical inclusion bodies.

In addition, we quantified the interaction between wild-type and N-terminal deletion α-synuclein mutants and Sec61A in SH-SY5Y cells lacking endogenous α-synuclein (n = 5 independent experiments). These data (new Fig. 4h) show that deletion of residues 32–42 reduces the interaction with Sec61A. We also attempted to generate larger N-terminal deletion mutants; however, these constructs failed to achieve detectable protein expression levels in cells, possibly due to impaired protein stability or rapid degradation, precluding further interaction analyses. Together with our proximity ligation assays (PLA), which show increased signal between pSer129-positive α-synuclein and Sec61 (but not GET1 or EMC), and 3D imaging of postmortem PD substantia nigra and quantification of our PLA experiment in human brain sections (new Fig. 4e–f), these findings provide convergent evidence consistent with an interaction between pathological α-synuclein species and the Sec61 translocon.

It is noteworthy that several biochemical and functional changes observed in our system including increased UFMylation, reduced levels and distribution of Sec61 substrates (e.g. ATP6V0A1 and CTSB), impaired lysosomal acidification and new data showing reduced Cathepsin B activity (**Fig. 7c**) are consistent with disruption of ER translocation. We have revised the text to clearly frame these observations as supportive of, but not definitive for, the proposed mechanism.

We have revised the manuscript to clarify in the limitations of the study that direct demonstration of translocon occlusion will require future methodological advances and explicitly state that the precise α-synuclein species involved and the structural basis of the interaction remain to be determined.

Likewise, proteasome activation is inferred from prior literature and pharmacological manipulation, but proteasome activity was not directly measured in this system. I would therefore ask the authors to slightly tone down their wording in the main text

and Discussion, and to explicitly frame these points as models that are consistent with, but not conclusively demonstrated by, the current data (e.g. avoiding statements that these mechanisms are "demonstrated" or "sufficient," and instead using formulations such as "our findings are consistent with a model in which …").

We thank the reviewer for this suggestion and have revised the manuscript to moderate our conclusions accordingly. Specifically, we have:

(i) replaced "sufficient to" with "can partly" mitigate (page 18, line 405);
(ii) added the statement: "Although we cannot exclude alternative or additional pharmacological mechanisms of action, the use of two mechanistically distinct compounds with a convergent target supports a role for proteasomal clearance in this model…" (page 22–23, lines 504–506);
(iii) clarified in the limitations that "the precise high-resolution structure of the α-synuclein proteoform interacting with Sec61A, as well as direct structural confirmation of this interaction, remain to be determined" (page 23, lines 526–527);
(iv) revised the overall framing to emphasise that our findings are consistent with a model derived from iPSC-based studies of α-synuclein pathology.

<u>**Reviewer #2**</u>

The authors have made a number of revisions and provided additional data to address the issues raised by myself and the other reviewers. Most of the revisions addressed the points I raised and the manuscript is improved.

We thank the reviewer for this positive assessment and for the constructive feedback throughout the review process.

The suggestion to directly monitor translocation by detecting non-translocated precursor (point 3) was addressed with the N-glycosylated substate Cathepsin B, however, the authors refer to the 42 kD pro and pre-pro forms. However as pro-cathepsin-B has two N-glycans, the non-translocated (pre-pro) form will lack the glycans and be smaller, albeit compensated by the uncleaved signal sequence so I suspect 2-3 kD smaller, such a species wasn't obvious in the ESI or SNCA-TRIP blots. As mentioned, it may be that this is rapidly degraded hence the need for proteasome inhibitor to detect the non-translocated precursor. While the reduction in translocated forms of Sec61 substrates, but not GET client, is supportive, the direct demonstration of Sec61-translocation block over reduced translation remains.

We appreciate the reviewer's insightful comment. We agree that detection of the non-translocated precursor form is technically challenging due to its close molecular weight to the signal peptide-containing species. In our system, we did not observe a clearly distinguishable lower molecular weight band corresponding to the non-translocated form, which may reflect rapid degradation, as suggested by the reviewer.

We attempted to address this experimentally. However, human iPSC-derived neurons did not tolerate combined treatment with proteasome inhibitors and ESI, limiting our ability to stabilise and detect this species.

To provide complementary evidence, we assessed functional consequences of impaired translocation by measuring Cathepsin B activity using the Magic Red live-cell assay. These

experiments revealed reduced Cathepsin B activity in dopaminergic neurons with SNCA triplication compared to isogenic controls at day 80 (**Fig. 7c**). This functional readout is consistent with our biochemical data (PLA and immunoblotting) and supports impaired processing of Sec61-dependent substrates such as Cathepsin B.

The suggestion to use of Sec61 inhibitors to investigate whether inhibition of translocation leads to UFMylation looks to be fairly convincing (and an interesting finding) from the UFM blot (Fig. 4H), although if the Rpl26 blot panel is from the same samples, the levels of the mono-UFM band don't seem to match well with the UFM panel.

We thank the reviewer for this observation. It remains unclear whether, in human neurons, additional proteins of similar molecular weight to Rpl26 may also undergo UFMylation, or whether antibody affinity differs between modified and unmodified forms in this human neuronal system. While we consistently observed an increase in higher molecular weight Rpl26 species across independent differentiations, we agree that differences in band intensity relative to total UFM signal may reflect technical or system-specific factors.

I didn't understand the comment on the conA puromycin reduction, "it is not surprising that the difference was not as prominent as with individual substrates as not all glycosylated membrane proteins are translocon substrates.' Does 'translocon' here mean Sec61? Only translocated proteins can be N-glycosylated hence they need a translocon, but they may use different translocases to Sec61.

By "translocon" we were specifically referring to Sec61-dependent substrates. As the reviewer notes, while all N-glycosylated proteins require translocation into the ER, not all are necessarily dependent on the Sec61 complex.

As suggested by reviewer 1, the authors carry out experiments to correlate removal of the potential N-terminal interacting region of Syn with translocation inhibition and interaction with Sec61.
These experiments are very difficult to interpret; the proximity assay shows a marked reduction with the Δ10-20 construct and showed modest loss of translocation inhibition, but then showed similar cross-linking to Sec61α in the IP– although the data are not of highest quality – and I assume this is a denaturing IP (not co-IP as mentioned) to rigorously demonstrate direct crosslinking to Sec61α. In contrast, the Δ21-31and Δ32-42 showed similar interaction in the proximity assay yet even stronger crosslinking in the case of Δ21-31 but no cross-link at all in Δ32-42. Similarly, Δ21-31 construct has strong loss of inhibition while the Δ32-42 is similar to Δ10-20, thus there is no good correlation of Sec61 binding with translocation inhibition. The use of the lysine-based cross-linker DSG is perhaps not the best as there is the additional complication that the lysine residues that form the cross-links are lost in some truncations. Overall, I didn't find this aspect convincing

We thank the reviewer for this detailed and thoughtful critique. To address these concerns, we have performed additional cross-linking experiments and quantified the interaction between wild-type and N-terminal deletion α-synuclein mutants and Sec61A across five independent experiments (new **Fig. 4h**).

These data show that deletion of residues 32–42 consistently reduces interaction with Sec61A, whereas the Δ21–31 mutant shows increased interaction relative to wild type. We agree that interpretation of cross-linking data is complex, particularly given the use of lysine-

reactive crosslinkers and the potential redistribution of lysine residues in truncation mutants. As mentioned above, we also attempted to generate larger N-terminal deletion mutants but these constructs failed to achieve detectable protein expression levels in cells. We have therefore moderated our interpretation and now present these data as supporting, but not definitively establishing, a specific binding interface.

Interestingly, the differential interaction patterns we detected are reflected in downstream functional effects, such as altered ATP6V0A1 levels, with the Δ21–31 mutant (which shows enhanced interaction) further reducing ATP6V0A1 abundance. We have included these observations here for the reviewer to provide additional context while avoiding overinterpretation.

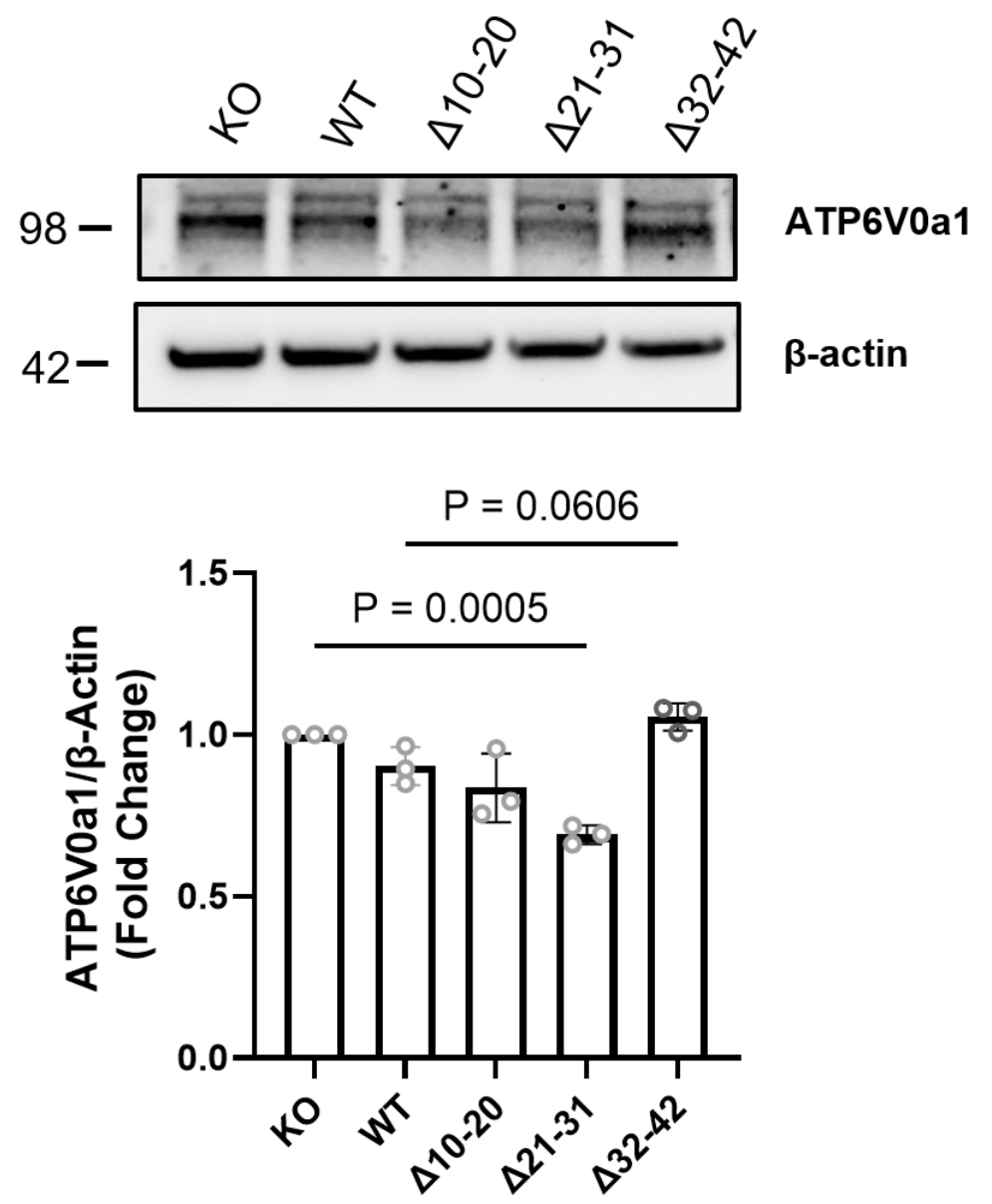

<u>Reviewer #3</u>

The relevance, quality, and soundness of the presented research was already highlighted in my original assessment. All my suggestions to improve the work further have been sufficiently addressed in the revised version. The work should be published.

We thank the reviewer for their positive assessment and recommendation.
